# Dual-Acting Vitamin B_3_‑Melanostatin Neuropeptide Hybrids as Potent Modulators of the Dopamine D_2_ Receptors with Neuroprotective Activity

**DOI:** 10.1021/acs.jmedchem.6c00697

**Published:** 2026-06-18

**Authors:** Beatriz L. Pires-Lima, Sara C. Silva-Reis, Xavier Cruz Correia, Hugo F. Costa-Almeida, Vera M. Costa, Xerardo García-Mera, José Brea, María I. Loza, Marialessandra Contino, Maria Grazia Perrone, Giovanni Graziano, Nuno Vale, José E. Rodríguez-Borges, Ivo E. Sampaio-Dias

**Affiliations:** † LAQV/REQUIMTE, Department of Chemistry and Biochemistry, Faculty of Sciences, 131674 University of Porto, Porto 4169-007, Portugal; ‡ PerMed Research Group, RISE-Health, Faculty of Medicine, 26706 University of Porto, Porto 4200-319, Portugal; § UCIBIO, REQUIMTE, Laboratory of Toxicology, Faculty of Pharmacy, University of Porto, Porto 4050-313, Portugal; ∥ Associate Laboratory i4HB − Institute for Health and Bioeconomy, Faculty of Pharmacy, University of Porto, Porto 4050-313, Portugal; ⊥ Department of Organic Chemistry, Faculty of Pharmacy, University of Santiago de Compostela, Santiago de Compostela E-15782, Spain; # Innopharma Screening Platform. Biofarma Research Group. Centre of Research in Molecular Medicine and Chronic Diseases (CIMUS), University of Santiago de Compostela, Santiago de Compostela E-15782, Spain; ∇ Department of Pharmacy - Pharmaceutical Sciences, 83414 University of Bari “Aldo Moro”, Via E. Orabona 4, Bari 70125, Italy; ○ RISE-Health, Department of Community Medicine, Health Information and Decision (MEDCIDS), Faculty of Medicine, University of Porto, Porto 4200-450, Portugal; ◆ Laboratory of Personalized Medicine, Department of Community Medicine, Health Information and Decision (MEDCIDS), Faculty of Medicine, University of Porto, Porto 4200-450, Portugal

## Abstract

Parkinson’s disease (PD) therapy remains limited by complications of the levodopa regimen, highlighting the need for novel therapeutic strategies to modulate dopaminergic signaling. Melanostatin (MIF-1) has attracted attention as a privileged scaffold for dopamine D_2_ receptor (D_2_R) modulation due to its intrinsic positive allosteric modulatory activity, although improved potency and drug-like properties are required for translation. In this work, nicotinic acid (Nic), a vitamer of vitamin B_3_, was employed as a proline (Pro) bioisostere, generating 12 vitamin B_3_-MIF-1 hybrids. Nicotinoyl-l-leucylglycinamide (**6c**) emerged as a lead compound, promoting a 5.11-fold enhancement of dopamine potency at 0.01 nM and displaying neuroprotective activity at 50–100 μM in dopaminergic-differentiated SH-SY5Y cells. Additionally, Pro-to-Nic substitution showed no cytotoxicity in HepG2, reduced P-glycoprotein-mediated efflux, and preserved β-turn conformational propensity, supporting improved oral drug-like properties. Collectively, these findings identify **6c** as a dual-acting lead and establish Nic as a privileged Pro surrogate for next-generation MIF-1-based PD therapeutics.

## Graphical abstract

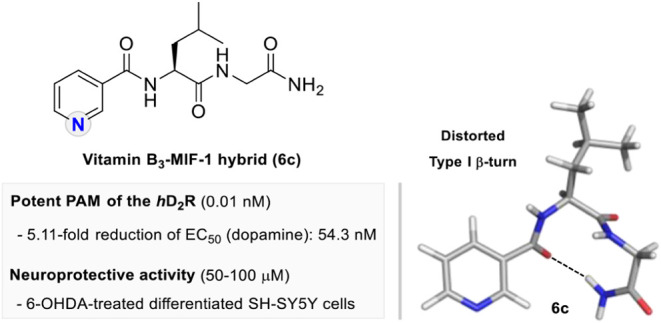

## Introduction

Parkinson’s disease (PD) is widely acknowledged as the most common neurodegenerative movement disorder affecting the central nervous system (CNS) and constitutes a major and growing global health burden.^1^ The disease is pathologically defined by the progressive loss of dopaminergic neurons in the *substantia nigra*, leading to a severe reduction in striatal dopamine (DA) levels.^2^ DA critically regulates basal ganglia circuitry responsible for movement initiation and execution; its loss disrupts motor network homeostasis and culminates in the cardinal motor manifestations collectively referred to as TRAP symptoms: tremor, rigidity, akinesia/bradykinesia, and postural instability.^2^

Current pharmacological management remains centered on DA replacement strategies, most notably the administration of levodopa, the metabolic precursor of DA, typically combined with peripheral decarboxylase inhibitors and monoamine oxidase B or catechol-*O*-methyltransferase inhibitors to enhance central bioavailability.^3–7^ Although these interventions provide substantial symptomatic benefit, their efficacy progressively declines with disease advancement and are frequently accompanied by motor fluctuations, dyskinesias, and other dose-limiting adverse effects.^3–7^ Importantly, conventional DA replacement does not restore physiological dopaminergic signaling dynamics and fails to halt neurodegeneration. These limitations underscore the need for alternative approaches capable of modulating dopaminergic transmission in a more physiologically nuanced manner.

DA signaling is mediated via five G protein-coupled DA receptors (DR), which are grouped into D_1_- and D_2_-like subfamilies according to structural and signaling features; the former includes D_1_R and D_5_R, whereas the latter comprises D_2_R, D_3_R, and D_4_R.^8,9^ Dopaminergic neurotransmission is highly concentration-dependent and temporally regulated. Tonic nanomolar DA levels preferentially engage D_1_-like receptors, whereas phasic micromolar DA release associated with voluntary movement predominantly activates D_2_-like receptors.^8^ Progressive DA depletion in PD disrupts both tonic and phasic signaling; however, signaling pathways requiring higher DA concentrations, particularly D_2_R-mediated responses, become functionally compromised early during dopaminergic decline.^8^ This functional vulnerability positions D_2_R as a particularly attractive target for restoring motor circuit balance under conditions of limited DA availability.^8^

Pharmacological strategies aimed at enhancing D_2_R signaling include direct orthosteric agonism and modulation of receptor responsiveness to endogenous DA.^10–13^ While orthosteric agonists can compensate for DA deficiency, they often lack receptor subtype selectivity and may override physiological spatiotemporal signaling patterns, contributing to adverse effects. In contrast, positive allosteric modulators (PAM) enhance receptor responsiveness without directly activating the receptor, thereby preserving endogenous signaling dynamics and offering a potentially improved safety and tolerability profile.^10–13^

Collectively, these observations establish D_2_R allosteric potentiation as a compelling therapeutic strategy to restore dopaminergic signaling under DA-deficient conditions.^9,11,13–15^ In the context of PD, D_2_R PAM molecules can be used as a pharmacological strategy to amplify residual dopaminergic tone while minimizing overstimulation-associated liabilities.

In this context, melanostatin neuropeptide (MIF-1, Figure 1) represents an interesting scaffold with neuromodulatory activity,^14^ being the only reported endogenous PAM with intrinsic selectivity for the D_2_R.^15–21^

**Figure 1:**
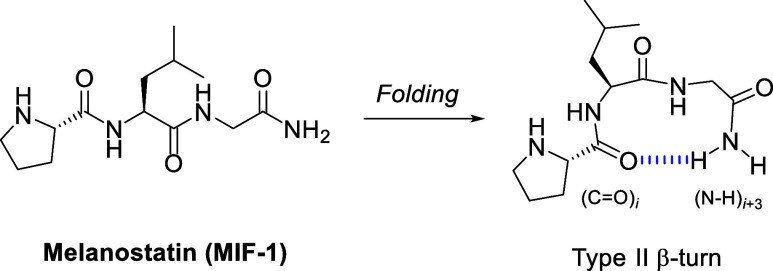
Schematic representation of MIF-1 with an emphasis on its type II β-turn conformation.^18,19,22^

Mechanistically, MIF-1 stabilizes high-affinity receptor conformations, enhancing DA and agonist binding without affecting antagonist binding, receptor expression, DA synthesis, uptake, or metabolism.^11,13,19,22^ This pharmacological profile is consistent with a *bona fide* allosteric mechanism and clearly distinguishes MIF-1 from conventional dopaminergic agents.

Within this framework, MIF-1 represents a privileged molecular scaffold for the development of novel PAM targeting CNS disorders associated with D_2_R dysfunction, including PD,^11^ tardive dyskinesia,^13^ antipsychotic drug-induced dyskinesia,^10^ and depression.^15^

Rational optimization of MIF-1 analogs offers the opportunity to fine-tune allosteric modulation, thereby advancing the development of next-generation PAM of the D_2_R capable of restoring dopaminergic homeostasis with greater therapeutic precision.

## Results and Discussion

### Rational Design

Previous structure–activity relationship studies established that the l-proline (Pro) residue of MIF-1 is tolerant to modification without compromising PAM activity, thereby enabling exploration of alternative scaffolds capable of preserving activity while expanding chemical diversity.^18,23–26^ Guided by this observation, we previously demonstrated that heteroaromatic moieties can successfully replace Pro, affording potent and D_2_R-selective PAM.^19,27,28^ In particular, incorporation of 2-furoic and 3-furoic acid scaffolds maintained or even improved allosteric activity while introducing aromatic character and increased conformational rigidity relative to the native Pro residue.^19,28^

Subsequent bioisosteric replacement of Pro with pyridine-2-carboxylic acid (picolinic acid, Pic) led to the identification of methyl picolinoyl-l-valyl-l-alaninate (**I**, Figure 2), a potent D_2_R PAM.^27^

**Figure 2:**
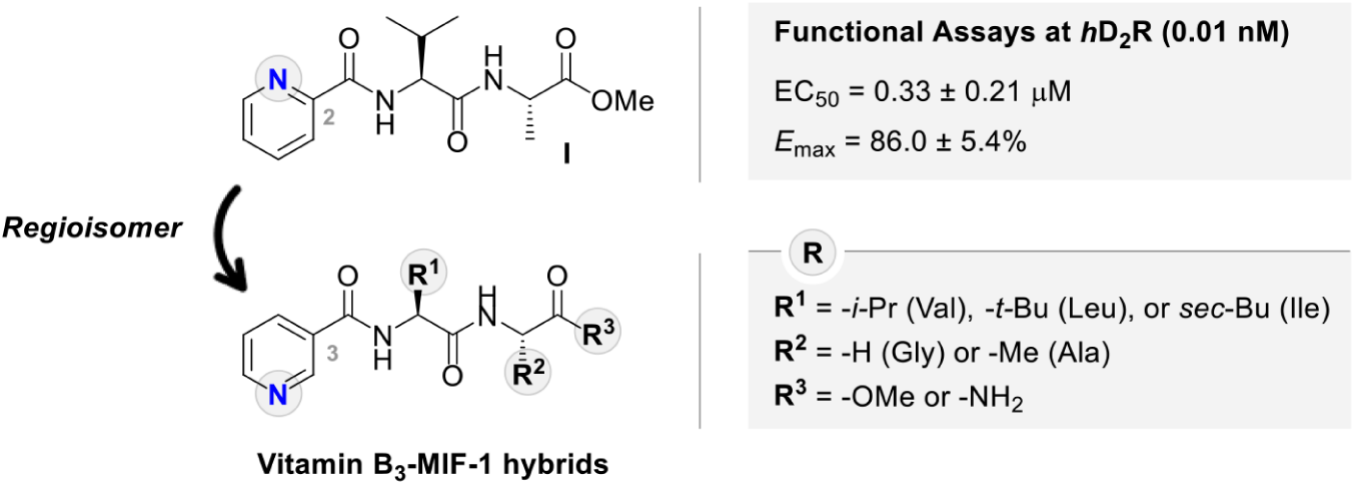
Structure of methyl picolinoyl-l-valyl-l-alaninate (**I**) and the series of Nic-based substitutions explored in this study.

Compound **I** (0.01 nM) exhibited robust PAM activity in functional assays, reducing the half-maximal effective concentration (EC_50_) of DA from 2.92 ± 1.26 μM to 0.33 ± 0.21 μM with a maximal effect (*E*_max_) of 86.0 ± 5.4%, comparable to the PAM effects observed for DA in the presence of MIF-1 (EC_50_ = 0.17 ± 0.07 μM; *E*_max_ = 93.6 ± 4.4%).^27^ These findings validated heteroaromatic Pro bioisosteres as viable scaffolds for the development of MIF-1-based PAM to effectively modulate the D_2_R signaling.

Building on this framework, we sought to interrogate how subtle electronic and spatial perturbations within the pyridine scaffold influence D_2_R PAM activity. To this end, we explored a regioisomer modification by replacing Pic with pyridine-3-carboxylic acid (nicotinic acid, Nic), a well-established vitamer of vitamin B_3_.^29,30^ This substitution repositions the ring nitrogen without altering overall aromatic topology, thereby modulating electronic distribution, hydrogen-bonding orientation, and dipole vector alignment while minimally perturbing steric parameters. From a medicinal chemistry perspective, such a controlled regioisomeric shift provides a mechanistically informative probe of receptor–ligand interactions within the putative allosteric binding pocket.

Beyond its structural relevance, Nic is a NAD^+^/NADP^+^ precursor, central to cellular energy metabolism and redox homeostasis,^29,31^ and has been reported at reduced levels in PD patients and associated with nonmotor symptoms.^31,32^ Furthermore, when used as adjunct therapy to levodopa/carbidopa, Nic has demonstrated neuroprotective and anti-inflammatory effects with potential motor benefits in preliminary clinical studies,^31–34^ thereby providing additional biological context for the exploration of Nic-derived scaffolds in dopaminergic dysfunction.

We hypothesized that Nic-based analogs could retain D_2_R-selective PAM activity through altered electronic interactions at the allosteric interface (Figure 2), while exploring additional neuroprotective properties derived from the vitamin B_3_ moiety. In this context, the conjugation of Nic to the MIF-1 scaffold was envisioned as a strategy to generate dual-acting ligands with enhanced therapeutic potential compared to the parent neuropeptide.

To further delineate structure–activity relationships, targeted side-chain modifications were introduced within the l-valine (Val)/l-alanine (Ala) region of compound **I**. Specifically, Val was interchanged with l-leucine (Leu) or l-isoleucine (Ile) to probe steric tolerance and hydrophobic vector orientation at the central peptide position, while Ala was replaced with glycine (Gly) to evaluate the impact of C_α_ substitution on backbone conformational bias and receptor engagement. Consistent with our earlier findings on compound **I**,^27^ the rational design incorporated both the primary carboxamide and the methyl ester functional groups.

This focused diversification strategy was designed to systematically assess how subtle steric and conformational perturbations influence D_2_R modulation and neuroprotective activity. In total, 12 Nic-based MIF-1 analogs were designed, synthesized, and pharmacologically and biologically evaluated to investigate a dual pharmacological profile, highlighting their potential as next-generation dopaminergic therapeutics for PD.

### Organic Synthesis

Scheme 1 depicts the synthetic route to Nic-based MIF-1 derivatives. The first step comprised the preparation of dipeptides **3­(a–f)**. To this end, *N*-*tert*-butoxycarbonyl (Boc) protected amino acids **1­(x**_**1**_ **–x**_**3**_) reacted with TBTU (*O*-(benzotriazol-1-yl)-*N*,*N*,*N*′,*N*′-tetramethyluronium tetrafluoroborate) as the peptide coupling reagent in the presence of triethylamine (Et_3_N) to generate the corresponding activated benzotriazolyl esters.^18,19^ These activated intermediates were then reacted with the hydrochloride forms of methyl glycinate (H-Gly-OMe·HCl, **2y**_**1**_) or methyl l-alaninate (H-l-Ala-OMe·HCl, **2y**_**2**_), affording dipeptides **3­(a–f)** in good to excellent yields (75–94%, Scheme 1).

**Scheme 1:**
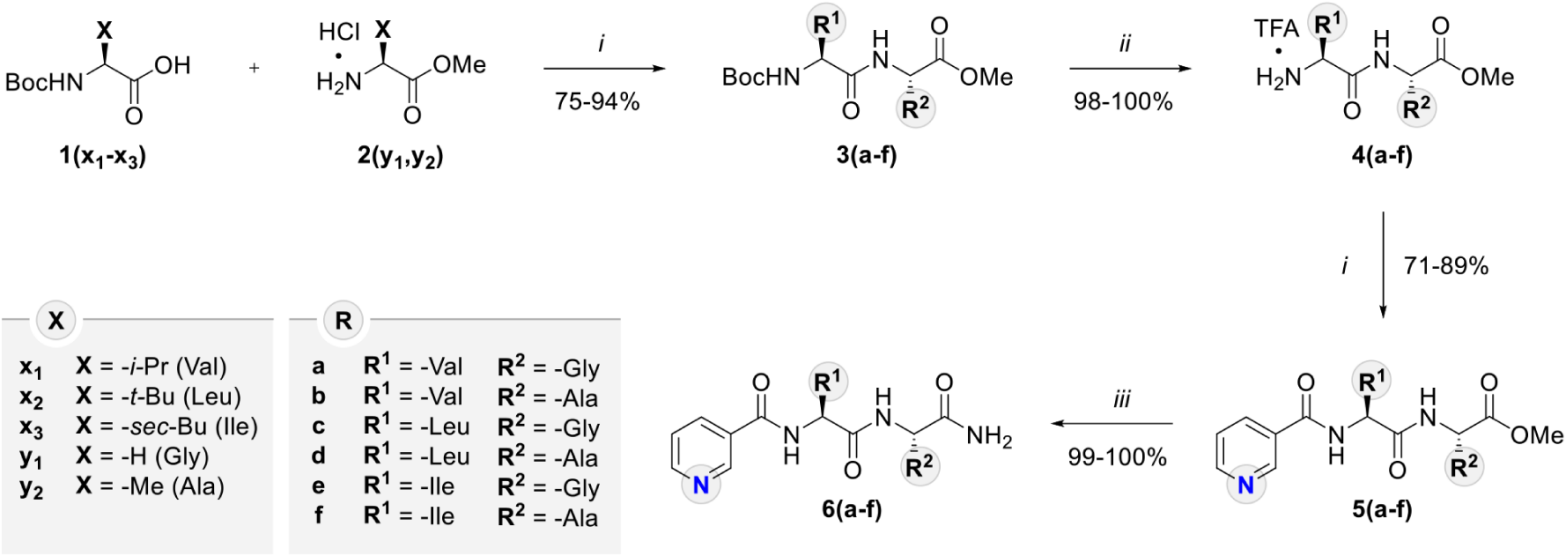
Synthesis of Nic-Based Derivatives **5­(a–f)** and **6­(a–f)** ^a^

a Reagents and conditions: (i) Et_3_N, TBTU, H-Gly-OMe·HCl or H-l-Ala-OMe·HCl, anhydrous CH_2_Cl_2_; (ii) TFA, anhydrous CH_2_Cl_2_; (iii) 7 M ammonia in MeOH.

Next, acidolysis with trifluoroacetic acid (TFA) was used for *N*-Boc deprotection of dipeptides **3­(a–f)**,^18,19^ affording the corresponding dipeptides **4­(a–f)** as ammonium trifluoroacetate salts (98–100%, Scheme 1).

With **4­(a–f)** in hand, peptide coupling with Nic under TBTU-mediated conditions,^18,19^ afforded the hybrid compounds **5­(a–f)** in good yields (71–89%, Scheme 1).

The last reactions comprised ammonolysis to convert the methyl esters **5­(a–f)** into the corresponding primary amides **6­(a–f)**. To this end, compounds **5­(a–f)** were treated with a solution of ammonia (7 M) in methanol (MeOH) until no detection of the starting materials (TLC, *ca*. 48 h).^18,19^ After removal of the volatiles, compounds **6­(a–f)** were obtained in near quantitative yields (99–100%, Scheme 1).

This protocol allowed the preparation of Nic-based MIF-1 derivatives **5­(a–f)** and **6­(a–f)** in 3–4 steps with good global yields (58–81%).

Structural elucidation of the target compounds was performed using 1D nuclear magnetic resonance (NMR) spectroscopy (^1^H, ^13^C­{^1^H}, and DEPT-135) together with high-resolution mass spectrometry (HRMS). Complete experimental procedures and spectral data sets are provided in the Experimental Section and Supporting Information (Figures S1–S44).

### Dopamine Human D_2_R Functional Assay

Prior to pharmacological assays, all peptidomimetics were examined *in silico*,^35^ using established computational filters to assess potential promiscuity, including the propensity for colloidal aggregation and classification as pan-assay interference compounds (PAINS). None of the compounds triggered PAINS alerts or showed predicted aggregation liabilities, supporting their suitability for functional evaluation.

The modulatory activity of Nic-based MIF-1 derivatives **5­(a–f)** and **6­(a–f)** was then evaluated by functional assays using Chinese hamster ovary (CHO) cells transfected with the human D_2_R. Building on a protocol routinely employed in our research group,^18–20^ DA-induced changes in cyclic adenosine monophosphate (cAMP) levels were monitored via homogeneous time-resolved fluorescence (HTRF) assay. This functional readout is particularly suitable for G_i/o_-coupled receptors such as D_2_R: activation of the receptor by DA inhibits adenylyl cyclase, reducing intracellular cAMP.^18–20^ In the presence of a PAM, this inhibitory response becomes more pronounced, allowing us to quantify allosteric enhancement with high sensitivity and excellent assay robustness.

With this preliminary validation in hand, compounds **5­(a–f)** and **6­(a–f)** were initially assessed at 0.01 nM and 1 nM at the D_2_R by measuring their effects on the DA concentration–response curve. Intracellular cAMP levels were quantified via a standard cAMP calibration curve, and the DA concentration–response curve was included in all experiments. For comparative purposes, MIF-1 was evaluated under identical experimental conditions as a positive control.

PAM activity at the D_2_R was assessed by evaluating changes in DA concentration–response curves in the presence and absence of each compound, with EC_50_ and *E*_max_ values determined after normalization to the maximal DA response. A summary of the data is provided in Table S1.

In our assay system, DA alone displayed an EC_50_ of 87.08 ± 24.87 nM. Consistent with previous reports,^18,20^ MIF-1 showed activity only at 1 nM, promoting a 3.68-fold reduction of DA potency (EC_50_ = 23.64 ± 6.73 nM) with no change in efficacy (*E*_max_ = 100%, Figure S46). In contrast, four of the newly prepared Nic-MIF-1 hybrid compounds (**5b**, **5c**, **6c**, and **6f**) exhibited PAM activity at subnanomolar concentrations (0.01 nM), effectively reducing the EC_50_ of DA while largely retaining its maximal efficacy (> 98.9%), except for **5b**, which reached 80.5% of DA *E*_max_.

At 0.01 nM, compounds **5b** and **5c** increased DA potency by more than 2-fold (EC_50_ = 32.18 ± 16.41 and 35.70 ± 8.28 nM, respectively), producing a leftward shift of the DA concentration–response curve (Figure 3). Remarkably, **6c** and **6f** produced even stronger leftward shifts, with EC_50_ values of 17.03 ± 1.35 and 22.89 ± 2.23 nM, corresponding to 5.11-fold and 3.80-fold enhancements of DA potency, respectively (Figure 3). These results are consistent with potent PAM activity at the D_2_R.

**Figure 3:**
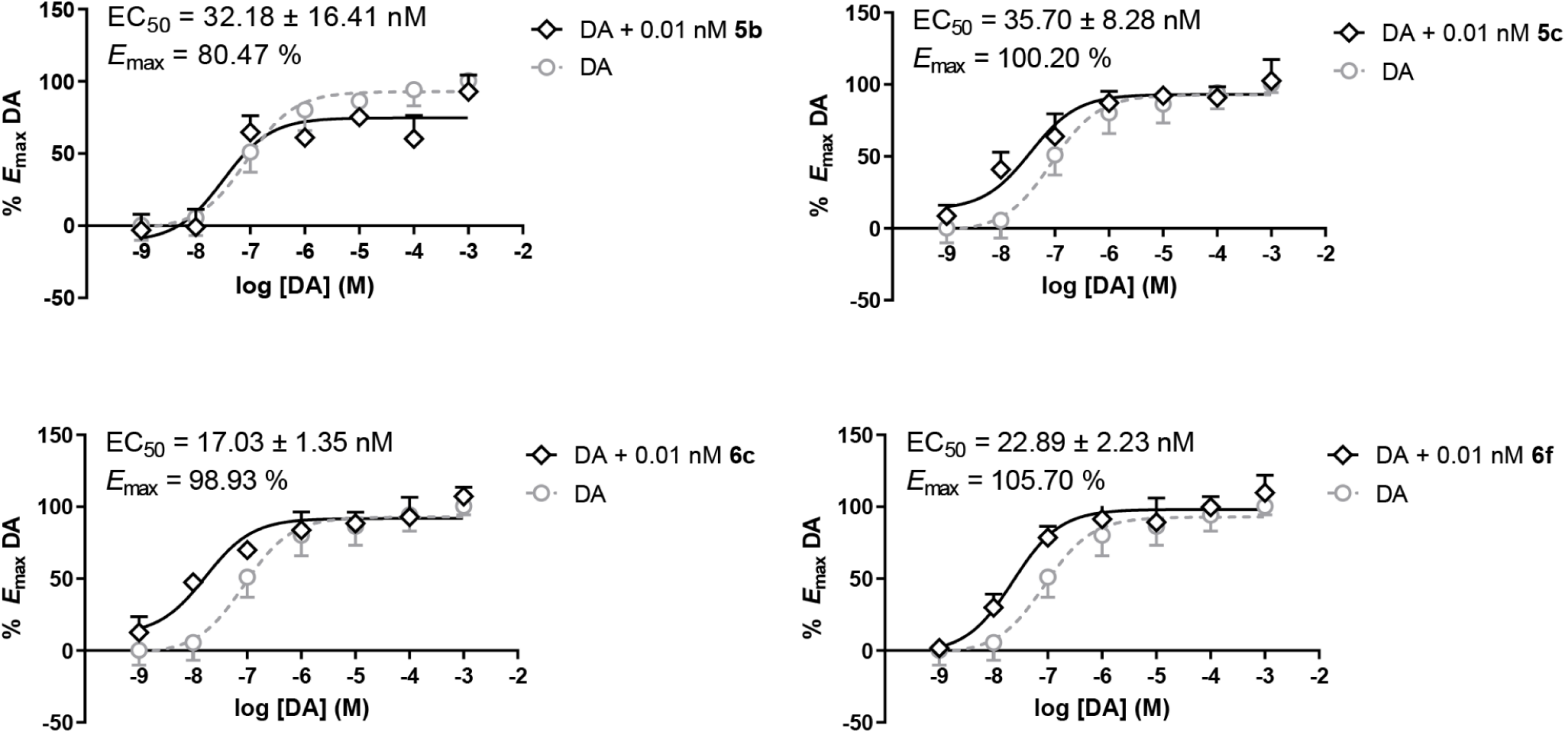
Effect of compounds **5b**, **5c**, **6c**, and **6f** (0.01 nM) on DA concentration–response profiles. The DA curve is included for reference. Data are presented as mean ± standard deviation from three independent experiments carried out in duplicate.

Substitution of the C-terminal methyl ester in **5c** (EC_50_ = 35.70 ± 8.28 nM) with a carboxamide in **6c** (EC_50_ = 17.03 ± 1.35 nM) at 0.01 nM resulted in a 2.1-fold improvement in DA potency (unpaired *t*-test with Welch’s correction; *p* < 0.05), highlighting the critical contribution of the carboxamide functionality to PAM efficacy.

Additionally, to validate the PAM mechanism, all potent modulators (**5b**, **5c**, **6c**, and **6f**) were evaluated for potential intrinsic agonist activity at the D_2_R. In these assays, the compounds were tested in the absence of DA, allowing detection of any direct agonist effect independent of endogenous ligand.

Under these experimental conditions, no intrinsic agonism was observed for any of the compounds. These results support their PAM activity profiles, excluding intrinsic agonist activity or ago-allosteric behavior. Representative data for the most active compound (**6c**) and MIF-1 (control) are shown in Figure 4, with results validated against the DA concentration–response curve.

**Figure 4:**
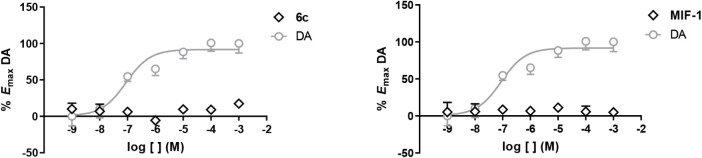
Effect of compound **6c** and MIF-1 in the absence of DA (agonist mode). The DA curve is included for comparison. Data points are presented as mean ± standard deviation and were obtained from three independent experiments carried out in duplicate.

Altogether, these results show that **5b**, **5c**, **6c**, and **6f** exhibit potent activity, modulating the D_2_R at subnanomolar concentrations (0.01 nM), consistent with a PAM mechanism similar to that of MIF-1.

Interestingly, compound **5b**, a regioisomer of compound **I** from the picolinic acid series (Figure 2),^27^ displayed potent PAM activity. Overall, this approach enabled the identification of four Nic-based MIF-1 derivatives with robust PAM activity, demonstrating the effectiveness of this strategy for discovering new allosteric modulators of the D_2_R.

### P-Glycoprotein Interaction

The ability of the synthesized compounds to interact with P-glycoprotein (P-gp), a key adenosine triphosphate-dependent efflux transporter expressed at multiple tissue barriers, including the blood–brain barrier (BBB) interface, was investigated.^36,37^ To this end, the effects of compounds **5b**, **5c**, **6c**, and **6f**, alongside the parent neuropeptide MIF-1 for comparison, were evaluated using a calcein-AM transport inhibition assay in MDCK-MDR1 cells, an epithelial cell line engineered to overexpress human P-gp.^38,39^

Calcein-AM, a nonfluorescent lipophilic probe, readily diffuses across the cell membrane, although it is efficiently extruded by P-gp.^38^ When P-gp activity is inhibited or competitively modulated, calcein-AM accumulates intracellularly and is subsequently hydrolyzed by esterases to yield calcein, a hydrophilic and highly fluorescent molecule that is not a P-gp substrate and therefore remains trapped within the cell.^40^ Consequently, intracellular fluorescence intensity is inversely correlated with P-gp transport activity.

The Nic-based MIF-1 derivatives **5b**, **5c**, **6c**, and **6f** (0.1 to 100 μM) were evaluated in MDCK-MDR1 cells. No cytotoxicity was observed at any tested concentration. All derivatives exhibited negligible inhibition of calcein-AM efflux (< 1%) at 100 μM, suggesting minimal interaction with P-gp under these conditions. These findings indicate that replacing the Pro residue with a Nic heteroaromatic scaffold does not promote P-gp recognition in this *in vitro* model.

### Cytotoxicity Assays in HepG2 and SH-SY5Y Cells

After characterizing the PAM activity, preliminary cytotoxicity assays were conducted on the PAM-active Nic-based MIF-1 derivatives **5b**, **5c**, **6c**, and **6f** using HepG2 cells, a widely used human hepatocellular carcinoma model for assessing general hepatotoxicity.^41,42^ Despite their cancerous origin, HepG2 cells provide a reliable model to screen compounds for general cytotoxicity and are widely adopted for preliminary hepatotoxicity assessment.^41,42^

In this study, HepG2 cells were treated with compounds **5b**, **5c**, **6c**, and **6f** (100 μM) for 72 h, and cytotoxicity was evaluated using the (4,5-dimethylthiazol-2-yl)-2,5-diphenyltetrazolium bromide (MTT) reduction assay as a measure of metabolic activity, through bioreduction of the MTT salt into the corresponding formazan.^43,44^ Cytotoxicity was assessed spectrophotometrically, and cytotoxic effects were inferred from the reduction in the absorbance of the formazans on the control cells, reflecting impaired metabolic activity, namely mitochondrial activity.^43,44^ The hepatotoxicity results obtained for the MTT reduction assay are presented in Figure 5.

**Figure 5:**
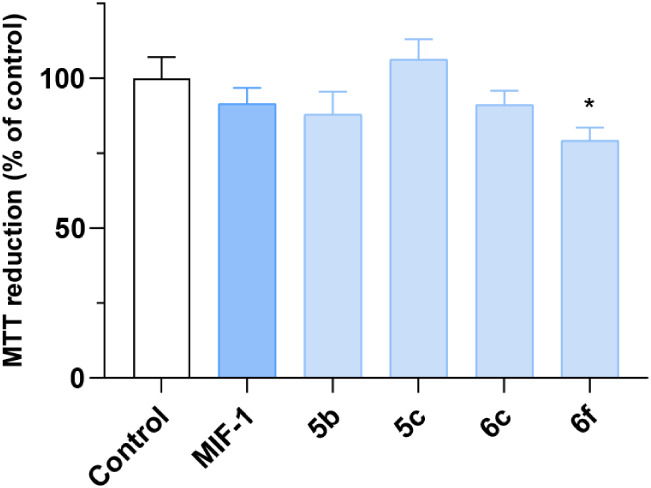
Cytotoxicity in HepG2 cells was assessed after 72 h treatment with compounds **5b**, **5c**, **6c**, and **6f** at 100 μM (1.0% DMSO final concentration) using the MTT reduction assay. Cell viability is reported as a percentage of the control condition (untreated cells) expressed as mean ± standard deviation from two independent experiments. Statistical analysis was performed using one-way ANOVA with Tukey’s *post hoc* test (*p < 0.05 vs control).

Compounds **5b**, **5c**, and **6c** demonstrated a favorable safety profile, with no significant cytotoxicity compared to the control. Conversely, compound **6f** exhibited a mild but statistically significant reduction in viability (79.23 ± 4.11%, *p* < 0.05).

Next, these compounds were evaluated in the human SH-SY5Y neuroblastoma cell line following a differentiation protocol with 12-*O*-tetradecanoylphorbol-13-acetate (TPA) and retinoic acid (RA), which promotes the acquisition of a dopaminergic phenotype,^18,45^ widely recognized as a suitable *in vitro* model for PD research.^18,45,46^ As previously reported by our research group,^18,45^ a 6-day differentiation with RA and TPA enhances the dopaminergic phenotype of these cells and increases resistance to dopaminergic neurotoxicants, along with increasing the expression of TH and DAT.^47,48^

In this study, the neurotoxicity of the active Nic-based PAM derivatives, namely **5b**, **5c**, **6c**, and **6f** (100 μM), the parent neuropeptide MIF-1 (100 μM), and the neurotoxicant 6-hydroxydopamine (6-OHDA, 125 μM), was determined by the MTT reduction assay and complemented by the neutral red (NR) uptake assay.^18,45^ The NR uptake assay was also performed to evaluate lysosomal integrity, as it measures the ability of viable cells to accumulate the NR dye within lysosomes.^18,45,49^ Cytotoxicity in both assays was assessed spectrophotometrically,^18,45,48,49^ with absorbance values inversely proportional to cytotoxicity.

Neurotoxicity results from the MTT reduction (A) and NR uptake (B) assays are shown in Figure 6. Additionally, compound **I** was evaluated using the MTT reduction assay, where it exhibited pronounced cytotoxicity; the corresponding data are provided in the Supporting Information (Figure S47).

**Figure 6:**
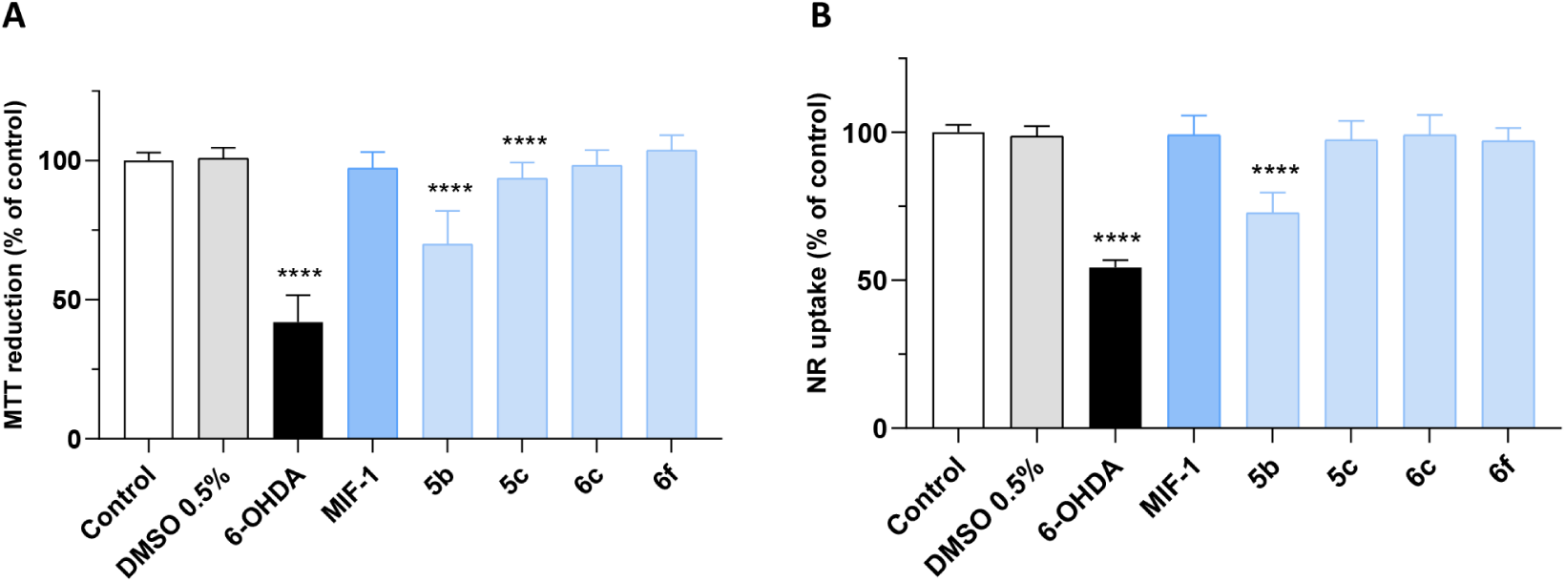
Neurotoxicity assessed by the MTT reduction (A) and NR uptake (B) assays in dopaminergic-differentiated SH-SY5Y cells following a 48-h incubation with compounds **5b**, **5c**, **6c**, **6f**, and MIF-1 at 100 μM (all prepared in DMSO; final well concentration 0.5%), and with 6-OHDA at 125 μM (prepared in PBS). Data from 15–64 wells (A) or 16–24 wells (B) across 4–5 independent experiments are presented as mean ± standard deviation relative to the control condition (PBS). Statistical analysis was performed using one-way ANOVA followed by Tukey’s *post hoc* test (****p < 0.0001 vs control).

In the MTT reduction assay (Figure 6A), cells treated with 6-OHDA (125 μM) exhibited a pronounced cytotoxicity (41.94 ± 9.69%, *p* < 0.0001) relative to the control (phosphate-buffered saline, PBS), consistent with previously reported data,^18–20,45^ confirming the suitability of 6-OHDA as an effective positive control for neurotoxicity (Figure 6A).

Among the Nic-based derivatives, **5c** induced a mild but significant decrease (93.61 ± 5.68%, *p* < 0.0001) in mitochondrial metabolic activity, whereas **5b** showed more pronounced cytotoxicity (69.91 ± 12.03%, *p* < 0.0001), increasing cytotoxicity by more than 30% relative to vehicle control (Figure 6A). In contrast, MIF-1, **6c**, and **6f** did not produce significant cytotoxicity, indicating an absence of cytotoxicity under the tested conditions (Figure 6A).

Consistent with these findings, 6-OHDA also demonstrated significant cytotoxicity in the NR uptake assay (54.34 ± 2.53%, *p* < 0.0001), while MIF-1, **6c**, and **6f** again showed no cytotoxic effects (Figure 6B). The NR uptake assay further confirmed cytotoxicity for compound **5b** (72.80 ± 6.81%, *p* < 0.0001). While **5c** induced a subtle yet significant decrease in cell viability in the MTT reduction assay (93.61 ± 5.68%, *p* < 0.0001), no cytotoxic effect was detected in the NR uptake assay (Figure 6B), indicating an assay-dependent effect and suggesting selective impairment of mitochondrial function without affecting lysosomal integrity.

### Neuroprotection in Differentiated SH-SY5Y Cells

Since 6-OHDA is a well-established pro-oxidant neurotoxin that induces dopaminergic cell death through mitochondrial dysfunction and excessive production of reactive oxygen species (ROS),^50^ it is widely used to model oxidative stress-mediated neurodegeneration *in vitro*.^45,50^ Considering the absence of intrinsic neurotoxicity of **6c** and **6f** (100 μM), together with the reported neuroprotective properties of the Nic moiety incorporated into our design, we next evaluated their neuroprotective potential.

To this end, dopaminergic-differentiated SH-SY5Y cells were exposed to **6c** or **6f** (100 μM) for 1 h before treatment with 6-OHDA (48 h). MIF-1, Nic, and an equimolar mixture of MIF-1 and Nic were also tested at 100 μM for comparison. Compound **I** was excluded from subsequent neuroprotection assays due to its pronounced cytotoxicity in this cell model (Figure S47). All the compounds were solubilized in a mixture of PBS/DMSO, yielding a final well concentration of 0.5% DMSO. 6-OHDA was prepared in PBS to achieve final well concentrations of 25, 50, 75, and 100 μM, ensuring a comprehensive assessment of the neuroprotective effects of the best-performing Nic-based compounds. Cell viability was evaluated using the MTT reduction assay, and the results are shown in Figure 7.

**Figure 7:**
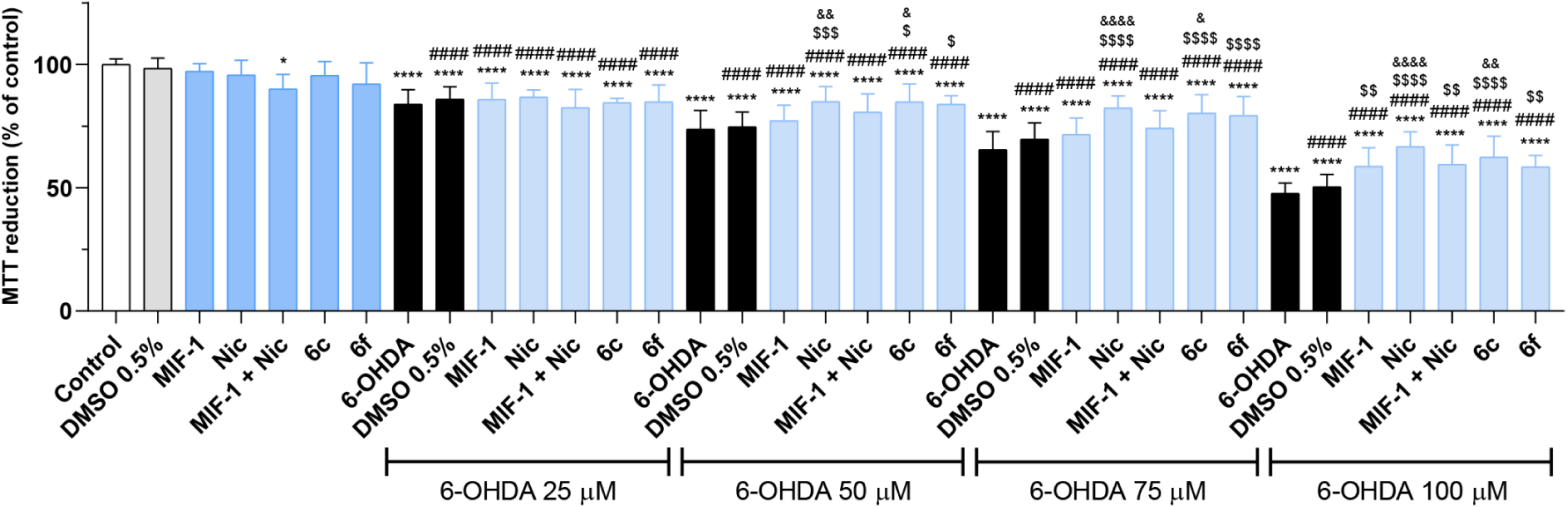
Neuroprotection assessed by the MTT reduction assay in dopaminergic-differentiated SH-SY5Y cells following a 48-h coincubation with MIF-1, Nic, MIF-1 + Nic, **6c**, or **6f** (100 μM) and 6-OHDA at 25, 50, 75, or 100 μM. Data from 9 to 19 wells across 3–5 independent experiments are presented as mean ± standard deviation relative to the control (PBS or DMSO 0.5%). Statistical analysis was performed using one-way ANOVA followed by Tukey’s *post hoc* test (*p < 0.05, and ****p < 0.0001 vs control; ^####^ *p* < 0.0001 vs DMSO 0.5%; ^$^ *p* < 0.05, ^$$^ *p* < 0.01, ^$$$^ *p* < 0.001, and ^$$$$^ *p* < 0.0001 vs 6-OHDA at each concentration; ^&^ *p* < 0.05, ^&&^ *p* < 0.01, and ^&&&&^ *p* < 0.0001 vs DMSO 0.5% + 6-OHDA at each concentration).

As expected, exposure to 6-OHDA alone induced a marked cytotoxicity compared with the untreated control. This effect was concentration-dependent, decreasing cell viability from 83.88 ± 5.87% at 25 μM (*p* < 0.0001) to 47.69 ± 4.24% at 100 μM (*p* < 0.0001), confirming the robustness of the oxidative insult. To validate the experimental conditions, the effect of the solvent was also assessed. No statistical differences were observed when comparing the 6-OHDA groups with the 0.5% DMSO + 6-OHDA groups across all concentrations tested, indicating that 0.5% DMSO did not influence the neurotoxic effect of 6-OHDA. Neuroprotective effects were assessed relative to vehicle controls at each concentration.

Although cotreatment with an equimolar mixture of MIF-1 and Nic induced a slight but statistically significant neurotoxicity (90.09 ± 5.84%, *p* < 0.05), this group was retained in the neuroprotection assays for comparison.

At 25 μM 6-OHDA (Figure 7), none of the tested compounds exhibited statistically significant neuroprotection. Although a general trend toward attenuation of 6-OHDA-induced cytotoxicity was observed for all compounds between 50 and 100 μM, only Nic and **6c** showed statistically significant neuroprotective effects compared to the 0.5% DMSO + 6-OHDA control group (Figure 7).

At higher concentrations of 6-OHDA (50 and 75 μM, Figure 7), both Nic and **6c** improved cell viability by approximately 10% relative to control. Notably, at the highest concentration tested of 6-OHDA (100 μM), Nic and **6c** significantly rescued cell survival by 16% (66.76 ± 5.92%, *p* < 0.0001) and 12% (62.44 ± 8.42%, *p* < 0.01), respectively.

The lack of significant neuroprotection observed in the MIF-1 + Nic control group across all tested 6-OHDA concentrations indicates that simple coincubation of MIF-1 and Nic is insufficient to reproduce the neuroprotective effect of **6c**. Moreover, the absence of neuroprotective activity in **6f**, in contrast to **6c**, suggests that Pro replacement with Nic alone is not sufficient to confer neuroprotective activity. Instead, the peptide sequence appears to play a key role, as substitution of Leu and Gly residues in **6c** with Ile and Ala in **6f** resulted in loss of neuroprotection, potentially reflecting changes in peptide conformational flexibility, intramolecular folding, and hydrophobicity, which may in turn influence membrane interactions and/or recognition by relevant protein targets.

To further support the quantitative findings, the neuronal morphology was examined after 48 h of coincubation. Representative microscopy images illustrating the effects of the different treatments are shown in Figure 8.

**Figure 8:**
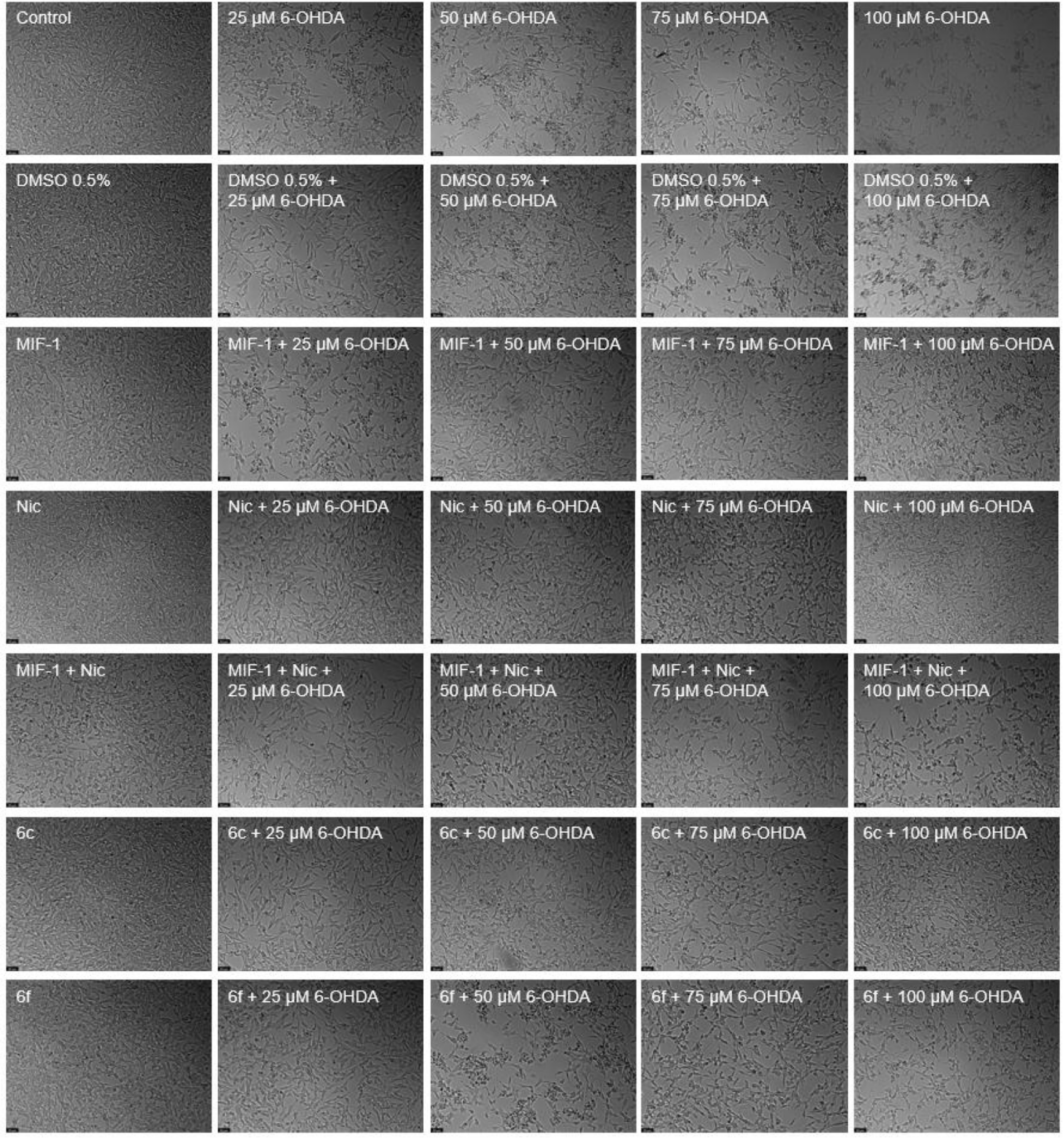
Morphological assessment of neuroprotection against 6-OHDA-induced toxicity in differentiated SH-SY5Y cells. Representative micrographs show cells under the following conditions: Control (PBS), DMSO 0.5%, compounds MIF-1, Nic, MIF-1 + Nic, **6c**, and **6f** (100 μM), and neurotoxicant 6-OHDA (at 25, 50, 75, or 100 μM) alone; DMSO 0.5% + 6-OHDA (at 25, 50, 75, or 100 μM), and cells pretreated with MIF-1, Nic, MIF-1 + Nic, **6c**, and **6f** (100 μM), followed by 6-OHDA (at 25, 50, 75, or 100 μM) exposure. Images were acquired after 48 h of coincubation and are representative of 9–19 wells from 3–5 independent experiments. Scale bar: 50 μm.

The morphological analysis shown in Figure 8 revealed that cells treated with 0.5% DMSO, MIF-1, Nic, **6c**, or **6f** alone did not differ from the control condition, exhibiting the expected differentiated phenotype characterized by high cell density and well-developed neuritic networks. In contrast, exposure to an equimolar mixture of MIF-1 + Nic led to a slight reduction in cell density, in agreement with the metabolic data obtained from the MTT reduction assay.

Conversely, 6-OHDA alone caused a marked reduction in cell density, extensive cell detachment, and the predominance of small, rounded cell bodies, indicative of severe cellular stress, which became more pronounced in a concentration-dependent manner. The 0.5% DMSO + 6-OHDA groups similarly exhibited substantial cell detachment and neurite loss, although a slight preservation of cell density was observed.

Pretreatment with Nic and the hybrid **6c** conferred a visually evident protective effect across all evaluated concentrations. Consistent with the efficacy quantified in the MTT reduction assay, Nic showed the greatest preservation of cellular architecture, with **6c** displaying the next most robust rescue. Although a slight trend toward phenotypic preservation was observed for the remaining compounds, these morphological changes did not translate into statistically significant neuroprotection in the metabolic assays.

These results provide, to our knowledge, the first evidence of neuroprotection associated with MIF-1 derivatives. The combination of the potent PAM activity of **6c** with its neuroprotective effect against 6-OHDA-induced toxicity highlights a unique synergistic profile, suggesting that this Nic-based MIF-1 derivative may offer a dual therapeutic advantage for PD therapy.

### Conformational Analysis

To gain further structure–activity insights and explore the preferential conformations, energy minimizations were performed for compound **6c** in the gas phase using Gaussian 09.^51^

The pyridine ring in **6c** is fully sp^2^-hybridized, with the nitrogen contributing to the aromatic π system via its unhybridized *p* orbital. Planar orientation of the lone pair within the ring enhances nitrogen *s*-character and decreases basicity compared to Pro. While at physiological pH, MIF-1 is protonated,^19,27,52^ consistent with the p*K*_a_ values of pyrrolidinium and pyridinium cations (11.27 and 5.25, respectively),^52,53^ compound **6c** is expected to remain largely neutral. Therefore, only the neutral species were considered in the minimization experiments. The PM6 level of theory was employed in combination with the IEFPCM (integral equation formalism polarizable continuum model) to account for solvation effects in water.^54^ The optimized geometry of **6c** is shown in Figure 9 and Table S2.

**Figure 9:**
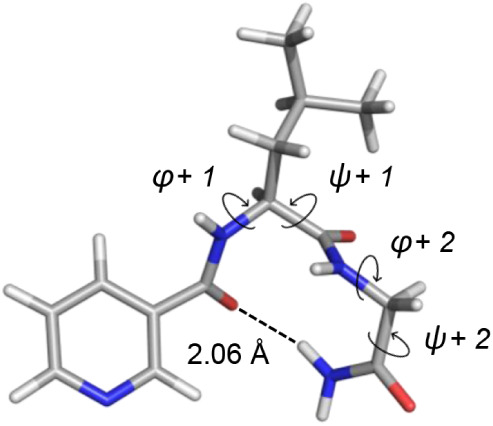
Conformational analysis of **6c** (PM6 level of theory) reveals a distorted type I β-turn in aqueous environment (IEFPCM), stabilized by an intramolecular hydrogen bond (dashed black line).

In compound **6c** (Figure 9), an intramolecular hydrogen bond is formed between the nicotinic acid carbonyl oxygen (C=O)_*i*_ and the *trans*-amide proton of the C-terminus (NH)_*i*+3_, with a donor–acceptor distance (D···A) of 3.05 Å, a hydrogen-acceptor distance (H···A) of 2.06 Å, and an associated bond angle (D–H···A) of 115.39°, suggesting moderate-to-strong interactions. These types of 10-membered pseudocyclic intraturns assisted by a hydrogen bond are collectively known as β-turns.^55,56^ The characteristic (φ, ψ) dihedral angles of the central residues (*i* + 1) and (*i* + 2) vary depending on their type.^56^

For **6c**, the dihedral angles (*i* + 1) and (*i* + 2) obtained were (φ = −58.9°, ψ = −46.1°) and (φ = −94.5°, ψ = +31.4°), respectively, consistent with a type I β-turn conformation (Figure 9), which ideally exhibits dihedral angles around (60°, 30°) and (90°, 0°), respectively.^56^ The ψ angle at *i* + 2 is shifted relative to the canonical value, indicating a mildly distorted type I turn, potentially influenced by local steric and/or electronic contributions arising from the pyridine moiety at position *i*.

The data obtained in this study show that the presence of the pyridine ring at position *i* does not prevent compound **6c** from adopting a β-turn conformation. Despite introducing distinct electronic and steric features relative to the native Pro residue of MIF-1, the Nic moiety is compatible with the formation of a stabilizing intramolecular hydrogen bond and the overall β-turn topology. This observation is of particular relevance to the structure–activity relationships, as β-turn conformations have been proposed as key structural motifs underlying the biological activity of MIF-1 and its analogs.

MIF-1 is suggested to form a type II β-turn, which has been proposed as its active conformation (Figure 1).^22^ However, the strict requirement for this specific secondary structure has been challenged by conformationally constrained analogs that exhibit high potency despite adopting divergent geometries. For instance, Vartak and coworkers demonstrated that derivatives constrained to mimic a type VI β-turn retain significant allosteric activity at the D_2_R.^57^

Furthermore, investigations by Raghavan and coworkers revealed that analogs locked into a polyproline II helix are also pharmacologically active.^58^ Collectively, these findings suggest that the D_2_R allosteric site, which accommodates significant backbone plasticity, may not strictly recognize the type II β-turn, but rather a specific topographical arrangement of pharmacophores.

The ability of **6c** to preserve a (slightly distorted) type I β-turn suggests that substitution of Pro with a heteroaromatic residue can be tolerated without disrupting the conformational framework thought to be important for receptor recognition. Subtle deviations from ideal dihedral angles, such as the ψ angle shift at position *i* + 2, may even contribute to fine-tuning ligand–receptor interactions by modulating the spatial presentation of pharmacophoric groups, potentially influencing potency or selectivity.

Notably, in contrast with **6c**, compounds **5b** and **5c** are structurally incapable of adopting a β-turn conformation (absence of the C-terminal carboxamide). The observed PAM activity of these analogs therefore supports the existence of alternative pharmacologically active conformations for MIF-1 and related peptidomimetics, in agreement with the previous studies from our group^18,19,27,59^ and others.^21,22,57,58,60^

Overall, these results support the notion that incorporating a pyridine ring provides a viable strategy for MIF-1 derivatization while maintaining access to conformations relevant to biological activity. While the minimized structure reflects a low-energy state in implicit solvent, it does not necessarily represent the receptor-bound active conformation. The intrinsic high flexibility of these small peptidomimetics suggests that different conformers may be populated under equilibrium conditions.

## Conclusion

In this work, the rational bioisosteric replacement of the Pro residue in MIF-1 with a pyridine derivative (Nic) yielded highly potent PAM of the D_2_R. A focused library enabled systematic tuning of heteroaromatic positioning, identifying the primary carboxamide as a key pharmacophoric element that enhances both PAM potency and cellular safety.

Among this series, **6c** and **6f** increased dopamine potency by 5.11- and 3.80-fold at 0.01 nM, respectively, with clean PAM profiles devoid of intrinsic agonism. Compound **6c** emerged as the lead, combining superior potency, an improved toxicological profile, and negligible P-gp interaction. Notably, **6c** protected SH-SY5Y cells (dopaminergic phenotype) from 6-OHDA-induced neurotoxicity at concentrations ranging from 50 to 100 μM, representing the first MIF-1 derivative to integrate robust D_2_R PAM activity with neuroprotective effects. Importantly, these results indicate that neuroprotection is not solely dependent on the Pro-to-Nic isosteric modification strategy but also relies on the interplay of the peptide sequence, which critically modulates the overall activity profile.

Computational analyses indicate that **6c** adopts a slightly distorted type I β-turn, preserving the canonical MIF-1 conformational motif while introducing electronic features that may optimize allosteric receptor recognition.

Compared to the parent MIF-1 neuropeptide, the vitamin B_3_-MIF-1 hybrid **6c** displays a distinct biological advantage by successfully combining enhanced D_2_R allosteric modulation with neuroprotective activity. These findings establish Nic as a privileged Pro isostere, validating this strategy for expanding the MIF-1 chemical space, and position **6c** as a promising lead for the development of next-generation dopaminergic therapeutics.

## Experimental Section

### Organic Chemistry: General Data

All chemicals and reagents were of reagent grade and used as received. Boc-l-Leu-OH·H_2_O, Boc-l-Ile-OH, and Boc-l-Val-OH were obtained from NovaBiochem; TBTU was purchased from Bachem (Bubendorf, Switzerland); Nic was purchased from Alfa Aesar (Kandel, Germany); H-Gly-OMe·HCl, H-l-Ala-OMe·HCl, and a 7 M ammonia solution in MeOH were obtained from Fluorochem (Derbyshire, UK); and Et_3_N was from Sigma-Aldrich (Algés, Portugal). Flash column chromatography was carried out using silica gel (Merck 60 Å, 230–240 mesh). Analytical thin-layer chromatography (TLC) was performed on precoated aluminum silica gel plates (Merck 60 F_254_, 0.25 mm). TLC plates were visualized under UV light and/or by staining with phosphomolybdic acid in ethanol, followed by gentle heating. Except for ammonolysis, all reactions were performed under an argon atmosphere.

### Apparatus

Mass spectra were recorded on an LTQ Orbitrap XL hybrid mass spectrometer (Thermo Fisher Scientific, Bremen, Germany) controlled by Xcalibur 2.1.0 and LTQ Tune Plus 2.5.5 (Centro de Materiais da Universidade do Porto, CEMUP). The capillary temperature was set to 275 °C, the capillary voltage to 3.1 kV, and the sheath gas to 6 (arbitrary units as provided by the software settings). The tube lens and capillary voltage were set to 110 and 35 V, respectively.

High-performance liquid chromatography (HPLC) analyses were performed on a Merck-Hitachi LaChrom Elite system equipped with a diode array detector (DAD) at the Department of Chemistry and Biochemistry, Faculty of Sciences, University of Porto (FCUP|DQB – Lab&Services), using a LiChroCART column (250 mm × 4.0 mm, 5 μm). The mobile phase consisted of solvent A (H_2_O containing 0.05% TFA) and solvent B (acetonitrile), using a linear gradient of 1–100% B over 30 min at a flow rate of 1.0 mL/min. All compounds are > 95% pure by HPLC analysis.

NMR spectra were recorded at CEMUP with a Bruker Avance III 400 spectrometer operating at 400.15 and 100.62 MHz for ^1^H and ^13^C­{^1^H}, respectively. Chemical shifts (δ, ppm) for ^1^H and ^13^C­{^1^H} spectra are referenced to the residual protic solvent signals (DMSO-*d*_6_: δ_H_ = 2.50; CDCl_3_: δ_H_ = 7.26)^61^ in the ^1^H NMR spectra and to the deuterated solvents signals (DMSO-*d*_6_: δ_C_ = 39.52; CDCl_3_: δ_C_ = 77.16)^61^ in the ^13^C­{^1^H} NMR spectra. Assignments of proton and carbon resonances follow the three-letter system to specify the proteinogenic α-amino acids (Ala: l-alanine; Gly: glycine; Leu: l-leucine; Ile: l-isoleucine; Val: l-valine) in subscript, denoting the proton (or group of protons) and/or the carbons within a given amino acid residue by numbering from the carbonyl carbon.

Optical rotations were determined using a JASCO P-2000 polarimeter (thermostated) using a sodium lamp and are expressed as follows: (°) (dm^–1^) (g^–1^) and reported as ${[\alpha ]}_{D}^{T}=\mathrm{MV}\pm \mathrm{SD}$ (*c*, solvent), in which *T* is the temperature in degrees Celsius, MV is the mean value of 10 measurements, SD is the standard deviation, and *c* is the concentration in g/100 mL. Melting points were determined using a STUART model SMP1 and are not corrected. Solvents were evaporated using a Büchi RotaVapor R-210.

### General Procedures

#### Peptide Coupling – Synthesis of 3­(**a–f**) and 5­(**a–f**)

In a round-bottom flask, a solution of the appropriate carboxylic acid (1 equiv) in anhydrous CH_2_Cl_2_ (50 mL) was prepared, followed by the addition of Et_3_N (3 equiv) and TBTU (1.05 equiv). After stirring for 20 min at room temperature, the adequate amine (1.05 equiv) was added, and the mixture was further stirred until completion (TLC, *ca.* 2 h). The volatiles were removed under reduced pressure and the residue obtained was dissolved in EtOAc (100 mL), transferred into a separatory funnel, and washed with a saturated aqueous solution of NaHCO_3_ (3 × 100 mL). Then, the organic layer was dried over anhydrous Na_2_SO_4_, filtered, concentrated under reduced pressure, and the crude product was purified as specified below for each compound.

#### Removal of *N*-Boc Protecting Group – Synthesis of 4­(**a–f**)

In a round-bottom flask, a solution of the appropriate *N*-Boc carbamate (1 equiv) in anhydrous CH_2_Cl_2_ (30 mL) was prepared, followed by the addition of TFA (30 equiv). The mixture was left under magnetic stirring at room temperature until completion (TLC, *ca.* 2 h). After completion, the volatiles were removed under reduced pressure, affording the corresponding ammonium trifluoroacetate salts as pure compounds.

#### Ammonolysis – Synthesis of 6­(**a–f**)

In a round-bottom flask, the appropriate methyl ester (1 equiv) was dissolved in methanolic ammonia solution (7 M, 25 mL), and the mixture was stirred at room temperature until completion (TLC, *ca*. 48 h). Volatiles were then removed under reduced pressure to afford the corresponding pure primary carboxamide without further purification.

**Methyl (** ***tert*** **-butoxycarbonyl)-** **l** **-valylglycinate (3a).** Following the general protocol for peptide coupling, Boc-l-Val-OH (1.6742 g, 7.7056 mmol) reacted with Et_3_N (3.22 mL, 23.1 mmol) and TBTU (2.5979 g, 8.0909 mmol), followed by the addition of H-Gly-OMe·HCl (1.0158 g, 8.0909 mmol). After the workup, the resulting residue was purified by column chromatography using EtOAc/Hex (2:1) as eluent, affording **3a** (1.9996 g) as a white solid. The analytical and spectroscopic data were in perfect agreement with the previously reported.^62^ **Yield:** 90%. **R**_***f***_: 0.62 in EtOAc/Hex (3:2).

**Methyl (** ***tert*** **-butoxycarbonyl)-** **l** **-valyl-** **l** **-alaninate (3b).** Following the general protocol for peptide coupling, Boc-l-Val-OH (1.5108 g, 6.9536 mmol) reacted with Et_3_N (2.91 mL, 20.9 mmol) and TBTU (2.3444 g, 7.3013 mmol), followed by the addition of H-l-Ala-OMe·HCl (1.0191 g, 7.3013 mmol). After the workup, the resulting residue was purified by column chromatography using CH_2_Cl_2_/MeOH (10:1) as eluent, affording **3b** (1.8322 g) as a white solid. The analytical and spectroscopic data were in perfect agreement with the previously reported.^59^ **Yield:** 87%. **R**_***f***_: 0.79 in CH_2_Cl_2_/MeOH (10:1).

**Methyl (** ***tert*** **-butoxycarbonyl)-** **l** **-leucylglycinate (3c).** Following the general protocol for peptide coupling, Boc-l-Leu-OH·H_2_O (1.8612 g, 8.0470 mmol) reacted with Et_3_N (3.32 mL, 24.1 mmol) and TBTU (2.7130 g, 8.4494 mmol), followed by the addition of H-Gly-OMe·HCl (1.0608 g, 8.4492 mmol). After the workup, the resulting residue was precipitated, using Hex (30 mL), filtered under reduced pressure, and washed with cold Hex, affording **3c** (1.8249 g) as a white solid. The analytical and spectroscopic data were in perfect agreement with the previously reported.^59,63^ **Yield:** 75%. **R**_***f***_: 0.89 in EtOAc.

**Methyl (** ***tert*** **-butoxycarbonyl)-** **l** **-leucyl-** **l** **-alaninate (3d).** Following the general protocol for peptide coupling, Boc-l-Leu-OH·H_2_O (2.0101 g, 8.6908 mmol) reacted with Et_3_N (3.63 mL, 26.1 mmol) and TBTU (2.9301 g, 9.1253 mmol), followed by the addition of H-l-Ala-OMe·HCl (1.2737 g, 9.1253 mmol). After the workup, the resulting residue was precipitated, using Hex (30 mL), filtered under reduced pressure, and washed with cold Hex, affording **3d** (2.4198 g) as a white solid. The analytical and spectroscopic data were in perfect agreement with the previously reported.^64^ **Yield:** 88%. **R**_***f***_: 0.81 in EtOAc.

**Methyl (** ***tert*** **-butoxycarbonyl)-** l-isoleucylglycinate (3e). Following the general protocol for peptide coupling, Boc-l-Ile-OH (1.6520 g, 7.1425 mmol) reacted with Et_3_N (2.98 mL, 21.4 mmol) and TBTU (2.4081 g, 7.4996 mmol), followed by the addition of H-Gly-OMe·HCl (0.9416 g, 7.500 mmol). After the workup, the resulting residue was purified by column chromatography using EtOAc/Hex (3:2) as eluent, affording **3e** (2.0301 g) as a white solid. The analytical and spectroscopic data were in perfect agreement with the previously reported.^65^ **Yield:** 94%. **R**_***f***_: 0.71 in EtOAc/Hex (3:2).

**Methyl (** ***tert*** **-butoxycarbonyl)-** l-isoleucyl- **l** **-alaninate (3f).** Following the general protocol for peptide coupling, Boc-l-Ile-OH (1.6833 g, 7.2779 mmol) reacted with Et_3_N (3.04 mL, 21.8 mmol) and TBTU (2.4537 g, 7.6418 mmol), followed by the addition of H-l-Ala-OMe·HCl (1.0666 g, 7.6418 mmol). After the workup, the resulting residue was precipitated using Hex (30 mL), filtered under reduced pressure, and washed with cold Hex, affording **3f** (1.9113 g) as a white solid. The analytical and spectroscopic data were in perfect agreement with the previously reported.^65^ **Yield:** 83%. **R**_***f***_: 0.77 in EtOAc.

**Methyl** **l** **-valylglycinate trifluoroacetate (4a).** Following the general protocol for removal of the *N*-Boc protecting group, carbamate **3a** (1.9414 g, 6.7330 mmol) reacted with TFA (15.46 mL, 202.0 mmol). After the workup, **4a** (2.0350 g) was obtained as a pale-yellow oil. **Yield:** 100%. **R**_***f***_: 0.00 in EtOAc. ^**1**^ **H NMR** (DMSO-*d*_6_, 400 MHz) δ ppm: 8.89 (t, *J* = 5.8 Hz, 1H, OCONH), 8.16 (br s, 3H, ^+^NH_3_), [4.03 (dd, *J* = 17.4, 6.0 Hz, 1H), 3.90 (dd, *J* = 17.4, 5.7 Hz, 1H), H_Gly_-2], 3.65 (s, 3H, CO_2_CH_3_), 3.68 (d, *J* = 5.8 Hz, 1H, H_Val_-2), 2.16–2.04 (m, 1H, H_Val_-3), [0.97 (d, *J* = 6.9 Hz), 0.96 (d, *J* = 6.9 Hz), 6H, H_Val_-4]. ^**13**^ **C­{** ^**1**^ **H} NMR and DEPT-135** (DMSO-*d*_6_, 101 MHz) δ ppm: [169.8 (C), 168.5 (C), CONH + CO_2_CH_3_], 57.3 (CH, C_Val_-2), 51.8 (CH_3_, OCH_3_), 40.6 (CH_2_, C_Gly_-2), 29.8 (CH, C_Val_-3), [18.1 (CH_3_), 17.6 (CH_3_), C_Val_-4].

**Methyl** **l** **-valyl-** **l** **-alaninate trifluoroacetate (4b).** Following the general protocol for removal of the *N*-Boc protecting group, carbamate **3b** (1.5002 g, 4.9615 mmol) reacted with TFA (11.40 mL, 148.9 mmol). After the workup, the resulting residue was precipitated, using Et_2_O (30 mL), filtered under reduced pressure, and washed with Et_2_O, affording **4b** (1.5687 g) as a white solid. The analytical and spectroscopic data were in perfect agreement with the previously reported.^59^ **Yield:** 100%. **R**_***f***_: 0.00 in EtOAc.

**Methyl** **l** **-leucylglycinate trifluoroacetate (4c).** Following the general protocol for removal of the *N*-Boc protecting group, carbamate **3c** (1.7543 g, 5.8018 mmol) reacted with TFA (13.33 mL, 174.1 mmol). After the workup, **4c** (1.8350 g) was obtained as a brown oil. The analytical and spectroscopic data were in perfect agreement with the previously reported.^59,63^ **Yield:** 100%. **R**_***f***_: 0.00 in EtOAc.

**Methyl** **l** **-leucyl-** **l** **-alaninate trifluoroacetate (4d).** Following the general protocol for removal of the *N*-Boc protecting group, carbamate **3d** (1.7111 g, 5.4080 mmol) reacted with TFA (12.42 mL, 162.2 mmol). After the workup, the resulting residue was precipitated, using Et_2_O (30 mL), filtered under reduced pressure, and washed with Et_2_O, affording **4d** (1.7862 g) as a white solid. **Yield:** 100%. **R**_***f***_: 0.00 in EtOAc. ^**1**^ **H NMR** (DMSO-*d*_6_, 400 MHz) δ ppm: 8.96 (d, *J* = 6.9 Hz, 1H, OCONH), 8.25 (br s, 3H, ^+^NH_3_), 4.35 (p, *J* = 7.26 Hz, 1H, H_Ala_-2), 3.78 (t, *J* = 7.2 Hz, 1H, H_Leu_-2), 3.63 (s, 3H, CO_2_CH_3_), 1.71 (dp, *J* = 13.2, 6.6 Hz, 1H, H_Leu_-4), 1.55 (m, 2H, H_Leu_-3), 1.32 (d, *J* = 7.3 Hz, 3H, H_Ala_-3), [0.91 (d, *J* = 6.5 Hz), 0.89 (d, *J* = 6.5 Hz), 6H, H_Leu_-5]. ^**13**^ **C­{** ^**1**^ **H} NMR and DEPT-135** (DMSO-*d*_6_, 101 MHz) δ ppm: [172.4 (C), 168.9 (C), OCONH + CO_2_CH_3_], 52.0 (CH, C_Leu_-2), 50.7 (CH_3_, CO_2_CH_3_), 47.7 (CH, C_Ala_-2), 40.2 (CH_2_, C_Leu_-3), 23.4 (CH, C_Leu_-4), [22.5 (CH_3_), 22.1 (CH_3_), C_Leu_-5], 16.7 (CH_3_, C_Ala_-3).

**Methyl** l-isoleucylglycinate trifluoroacetate (4e). Following the general protocol for removal of the *N*-Boc protecting group, carbamate **3e** (1.9782 g, 6.5423 mmol) reacted with TFA (15.03 mL, 196.3 mmol). After the workup, **4e** (2.0692 g) was obtained as a pale-yellow oil. **Yield:** 100%. **R**_***f***_: 0.00 in EtOAc. ^**1**^ **H NMR** (DMSO-*d*_6_, 400 MHz) δ ppm: 8.87 (t, *J* = 5.0 Hz, 1H, CONH), 8.17 (br s, 3H, ^+^NH_3_), [4.03 (dd, *J* = 17.4, 6.0 Hz, 1H), 3.88 (dd, *J* = 17.4, 5.6 Hz, 1H), H_Gly_-2], 3.72–3.67 (m, 1H, H_Ile_-2), 3.64 (s, 3H, CO_2_CH_3_), 1.94–1.77 (m, 1H, H_Ile_-3), [1.52 (dqd, *J* = 14.6, 7.4, 3.9 Hz, 1H), 1.17 (ddq, *J* = 14.2, 9.3, 7.2 Hz, 1H), H_Ile_-4], 0.93 (d, *J* = 6.9 Hz, 3H, H_Ile_-6), 0.87 (t, *J* = 7.4 Hz, 3H, H_Ile_-5). ^**13**^ **C­{** ^**1**^ **H} NMR and DEPT-135** (DMSO-*d*_6_, 101 MHz) δ ppm: [169.8 (C), 168.4 (C), CONH + CO_2_CH_3_], 56.5 (CH, C_Ile_-2), 51.8 (CH_3_, CO_2_CH_3_), 40.6 (CH_2_, C_Gly_-2), 26.3 (CH, C_Ile_-3), 24.0 (CH_2_, C_Ile_-4), 14.3 (CH_3_, C_Ile_-6), 11.2 (CH_3_, C_Ile_-5).

**Methyl** l-isoleucyl- **l** **-alaninate trifluoroacetate (4f).** Following the general protocol for removal of the *N*-Boc protecting group, carbamate **3f** (1.4002 g, 4.4311 mmol) reacted with TFA (10.18 mL, 132.9 mmol). After the workup, the resulting residue was precipitated, using Et_2_O (30 mL), filtered under reduced pressure, and washed with Et_2_O, affording **4f** (1.4343 g) as a white solid. **Yield:** 98%. **R**_***f***_: 0.00 in EtOAc. ^**1**^ **H NMR** (DMSO-*d*_6_, 400 MHz) δ ppm: 8.87 (d, *J* = 6.7 Hz, 1H, CONH), 8.21 (br s, 3H, ^+^NH_3_), 4.35 (p, *J* = 7.3 Hz, 1H, H_Ala_-2), 3.78 (t, *J* = 7.2 Hz, 1H, H_Ile_-2), 3.63 (s, 3H, CO_2_CH_3_), [1.83 (ddt, *J* = 12.8, 6.7, 3.7 Hz, 1H), 1.51 (dtd, *J* = 14.9, 7.5, 3.8 Hz, 1H), H_Ile_-4], 1.24–1.09 (m, 1H, H_Ile_-3), 1.32 (d, *J* = 7.3 Hz, 3H, H_Ala_-3), 0.93 (d, *J* = 6.9 Hz, 3H, H_Ile_-6), 0.87 (d, *J* = 6.9 Hz, 3H, H_Ile_-5). ^**13**^ **C­{** ^**1**^ **H} NMR and DEPT-135** (DMSO-*d*_6_, 101 MHz) δ ppm: [172.4 (C), 167.8 (C), CONH + CO_2_CH_3_], 56.4 (CH, C_Ile_-2), 52.0 (CH_3_, CO_2_CH_3_), 47.7 (CH, C_Ala_-2), 36.2 (CH, C_Ile_-3), 23.9 (CH_2_, C_Ile_-4), 16.7 (CH_3_, C_Ile_-6), 14.3 (CH_3_, C_Ala_-3), 11.1 (CH_3_, C_Ile_-5).

**Methyl nicotinoyl-** **l** **-valylglycinate (5a).** Following the general protocol for peptide coupling, Nic (0.3080 g, 2.502 mmol) reacted with Et_3_N (1.04 mL, 7.50 mmol) and TBTU (0.8435 g, 2.627 mmol), followed by the addition of **4a** (0.7941 g, 2.627 mmol). After the workup, the resulting residue was purified by column chromatography using Hex/Acetone (3:1) as eluent, affording **5a** (0.5700 g) as a white solid. **Yield:** 78%. **m.p.**: 161–164 °C. **R**_***f***_: 0.55 in CH_2_Cl_2_/MeOH. ${[\alpha ]}_{D}^{21}$ : +6.31 ± 0.08 (*c*1.000, CHCl_3_). ^**1**^ **H NMR** (CDCl_3_, 400 MHz) δ ppm: 9.04 (d, *J* = 1.6 Hz, 1H, H-2), 8.73 (dd, *J* = 4.8, 1.6 Hz, 1H, H-6), 8.11 (dt, *J* = 7.9, 1.9 Hz, 1H, H-4), 7.40–7.34 (m, 1H, H-5), 7.15 (d, *J* = 8.5 Hz, 1H, CONH), 6.92 (t, *J* = 4.8 Hz, 1H, CONH), 4.58 (dd, *J* = 8.5, 7.2 Hz, 1H, H_Val_-2), [4.15 (dd, *J* = 18.2, 5.7 Hz, 1H), 3.98 (dd, *J* = 18.2, 5.1 Hz, 1H), H_Gly_-2], 3.74 (s, 3H, CO_2_CH_3_), 2.24 (dq, *J* = 13.7, 6.8 Hz, 1H, H_Val_-3), [1.04 (d, *J* = 6.7 Hz), 1.04 (d, *J* = 6.8 Hz), 6H, H_Val_-4]. ^**13**^ **C­{** ^**1**^ **H} NMR and DEPT-135** (CDCl_3_, 101 MHz) δ ppm: [171.9 (C), 170.1 (C), CONH + CO_2_CH_3_], 166.0 (C, CONH), 152.3 (CH, C-2), 148.5 (CH, C-4), 135.4 (CH, C-6), 129.9 (C, C-3), 123.5 (CH, C-5), 59.2 (CH, C_Val_-2), 52.4 (CH_3_, CO_2_CH_3_), 41.3 (CH_2_, C_Gly_-2), 31.3 (CH, C_Val_-3), [19.3 (CH_3_), 18.7 (CH_3_), C_Val_-4]. **HRMS** (ESI-TOF) *m*/*z*: [M + H]^+^ Calcd for C_14_H_20_N_3_O_4_^+^: 294.14483; Found: 294.14484.

**Methyl nicotinoyl-** **l** **-valyl-** **l** **-alaninate (5b).** Following the general protocol for peptide coupling, Nic (0.3104 g, 2.521 mmol) reacted with Et_3_N (1.05 mL, 7.56 mmol) and TBTU (0.8261 g, 2.647 mmol), followed by the addition of **4b** (0.8372 g, 2.647 mmol). After the workup, the resulting residue was purified by column chromatography using EtOAc as eluent, affording **5b** (0.6882 g) as a white solid. **Yield:** 89%. **m.p.**: 148–151 °C. **R**_***f***_: 0.58 in EtOAc. ${[\alpha ]}_{D}^{18}$ : −10.33 ± 0.07 (*c*1.025, CHCl_3_). ^**1**^ **H NMR** (CDCl_3_, 400 MHz) δ ppm: 9.05 (d, *J* = 1.5 Hz, 1H, H-2), 8.72 (dd, *J* = 4.9, 1.7 Hz, 1H, H-6), 8.12 (ddd, *J* = 8.0, 2.3, 1.7 Hz, 1H, H-4), 7.35 (ddd, *J* = 7.9, 4.8, 0.9 Hz, 1H, H-5), 7.30 (d, *J* = 8.6 Hz, 1H, CONH), 7.06 (d, *J* = 7.3 Hz, 1H, CONH), 4.59 (m, 2H, H_Val_-2 + H_Ala_-2), 3.74 (s, 3H, CO_2_CH_3_), 2.20 (h, *J* = 6.8 Hz, 1H, H_Val_-3), 1.38 (d, *J* = 7.2 Hz, 3H, H_Ala_-3), [1.04 (d, *J* = 6.1 Hz), 1.02 (d, *J* = 6.0 Hz), 6H, H_Val_-4]. ^**13**^ **C­{** ^**1**^ **H} NMR and DEPT-135** (CDCl_3_, 101 MHz) δ ppm: [173.2 (C), 171.0 (C), 165.7 (C), CO_2_CH_3_ + 2 × CONH], 152.5 (CH, C-2), 148.5 (CH, C-4), 135.3 (CH, C-6), 129.9 (C, C-3), 123.5 (CH, C-5), 59.0 (CH, C_Ala_-2), 52.6 (CH_3_, CO_2_CH_3_), 48.3 (CH, C_Val_-2), 31.8 (CH, C_Val_-3), [19.2 (CH_3_), 18.6 (CH_3_), C_Val_-4], 18.1 (CH_3_, C_Ala_-3). **HRMS** (ESI-TOF) *m*/*z*: [M + H]^+^ Calcd for C_15_H_22_N_3_O_4_^+^: 308.16048; Found: 308.16049.

**Methyl nicotinoyl-** **l** **-leucylglycinate (5c).** Following the general protocol for peptide coupling, Nic (0.2692 g, 2.187 mmol) reacted with Et_3_N (0.91 mL, 6.6 mmol) and TBTU (0.7372 g, 2.296 mmol), followed by the addition of **4c** (0.7262 g, 2.296 mmol). After the workup, the resulting residue was purified by column chromatography using EtOAc as eluent, affording **5c** (0.5530 g) as a white solid. **Yield:** 82%. **m.p.**: 148–151 °C. **R**_***f***_: 0.22 in EtOAc. ${[\alpha ]}_{D}^{19}$ : −15.87 ± 0.11 (*c*0.995, CHCl_3_). ^**1**^ **H NMR** (CDCl_3_, 400 MHz) δ ppm: 9.06 (br s, 1H, H-2), 8.68 (d, *J* = 4.7 Hz, 1H, H-6), 8.12 (d, *J* = 8.0 Hz, 1H, H-4), 7.63 (d, *J* = 5.7 Hz, 1H, CONH), 7.38–7.31 (m, 2H, H-5 + CONH), 4.82–4.77 (m, 1H, H_Leu_-2), [4.07 (dd, *J* = 18.1, 5.6 Hz, 1H), 3.97 (dd, *J* = 18.1, 5.3 Hz, 1H), H_Gly_-2], 3.71 (s, 3H, CO_2_CH_3_), 1.76–1.63 (m, 3H, H_Leu_-3 + H_Leu_-4), [0.90 (d, *J* = 6.3 Hz), 0.88 (d, *J* = 5.2 Hz), 6H, H_Leu_-5]. ^**13**^ **C­{** ^**1**^ **H} NMR and DEPT-135** (CDCl_3_, 101 MHz) δ ppm: [172.8 (C), 170.1 (C), 165.9 (C), 2 × CONH + CO_2_CH_3_], 152.4 (CH, C-2), 148.6 (CH, C-4), 135.5 (CH, C-6), 129.6 (C, C-3), 123.5 (CH, C-5), 52.5 (CH_3_, CO_2_CH_3_), 52.2 (CH, C_Leu_-2), 41.3 (CH_2_, C_Gly_-2), 41.0 (CH_2_, C_Leu_-3), 25.0 (CH, C_Leu_-4), [22.9 (CH_3_), 22.2 (CH_3_), C_Leu_-5]. **HRMS** (ESI-TOF) *m*/*z*: [M + H]^+^ Calcd for C_15_H_22_N_3_O_4_^+^: 308.16048; Found: 308.16043.

**Methyl nicotinoyl-** **l** **-leucyl-** **l** **-alaninate (5d).** Following the general protocol for peptide coupling, Nic (0.3454 g, 2.806 mmol) reacted with Et_3_N (1.17 mL, 8.42 mmol) and TBTU (0.9194 g, 2.946 mmol), followed by the addition of **4d** (0.9731 g, 2.946 mmol). After the workup, the resulting residue was precipitated using Hex (30 mL), filtered under reduced pressure, and washed with cold Hex, affording **5d** (0.7228 g) as a white solid. **Yield:** 80%. **m.p.**: 144–147 °C. **R**_***f***_: 0.58 in EtOAc. ${[\alpha ]}_{D}^{21}$ : −26.95 ± 0.11 (*c*1.030, CHCl_3_). ^**1**^ **H NMR** (CDCl_3_, 400 MHz) δ ppm: 9.04 (d, *J* = 1.6 Hz, 1H, H-2), 8.70 (dd, *J* = 4.9, 1.7 Hz, 1H, H-6), 8.11 (dt, 1H, H-4), 7.45 (d, *J* = 8.2 Hz, 1H, CONH), 7.34 (dd, *J* = 7.1, 4.9 Hz, 1H, H-5), 7.08 (d, *J* = 7.4 Hz, 1H, CONH), 4.78–4.73 (m, 1H, H_Leu_-2), 4.53 (p, *J* = 7.2 Hz, 1H, H_Ala_-2), 3.74 (s, 3H, CO_2_CH_3_), 1.80–1.66 (m, 3H, H_Leu_-3 + H_Leu_-4), 1.38 (d, *J* = 7.2 Hz, 3H, H_Ala_-3), [0.94 (d, *J* = 6.2 Hz), 0.93 (d, *J* = 6.2 Hz), 6H, H_Leu_-5]. ^**13**^ **C­{** ^**1**^ **H} NMR and DEPT-135** (CDCl_3_, 101 MHz) δ ppm: [173.2 (C), 172.1 (C), 165.7 (C), 2 × CONH + CO_2_CH_3_], 152.4 (CH, C-2), 148.5 (CH, C-4), 135.4 (CH, C-6), 129.6 (C, C-3), 123.5 (CH, C-5), 52.6 (CH_3_, CO_2_CH_3_), 52.4 (CH, C_Leu_-2), 48.3 (CH, C_Ala_-2), 41.6 (CH_2_, C_Leu_-3), 25.0 (CH, C_Leu_-4), 23.0 (CH_3_, C_Leu_-5), 22.2 (CH_3_, C_Leu_-5), 18.1 (CH_3_, C_Ala_-3). **HRMS** (ESI-TOF) *m*/*z*: [M + H]^+^ Calcd for C_16_H_24_N_3_O_4_^+^: 322.17613; Found: 322.17609.

**Methyl nicotinoyl-** l-isoleucylglycinate (5e). Following the general protocol for peptide coupling, Nic (0.2581 g, 2.096 mmol) reacted with Et_3_N (0.87 mL, 6.3 mmol) and TBTU (0.7067 g, 2.201 mmol), followed by the addition of **4e** (0.6961 g, 2.201 mmol). After the workup, the resulting residue was purified by column chromatography using CH_2_Cl_2_/MeOH (20:1) as eluent, affording **5e** (0.5621 g) as a white solid. **Yield:** 87%. **m.p.**: 164–168 °C. **R**_***f***_: 0.43 in CH_2_Cl_2_/MeOH (20:1). ${[\alpha ]}_{D}^{23}$ : −3.75 ± 0.10 (*c*0.995, CHCl_3_). ^**1**^ **H NMR** (CDCl_3_, 400 MHz) δ ppm: 9.06 (br s, 1H, H-2), 8.72 (d, *J* = 3.6 Hz, 1H, H-6), 8.13 (dt, *J* = 8.0, 1.8 Hz, 1H, H-4), 7.37 (dd, *J* = 7.6, 4.9 Hz, 1H, H-5), 7.32 (d, *J* = 8.6 Hz, 1H, CONH), 7.02 (t, *J* = 4.9 Hz, 1H, CONH), 4.61 (dd, *J* = 8.6, 7.6 Hz, 1H, H_Ile_-2), [4.14 (dd, *J* = 18.1, 5.7 Hz, 1H), 3.97 (dd, *J* = 18.1, 5.1 Hz, 1H), H_Gly_-2], 3.73 (s, 3H, CO_2_CH_3_), 2.06–1.93 (m, 1H, H_Ile_-3), [1.62 (dtt, *J* = 14.9, 7.4, 3.7 Hz, 1H), 1.33–1.13 (m, 1H), H_Ile_-4], 1.00 (d, *J* = 6.8 Hz, 3H, H_Ile_-6), 0.92 (d, *J* = 7.4 Hz, 3H, H_Ile_-5). ^**13**^ **C­{** ^**1**^ **H} NMR and DEPT-135** (CDCl_3_, 101 MHz) δ ppm: [171.7 (C), 170.0 (C), 165.7 (C), 2 × CONH + CO_2_CH_3_], 152.2 (CH, C-2), 148.3 (CH, C-4), 135.6 (CH, C-6), 129.9 (C, C-3), 123.7 (CH, C-5), 58.3 (CH, C_Ile_-2), 52.5 (CH_3_, CO_2_CH_3_) 41.3 (CH_2_, C_Gly_-2), 37.5 (CH, C_Ile_-3), 25.3 (CH_2_, C_Ile_-4), 15.5 (CH_3_, C_Ile_-6), 11.3 (CH_3_, C_Ile_-5). **HRMS** (ESI-TOF) *m*/*z*: [M + H]^+^ Calcd for C_15_H_22_N_3_O_4_^+^: 308.16048; Found: 308.16049.

**Methyl nicotinoyl-** l-isoleucyl- **l** **-alaninate (5f).** Following the general protocol for peptide coupling, Nic (0.2111 g, 1.715 mmol) reacted with Et_3_N (0.72 mL, 5.2 mmol) and TBTU (0.5783 g, 1.801 mmol), followed by the addition of **4f** (0.5949 g, 1.801 mmol). After the workup, the resulting residue was purified by column chromatography using CH_2_Cl_2_/MeOH (10:1) as eluent, affording **5f** (0.3889 g) as a white solid. **Yield:** 71%. **m.p.**: 180–183 °C. **R**_***f***_: 0.58 in CH_2_Cl_2_/MeOH (10:1). ${[\alpha ]}_{D}^{21}$ : −0.79 ± 0.11 (*c*0.990, CHCl_3_). ^**1**^ **H NMR** (CDCl_3_, 400 MHz) δ ppm: 9.05 (br s, 1H, H-2), 8.70 (d, *J* = 3.7 Hz, 1H, H-6), 8.11 (d, *J* = 8.0 Hz, 1H, H-4), 7.45 (d, *J* = 8.6 Hz, 1H, CONH), 7.33 (dd, *J* = 8.0, 4.8 Hz, 1H, H-5), 7.15 (d, *J* = 7.2 Hz, 1H, CONH), 4.66–4.50 (m, 2H, H_Ile_-2 + H_Ala_-2), 3.73 (s, 3H, CO_2_CH_3_), 2.05–1.89 (m, 1H, H_Ile_-3), 1.69–1.56 (m, 1H, H_Ile_-4), 1.37 (d, *J* = 7.2 Hz, 3H, H_Ala_-3), 1.30–1.17 (m, 1H, H_Ile_-4), 1.00 (d, *J* = 6.7 Hz, 3H, H_Ile_-6), 0.90 (t, *J* = 7.4 Hz, 3H, H_Ile_-5). ^**13**^ **C­{** ^**1**^ **H} NMR and DEPT-135** (CDCl_3_, 101 MHz) δ ppm: [173.1 (C), 171.1 (C), 165.7 (C), 2 × CONH + CO_2_CH_3_], 152.5 (CH, C-2), 148.6 (CH, C-4), 135.3 (CH, C-6), 129.8 (C, C-3), 123.5 (CH, C-5), 58.2 (CH, C_Ile_-2), 52.5 (CH_3_, CO_2_CH_3_) 48.3 (CH, C_Ala_-2), 37.7 (CH, C_Ile_-3), 25.4 (CH_2_, C_Ile_-4), 18.0 (CH_3_, C_Ala_-3), 15.3 (CH_3_, C_Ile_-6), 11.3 (CH_3_, C_Ile_-5). **HRMS** (ESI-TOF) *m*/*z*: [M + H]^+^ Calcd for C_16_H_24_N_3_O_4_^+^: 322.17613; Found: 322.17612.

**Nicotinoyl-** **l** **-valylglycinamide (6a).** Following the general protocol for primary amide synthesis, methyl ester **5a** (0.2831 g, 0.9652 mmol) reacted with a 7 M NH_3_ solution in MeOH (25 mL) until completion (TLC). After the workup, **6a** (0.2686 g) was obtained as a white solid. Yield: quantitative. **m.p.**: 168–171 °C. **R**_***f***_: 0.00 in EtOAc. ${[\alpha ]}_{D}^{21}$ : +17.91 ± 0.10 (*c*1.015, MeOH). ^**1**^ **H NMR** (CDCl_3_, 400 MHz) δ ppm: 9.03 (dd, *J* = 2.3, 0.9 Hz, 1H, H-2), 8.71 (dd, *J* = 4.8, 1.7 Hz, 1H, H-6), 8.64 (d, *J* = 8.0 Hz, 1H, CONH), 8.32–8.26 (m, 1H, H-4), 8.21 (dt, *J* = 7.9, 2.0 Hz, 1H, H-5), 7.50 (ddd, *J* = 7.9, 4.8, 0.9 Hz, 1H, CONH), 7.21 (br s, 1H, CONH_2_), 7.06 (br s, 1H, CONH_2_), 4.27 (t, *J* = 7.9 Hz, 1H, H_Val_-2), [3.71 (dd, *J* = 16.7, 6.1 Hz, 1H), 3.61 (dd, *J* = 16.7, 5.6 Hz, 1H), H_Gly_-2], 2.15 (h, *J* = 6.8 Hz, 1H, H_Val_-3), [0.95 (d, *J* = 6.7 Hz), 0.94 (d, *J* = 6.7 Hz), 6H, H_Val_-4]. ^**13**^ **C­{** ^**1**^ **H} NMR and DEPT-135** (CDCl_3_, 101 MHz) δ ppm: [171.2 (C), 170.8 (C), 165.6 (C), 2 × CONH + CONH_2_], 151.9 (CH, C-2), 148.7 (CH, C-4), 135.4 (CH, C-6), 129.8 (C, C-3), 123.3 (CH, C-5), 59.5 (CH, C_Val_-2), 41.9 (CH_2_, C_Gly_-2), 29.7 (CH, C_Val_-3), [19.3 (CH), 18.9 (CH), C_Val_-4]. **HRMS** (ESI-TOF) *m*/*z*: [M + H]^+^ Calcd for C_13_H_19_N_4_O_3_^+^: 279.14517; Found: 279.14517.

**Nicotinoyl-** **l** **-valyl-** **l** **-alaninamide (6b).** Following the general protocol for primary amide synthesis, methyl ester **5b** (0.2906 g, 0.9455 mmol) reacted with a 7 M NH_3_ solution in MeOH (25 mL) until completion (TLC). After the workup, **6b** (0.2764 g) was obtained as a white solid. **Yield:** quantitative. **m.p.**: > 200 °C. **R**_***f***_: 0.00 in EtOAc. ${[\alpha ]}_{D}^{20}$ : −6.09 ± 0.09 (*c*1.020, MeOH). ^**1**^ **H NMR** (DMSO-*d*_6_, 400 MHz, rotamers) δ ppm: 9.01 (br s, 1H, H-2), [8.71 (d, *J* = 4.7 Hz), 8.71 (d, *J* = 4.8 Hz), 1H, H-6], [8.66 (d, *J* = 7.9 Hz), 8.55 (d, *J* = 8.5 Hz), 1H, CONH], [8.31 (d, *J* = 7.8 Hz), 8.25–8.16 (m, 1H), 8.03 (d, *J* = 7.4 Hz), H-4 + CONH], 7.56–7.46 (m, 1H, H-5), 7.26 (br s, 1H, CONH_2_), [7.05 (br s), 6.97 (br s), 1H, CONH_2_], 4.37–4.19 (m, 2H, H_Ala_-2 + H_Val_-2), 2.20–2.06 (m, 1H, H_Val_-3), 1.22 (d, *J* = 7.1 Hz, 3H, H_Ala_-3), 1.00–0.88 (m, 6H, H_Val_-4). ^**13**^ **C­{** ^**1**^ **H} NMR and DEPT-135** (DMSO-*d*_6_, 101 MHz, rotamers) δ ppm: [174.1 (C), 174.0 (C), 170.7 (C), 170.3 (C), 165.6 (C), 165.3 (C), 2 × CONH + CONH_2_], [151.9 (CH), 151.9 (CH), C-2], [148.7 (CH), 148.7 (CH), C-4], 135.3 (CH, C-6), [129.9 (C), 129.7 (C), C-3], 123.3 (CH, C-5), [59.7 (CH), 59.0 (CH), C_Ala_-2], [48.0 (CH), 47.9 (CH), C_Val_-2], [30.1 (CH), 29.7 (CH), C_Val_-3], [19.3 (CH_3_), 19.2 (CH_3_), 19.1 (CH_3_), 18.7 (CH_3_), C_Val_-4], [18.3 (CH_3_), 18.1 (CH_3_), C_Ala_-3]. **HRMS** (ESI-TOF) *m*/*z*: [M + H]^+^ Calcd for C_14_H_21_N_4_O_3_^+^: 293.16082; Found: 293.16086.

**Nicotinoyl-** **l** **-leucylglycinamide (6c).** Following the general protocol for primary amide synthesis, methyl ester **5c** (0.2112 g, 0.6872 mmol) reacted with a 7 M NH_3_ solution in MeOH (25 mL) until completion (TLC). After the workup, **6c** (0.2009 g) was obtained as a pale-yellow solid. **Yield:** quantitative. **m.p.**: 58–60 °C. **R**_***f***_: 0.00 in EtOAc. ${[\alpha ]}_{D}^{20}$ : −1.07 ± 0.11 (*c*1.070, MeOH). ^**1**^ **H NMR** (DMSO-*d*_6_, 400 MHz) δ ppm: 9.04 (dd, *J* = 2.3, 0.9 Hz, 1H, H-2), 8.78 (d, *J* = 7.6 Hz, 1H, H-6), 8.71 (dd, *J* = 4.8, 1.7 Hz, 1H, H-4), 8.26–8.21 (m, 2H, CONH + H-5), 7.51 (ddd, *J* = 7.9, 4.8, 0.9 Hz, CONH), 7.16 (br s, 1H, CONH_2_), 7.07 (br s, 1H, CONH_2_), 4.51–4.45 (m, 1H, H_Leu_-2), [3.67 (dd, *J* = 16.7, 6.0 Hz, 1H), 3.59 (dd, *J* = 16.7, 5.7 Hz, 1H), H_Gly_-2], 1.73–1.56 (m, 3H, H_Leu_-3 + H_Leu_-4), [0.92 (d, *J* = 6.1 Hz), 0.88 (d, *J* = 6.1 Hz), 6H, H_Leu_-5]. ^**13**^ **C­{** ^**1**^ **H} NMR and DEPT-135** (DMSO-*d*_6_, 101 MHz) δ ppm: [172.2 (C), 170.9 (C), 165.3 (C), 2 × CONH + CONH_2_], 151.9 (CH, C-2), 148.7 (CH, C-4), 135.3 (CH, C-6), 129.6 (C, C-3), 123.3 (CH, C-5), 52.1 (CH, C_Leu_-2), 42.0 (CH_2_, C_Gly_-2), 40.0 (CH_2_, C_Leu_-3), 24.4 (CH, C_Leu_-4), [23.0 (CH_3_), 21.4 (CH_3_), C_Leu_-5]. **HRMS** (ESI-TOF) *m*/*z*: [M + H]^+^ Calcd for C_14_H_21_N_4_O_3_^+^: 293.16082; Found: 293.16107.

**Nicotinoyl-** **l** **-leucyl-** **l** **-alaninamide (6d).** Following the general protocol for primary amide synthesis, methyl ester **5d** (0.1969 g, 0.6127 mmol) reacted with a 7 M NH_3_ solution in MeOH (25 mL) until completion (TLC). After the workup, **6d** (0.1877 g) was obtained as a white solid. **Yield:** quantitative. **m.p.**: 195–198 °C. **R**_***f***_: 0.00 in EtOAc. ${[\alpha ]}_{D}^{19}$ : −22.71 ± 0.05 (*c*1.050, MeOH). ^**1**^ **H NMR** (DMSO-*d*_6_, 400 MHz) δ ppm: 9.03 (dd, *J* = 2.3, 0.9 Hz, 1H, H-2), 8.72–8.70 (m, 2H, H-6 + CONH), 8.22 (dt, *J* = 7.9, 2.0 Hz, 1H, H-4), 7.99 (d, *J* = 7.6 Hz, 1H, CONH), 7.51 (ddd, *J* = 8.0, 4.9, 0.9 Hz, 1H, H-5), 7.24 (br s, 1H, CONH_2_), 6.99 (br s, 1H, CONH_2_), 4.51 (ddd, *J* = 10.3, 7.9, 4.5 Hz, 1H, H_Leu_-2), 4.21 (p, *J* = 7.1 Hz, 1H, H_Ala_-2), 1.73–1.64 (m, 2H, H_Leu_-3), 1.60–1.53 (m, 1H, H_Leu_-4), 1.22 (d, *J* = 7.1 Hz, 3H, H_Ala_-3), [0.91 (d, *J* = 6.3 Hz), 0.88 (d, *J* = 6.3 Hz), 6H, H_Leu_-5]. ^**13**^ **C­{** ^**1**^ **H} NMR and DEPT-135** (DMSO-*d*_6_, 101 MHz) δ ppm: [174.1 (C), 171.5 (C), 165.1 (C), 2 × CONH + CONH_2_], 151.9 (CH, C-2), 148.6 (CH, C-4), 135.2 (CH, C-6), 129.6 (C, C-2), 123.4 (CH, C-5), 51.9 (CH, C_Leu_-2), 48.0 (CH, C_Ala_-2), 40.2 (CH_2_, C_Leu_-3), 24.4 (CH, C_Leu_-4), [23.1 (CH_3_), 21.3 (CH_3_), C_Leu_-5], 18.3 (CH_3_, C_Ala_-3). **HRMS** (ESI-TOF) *m*/*z*: [M + H]^+^ Calcd for C_15_H_23_N_4_O_3_^+^: 307.17647; Found: 307.17648.

**Nicotinoyl-** l-isoleucylglycinamide (6e). Following the general protocol for primary amide synthesis, methyl ester **5e** (0.2301 g, 0.7487 mmol) reacted with a 7 M NH_3_ solution in MeOH (25 mL) until completion (TLC). After the workup, **6e** (0.2167 g) was obtained as a white solid. **Yield:** 99%. **m.p.**: 183–186 °C. **R**_***f***_: 0.00 in EtOAc. ${[\alpha ]}_{D}^{26}$ : +18.59 ± 0.10 (*c*0.990, MeOH). ^**1**^ **H NMR** (DMSO-*d*_6_, 400 MHz) δ ppm: 9.00 (br s, 1H, H-2), 8.77–8.59 (m, 2H, H-6 + CONH), 8.33 (br s, 1H, H-4), 8.20 (d, *J* = 7.5 Hz, 1H, CONH), 7.58–7.42 (m, 1H, H-5), 7.23 (br s, 1H, CONH_2_), 7.07 (br s, 1H, CONH_2_), 4.28 (t, *J* = 7.7 Hz, 1H, H_Ile_-2), 3.84–3.81 [3.71 (dd, *J* = 16.8, 5.9 Hz), H_Gly_-2 + H_2_O], 2.02–1.83 (m, 1H, H_Ile_-3), [1.59–1.46 (m, 1H), 1.27–1.13 (m, 1H), H_Ile_-4], 0.89 (d, *J* = 6.5 Hz, 3H, H_Ile_-6), 0.84 (t, *J* = 7.1 Hz, 3H, H_Ile_-5). ^**13**^ **C­{** ^**1**^ **H} NMR and DEPT-135** (DMSO-*d*_6_, 101 MHz) δ ppm: [171.6 (C), 171.3 (C), 165.9 (C), 2 × CONH + CONH_2_], 152.1 (CH, C-2), 148.8 (CH, C-4), 135.6 (CH, C-6), 129.9 (C, C-3), 123.6 (CH, C-5), 58.6 (CH, C_Ile_-2), 42.1 (CH_2_, C_Gly_-2), 35.8 (CH, C_Ile_-3), 25.1 (CH_2_, C_Ile_-4), 15.6 (CH_3_, C_Ile_-6), 11.0 (CH_3_, C_Ile_-5). **HRMS** (ESI-TOF) *m*/*z*: [M + H]^+^ Calcd for C_14_H_21_N_4_O_3_^+^: 293.16082; Found: 293.16092.

**Nicotinoyl-** l-isoleucyl- **l** **-alaninamide (6f).** Following the general protocol for primary amide synthesis, methyl ester **5f** (0.2556 g, 0.7953 mmol) reacted with NH_3_ 7 M in MeOH (25 mL) until completion (TLC). After the workup, **6f** (0.2437 g) was obtained as a white solid. **Yield:** quantitative. **m.p.**: > 200 °C. **R**_***f***_: 0.00 in EtOAc. ${[\alpha ]}_{D}^{20}$ : +87.84 ± 0.10 (*c*1.040, MeOH). ^**1**^ **H NMR** (DMSO-*d*_6_, 400 MHz) δ ppm: 9.01 (d, *J* = 1.7 Hz, 1H, H-2), 8.70 (dd, *J* = 4.8, 1.7 Hz, 1H, H-6), 8.59 (d, *J* = 8.4 Hz, 1H, CONH), 8.21 (dt, *J* = 7.9, 2.0 Hz, 1H, H-4), 8.04 (d, *J* = 7.5 Hz, 1H, CONH), 7.50 (ddd, *J* = 7.9, 4.8, 0.9 Hz, 1H, H-5), 7.25 (br s, 1H, CONH_2_), 6.98 (br s, 1H, CONH_2_), 4.35 (t, *J* = 8.3 Hz, 1H, H_Ile_-2), 4.24 (p, *J* = 7.1 Hz, 1H, H_Ala_-2), 1.99–1.85 (m, 1H, H_Ile_-3), 1.50 (dtd, *J* = 14.8, 7.4, 3.5 Hz, 1H, H_Ile_-4), 1.28–1.13 [1.22 (d, *J* = 7.0 Hz), 4H, H_Ile_-4 + H_Ala_-3], 0.91 (d, *J* = 6.8 Hz, 3H, H_Ile_-6), 0.84 (t, *J* = 7.4 Hz, 3H, H_Ile_-5). ^**13**^ **C­{** ^**1**^ **H} NMR and DEPT-135** (DMSO-*d*_6_, 101 MHz) δ ppm: [174.0 (C), 170.4 (C), 165.2 (C), 2 × CONH + CONH_2_], 151.9 (CH, C-2), 148.7 (CH, C-4), 135.3 (CH, C-6), 129.8 (C, C-3), 123.3 (CH, C-5), 57.9 (CH, C_Ile_-2), 48.0 (CH, C_Ala_-2), 36.0 (CH, C_Ile_-3), 24.8 (CH_2_, C_Ile_-4), 18.3 (CH_3_, C_Ala_-3), 15.4 (CH_3_, C_Ile_-6), 11.8 (CH_3_, C_Ile_-5). **HRMS** (ESI-TOF) *m*/*z*: [M + H]^+^ Calcd for C_15_H_23_N_4_O_3_^+^: 307.17647; Found: 307.17664.

### Pharmacology

#### Dopamine D_2_ Functional Assays

The assays were performed on human D_2_R expressed in CHO cells using a Cisbio cAMP kit to measure cAMP mobilization via HTRF, as previously described.^18–20,66^ A total of 5,000 cells/well were seeded on a 96-well black plate in stimB buffer (provided in the kit) in the presence of 500 μM IBMX (a pan-phosphodiesterase inhibitor), to ensure maximal cAMP accumulation. Test compounds and DA were then added and incubated for 10 min at 37 °C, followed by a 5 min incubation with 10 μM forskolin. Subsequently, the kit reagents were added and incubated for 1 h at room temperature, after which the HTRF signal was measured using a Tecan M1000 Pro multilabel reader. Compounds were evaluated at 0.01 nM and 1 nM over a DA concentration–response curve. Data were converted to cAMP concentrations using a standard cAMP curve, and a DA curve was included as a positive control in all the assays. The activity of the compounds was expressed as the percentage of the response elicited by 0.1 μM DA.

### Biological Assays

#### Calcein-AM Experiment

The assay was performed as previously described by Riganti et al. with minor modifications.^38^ MDCK-MDR1 cells (kindly provided by Prof. P. Borst, NKI-AVL Institute, Amsterdam, The Netherlands) were cultured in Dulbecco’s modified Eagle medium (DMEM) supplemented with 10% fetal bovine serum (FBS), 2 mM glutamine, 100 U/mL penicillin, and 0.1 mg/mL streptomycin (Euroclone, Milan, Italy), under humidified conditions at 37 °C and 5% CO_2_ using a Form Direct Heat CO_2_ Incubator (Thermo Fisher Scientific, Waltham, MA, USA). Cells were seeded in black 96-well CulturePlates at a density of 30,000 cells per well in 100 μL of medium and allowed to adhere overnight. On the next day, the compounds under study (**5b**, **5c**, **6c**, **6f**, and MIF-1) were added to the monolayers in new complete DMEM, with final concentrations ranging from 0.1 to 100 μM, followed by incubation at 37 °C for 30 min. Subsequently, calcein-AM was added in PBS (100 μL) to a final concentration of 2.5 μM, and incubation was continued for 30 min. After incubation, the cells were washed three times with ice-cold PBS, and saline buffer was added (100 μL per well) prior to fluorescence measurement using a Victor 3 microplate reader (PerkinElmer) at excitation and emission wavelengths of 485 and 535 nm, respectively. Calcein accumulation was quantified in the absence and presence of test compounds, using untreated cells to define basal fluorescence levels. Compound activity was expressed as percentage inhibition at 100 μM.

### Cytotoxicity in HepG2 Cells

HepG2 cells purchased from the American Type Culture Collection (ATCC, Manassas, VA, USA) were used for hepatotoxicity evaluation. Cells were cultured in Eagle’s Minimum Essential Medium (MEM; Euroclone S.p.A., Pero, MI, Italy), supplemented with 10% FBS, 1% l-glutamine, 100 U/mL penicillin/streptomycin, and 1% nonessential amino acids (NEAA) (all from Euroclone S.p.A., Pero, MI, Italy). Cultures were maintained at 37 °C in a humidified atmosphere containing 5% CO_2_ using a Form Direct Heat CO_2_ Incubator (Thermo Fisher Scientific, Waltham, MA, USA) throughout the experimental procedures. For the experiments, cells were washed with calcium- and magnesium-free PBS (Corning, Milan, Italy), detached using trypsin-EDTA solution (Corning, Milan, Italy), and counted using a Countess 3 FL Automated Cell Counter (Thermo Fisher Scientific, Waltham, MA, USA). HepG2 cells were seeded in 96-well plates at a density of 10,000 cells per well and allowed to adhere overnight. On the next day, the compounds under study (**5b**, **5c**, **6c**, **6f**, and MIF-1) were added at a final concentration of 100 μM in new complete MEM. Vehicle controls containing equivalent concentrations of DMSO were included in all experiments to evaluate any potential solvent-related cytotoxicity. Cytotoxicity was assessed after 72 h of exposure using the MTT reduction colorimetric assay, as previously described.^67^ The MTT reagent was purchased from Sigma-Aldrich (Merck KGaA, Darmstadt, Germany).

### Cytotoxicity in the Human Differentiated SH-SY5Y Cells

Human neuroblastoma SH-SY5Y cells were used for neurotoxicity evaluation (Sigma-Aldrich, Taufkirchen, Germany). Cells were cultured in DMEM supplemented with GlutaMAX Gibco and 10% (v/v) FBS Gibco-Invitrogen (Alfagene, Carcavelos, Portugal), including 1% (v/v) of penicillin-streptomycin: 100 U/mL penicillin and 100 μg/mL streptomycin (Biotecnómica, Porto, Portugal). Cultures were maintained at 37 °C in a humidified atmosphere containing 5% CO_2_ (Heraeus, Hanau, Germany) throughout all the procedures. For experiments, cells were washed with calcium- and magnesium-free PBS (Biochrom, Berlin, Germany), detached using trypsin-EDTA solution (Sigma-Aldrich, Berlin, Germany), and counted using 0.4% (w/v) trypan blue solution (Sigma-Aldrich, Berlin, Germany). Cells were seeded in multiwell plates at a density of 25,000 cells/cm^2^ in complete DMEM supplemented with 10 μM all-*trans* RA (Sigma-Aldrich, Berlin, Germany) and cultured for 3 days. On day 3, differentiation continued by exposure to 80 nM TPA (Sigma-Aldrich, Berlin, Germany) in complete DMEM for an additional 3 days, to yield a dopaminergic-like phenotype. On day 6, dopaminergic differentiated cells were treated with the compounds under study (**5b**, **5c**, **6c**, **6f**, and MIF-1, all at 100 μM) or 6-OHDA (125 μM) for 48 h in new complete DMEM. In this assay, 6-OHDA, a well-established dopaminergic neurotoxin, was used as a positive control for cytotoxicity.^45,50^ All tested compounds were dissolved in sterile DMSO (Merck, Berlin, Germany) at a final concentration of 0.5% v/v in the wells, except 6-OHDA, which was freshly prepared in sterile PBS immediately before use. After 48 h of incubation, cytotoxicity was assessed using the MTT reduction and NR uptake assays.

### Neuroprotection in the Human Differentiated SH-SY5Y Cells

For the neuroprotective evaluation, following the 6-day differentiation period, a coincubation protocol was employed. Dopaminergic differentiated SH-SY5Y cells were pretreated with MIF-1, Nic, MIF-1 + Nic, **6c**, or **6f** (100 μM) for 60 min, followed by exposure to the neurotoxicant 6-OHDA (25–100 μM) for 48 h. Cell morphology was assessed and analyzed at the end of the incubation period using a Leica DMI 6000B microscope equipped with a Leica DFC350 FX camera and Leica LAS X software (v3.7.4; Leica Microsystems, Wetzlar, Germany). Neuroprotection was quantified using the MTT reduction assay.

### MTT Reduction Assay

The tetrazolium salt MTT is reduced to the corresponding formazan by cellular dehydrogenases, mainly mitochondrial.^45,50,67^ After the incubation period of each drug, MTT solution was added to each well at a final concentration of 0.5 mg/mL. Cells were then incubated at 37 °C and 5% CO_2_ for 3 h (HepG2 cells) or 1.5 h (SH-SY5Y cells). The reaction was stopped by removing the medium, followed by the addition of DMSO to solubilize the formazan crystals. Plates were then shaken for 15 min, protected from light. Absorbance was measured at 570 nm using the multiwell plate readers Victor 3 (PerkinElmer, Shelton, CT, USA), Synergy HT (BioTek Instruments, Winooski, VT, USA), or Infinite M200 (Tecan Life Sciences, Männedorf, Switzerland), with background correction. Results were expressed as a percentage of control cells (set at 100%) after subtraction of the reference absorbance for each well.

### NR Uptake Assay

The NR dye permeates viable cells and accumulates in lysosomes via active transport, serving as an indicator of lysosomal integrity.^49^ After the 48 h incubation, the culture medium was replaced with 250 μL/well of NR solution (33 μg/mL in DMEM), followed by incubation for 1.5 h at 37 °C, protected from light. The NR solution was then removed, and cells were washed with 250 μL/well of prewarmed Hank’s balanced salt solution (HBSS). Subsequently, NR dye was extracted from viable cells using 250 μL/well of extraction solution (50% ethanol/1% acetic acid), followed by 15 min of gentle shaking, in the dark. Absorbance was measured at 540 and 690 nm,^49^ in a multiwell plate reader (Biotech Synergy HT, Winooski, VT, USA). Results were expressed as a percentage of control cells, set to 100%, after subtracting the absorbance reference value of each well. All conditions were performed in quadruplicate in each independent experiment.

### Statistical Analysis

Results are presented as mean ± standard deviation. Statistical analysis was performed using one-way analysis of variance (ANOVA), followed by Tukey’s *post hoc* test when a statistical significance was achieved (*p* < 0.05). All analyses were conducted using GraphPad Prism 8.3 (GraphPad Software, San Diego, CA, USA).

## Supplementary Material







## Notes

NMR spectra for compounds **4a**, **4­(d–f)**, and **5–6­(a–f)**; HRMS spectra for compounds **5–6­(a–f)**; RP-HPLC chromatogram for **6c**; data from functional assays at the *h*D_2_R for compounds **5–6­(a–f)** and MIF-1; table of Cartesian coordinates for **6c**; physicochemical properties analysis and drug-likeness assessment (PDF)
FAIR NMR data, including the primary ^1^H, ^13^C­{^1^H}, and DEPT-135 NMR FID files for compounds **4a**, **4­(d–f)**, and **5–6­(a–f)** (ZIP)
Molecular formula strings of all compounds in the data set in the .csv format (CSV)

## ¶

RISE-Health, Faculty of Medicine, University of Porto, 4200-319 Porto, Portugal

## Abbreviations Used

^1^H NMR: proton nuclear magnetic resonance
^13^C­{^1^H} NMR: proton-decoupled carbon-13 nuclear magnetic resonance
6-OHDA: 6-hydroxydopamine
Ala: l-alanine
BBB: blood–brain barrier
Boc: *N*-*tert*-butoxycarbonyl
cAMP: cyclic adenosine monophosphate
CEMUP: Centro de Materiais da Universidade do Porto
CHO: Chinese hamster ovary cells
CNS: central nervous system
DA: dopamine
DEPT-135: distortionless enhancement by polarization transfer-135
DMEM: Dulbecco’s modified Eagle medium
DMSO: dimethyl sulfoxide
DMSO-*d*_6_: hexadeuterated dimethyl sulfoxide
DR: dopamine receptors
EC_50_: half maximal effective concentration
EDTA: ethylenediaminetetraacetic acid
*E*_max_: maximum effect
ESI-TOF: electrospray ionization time-of-flight
Et_3_N: triethylamine
FBS: fetal bovine serum
Gly: glycine
HBSS: Hank’s balanced salt solution
HepG2: human liver cancer cell line
HPLC-DAD: high-performance liquid chromatography with diode array detection
HRMS: high-resolution mass spectrometry
HTRF: homogeneous time-resolved fluorescence
IEFPCM: integral equation formalism polarizable continuum model
Ile: l-isoleucine
Leu: l-leucine
MDCK-MDR1: Madin-Darby Canine Kidney cell line transfected with the human MDR1 gene
MEM: Eagle’s minimum essential medium
MeOH: methanol
MIF-1: melanocyte-stimulating hormone release-inhibiting factor 1
m.p.: melting point
MTT: 3-(4,5-dimethylthiazol-2-yl)-2,5-diphenyl tetrazolium bromide
Nic: nicotinic acid
NMR: nuclear magnetic resonance
NR: neutral red
P-gp: P-glycoprotein
PAINS: pan-assay interference compounds
PAM: positive allosteric modulator
PBS: phosphate-buffered saline
PD: Parkinson’s disease
Pic: picolinic acid
Pro: l-proline
RA: retinoic acid
R_*f*_: retention factor
ROS: reactive oxygen species
SH-SY5Y: human neuroblastoma cell line
TBTU: *O*-(benzotriazol-1-yl)-*N*,*N*,*N*′,*N*′-tetramethyluronium tetrafluoroborate
TLC: thin-layer chromatography
TPA: 12-*O*-tetradecanoylphorbol-13-acetate
Val: l-valine
