# Supplementary material for: Dual-Acting Vitamin B3‑Melanostatin Neuropeptide Hybrids as Potent Modulators of the Dopamine D2 Receptors with Neuroprotective Activity
Source: J Med Chem. 2026 Jun 18;69(13):15597–613. doi: 10.1021/acs.jmedchem.6c00697 (PMC13370884; doi:10.1021/acs.jmedchem.6c00697)
Supplement: Supplementary file 1 [file jm6c00697_si_001.pdf]

Supporting Information for:

Dual-Acting Vitamin B_3-Melanostatin Neuropeptide Hybrids as Potent Modulators of the Dopamine D_2 Receptors with Neuroprotective Activity

Beatriz L. Pires-Lima,[a,b] Sara C. Silva-Reis,[a,c] Xavier Cruz Correia,[a] Hugo F. Costa-Almeida,[a] Vera M. Costa,[c,d,#] Xerardo García-Mera,[e] José Brea,[f] María I. Loza,[f] Marialessandra Contino,[g] Maria Grazia Perrone,[g] Giovanni Graziano,[g] Nuno Vale,[b,h,i] José E. Rodríguez-Borges,[a] and Ivo E. Sampaio-Dias[a,*]

[a]*LAQV/REQUIMTE, Department of Chemistry and Biochemistry, Faculty of Sciences, University of Porto, 4169-007 Porto, Portugal.*

[b]*PerMed Research Group, RISE-Health, Faculty of Medicine, University of Porto, 4200-319 Porto, Portugal.*

[c]*UCIBIO, REQUIMTE, Laboratory of Toxicology, Faculty of Pharmacy, University of Porto, 4050-313, Porto, Portugal.*

[d]*Associate Laboratory i4HB – Institute for Health and Bioeconomy, Faculty of Pharmacy, University of Porto, 4050-313 Porto, Portugal.*

[e]*Department of Organic Chemistry, Faculty of Pharmacy, University of Santiago de Compostela, E-15782 Santiago de Compostela, Spain.*

[f]*Innopharma Screening Platform. Biofarma Research Group. Centre of Research in Molecular Medicine and Chronic Diseases (CIMUS), University of Santiago de Compostela, E-15782 Santiago de Compostela, Spain.*

[g]*Department of Pharmacy - Pharmaceutical Sciences, University of Bari "Aldo Moro", Via E. Orabona 4, 70125 Bari, Italy.*

[h]*RISE-Health, Department of Community Medicine, Health Information and Decision (MEDCIDS), Faculty of Medicine, University of Porto, 4200-450 Porto, Portugal.*

[i]*Laboratory of Personalized Medicine, Department of Community Medicine, Health Information and Decision (MEDCIDS), Faculty of Medicine, University of Porto, 4200-450 Porto, Portugal.*

[#]*Current address: RISE-Health, Faculty of Medicine, University of Porto, 4200-319 Porto, Portugal.*

***Corresponding author e-mail**:
ivdias@fc.up.pt (Ivo E. Sampaio-Dias)

Table of Contents

1. NMR Spectra for Compounds **4a**, **4(d-f)**, and **5-6(a-f)** SI-2
2. HRMS Spectra for Compounds **5-6(a-f)** SI-18
3. RP-HPLC Chromatogram for **6c** SI-24
4. Data from Functional Assays at the *h*D_2R for Compounds **5-6(a-f)** and MIF-1 SI-25
5. Data from Neurotoxicity Evaluation at Dopaminergic Differentiated SH-SY5Y Cells for Compound **I** and MIF-1 SI-26
6. Table of Cartesian Coordinates for **6c** SI-27
7. Physicochemical Properties Analysis and Drug-Likeness Assessment SI-28
8. References SI-30

1. NMR Spectra for Compounds 4a, 4(d-f), and 5-6(a-f)

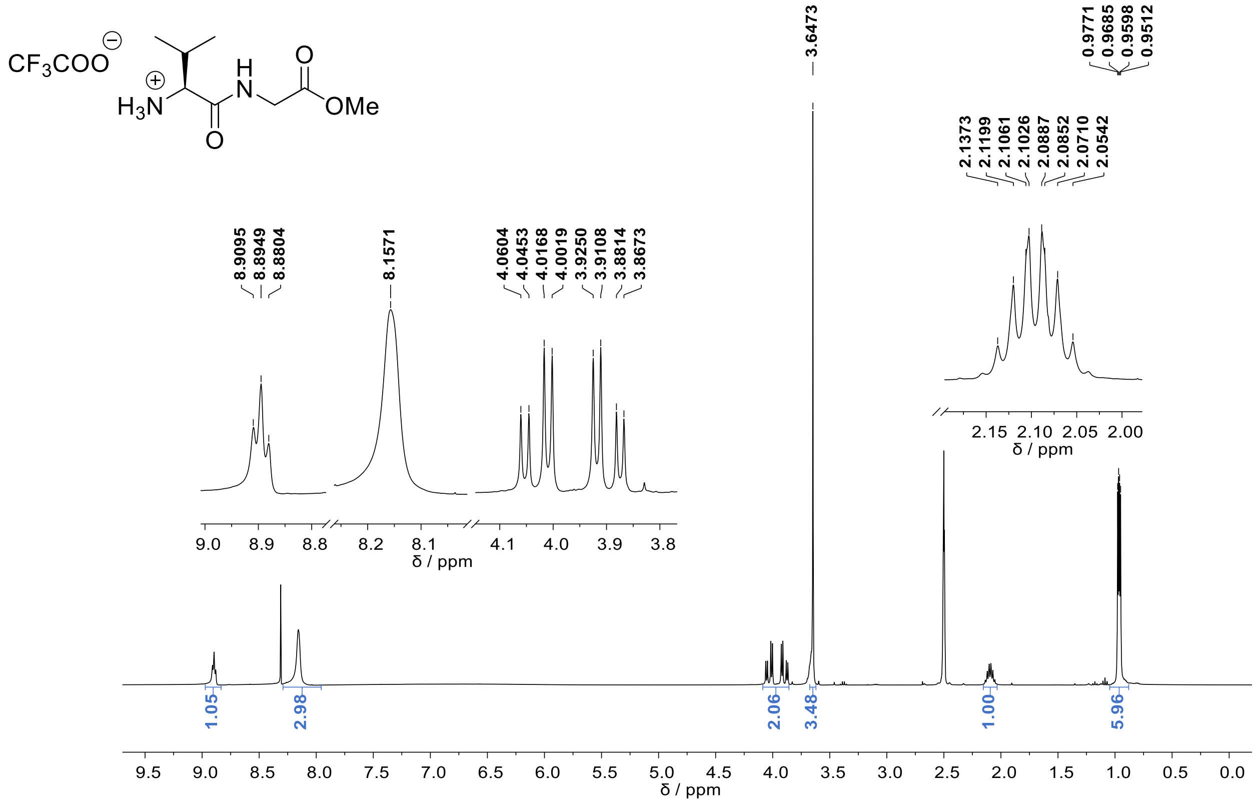

Figure S1. ^{1}H NMR spectrum (DMSO-d_6, 400 MHz) of dipeptide **4a**.

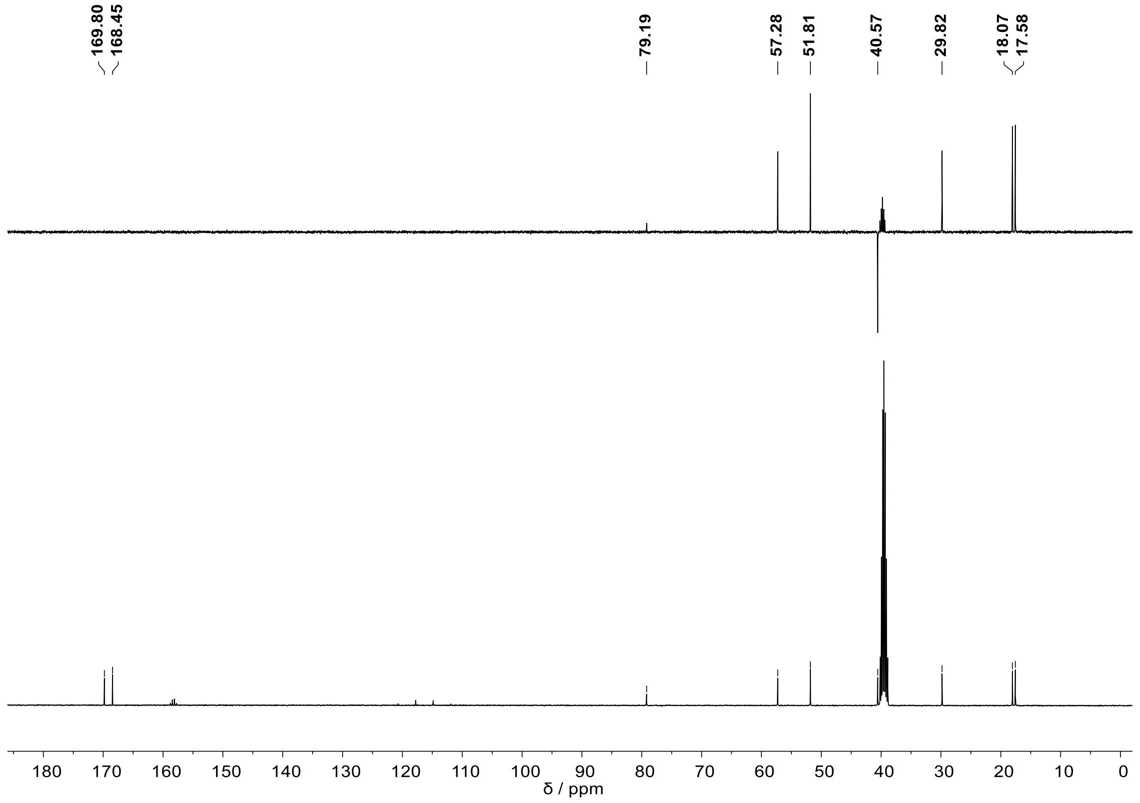

Figure S2. $^{13}C\{^{1}H\}$ and DEPT-135 NMR spectra (DMSO-d_6, 101 MHz) of dipeptide **4a**.

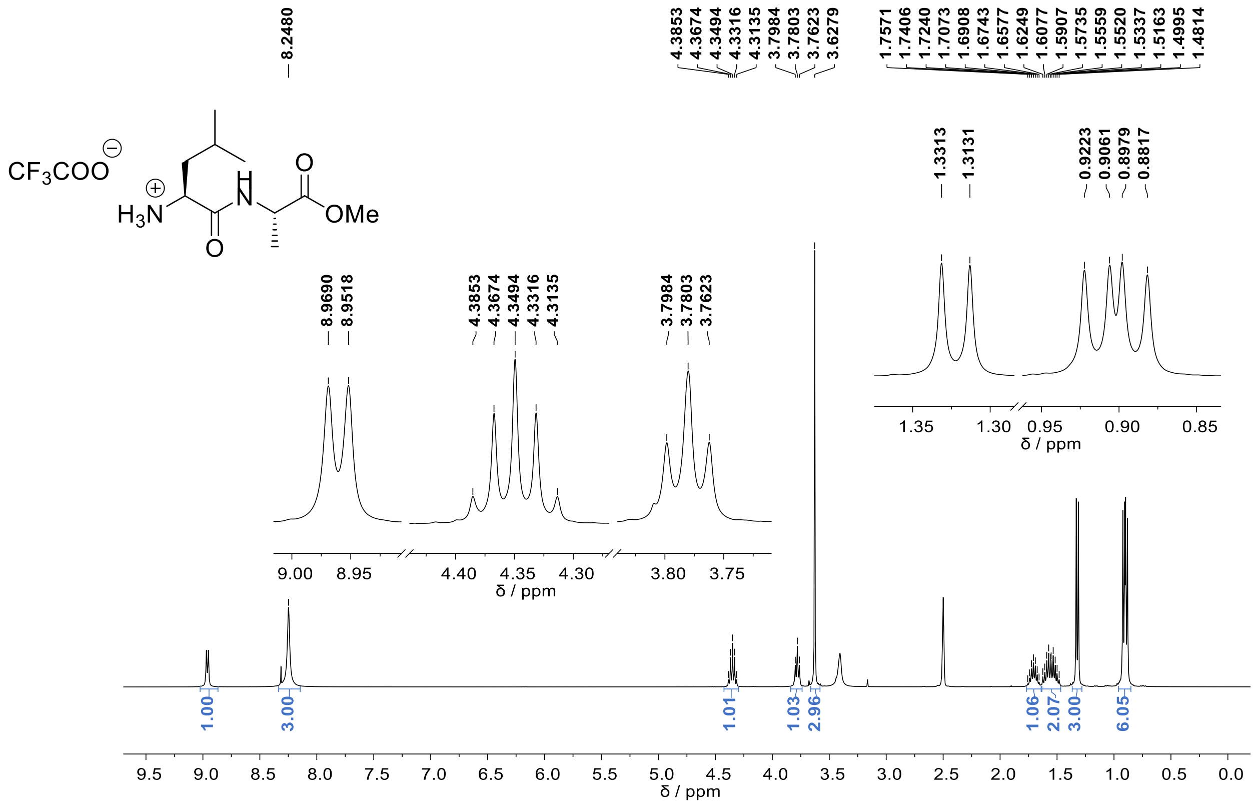

Figure S3. ^{1}H NMR spectrum (DMSO-d_6, 400 MHz) of dipeptide **4d**.

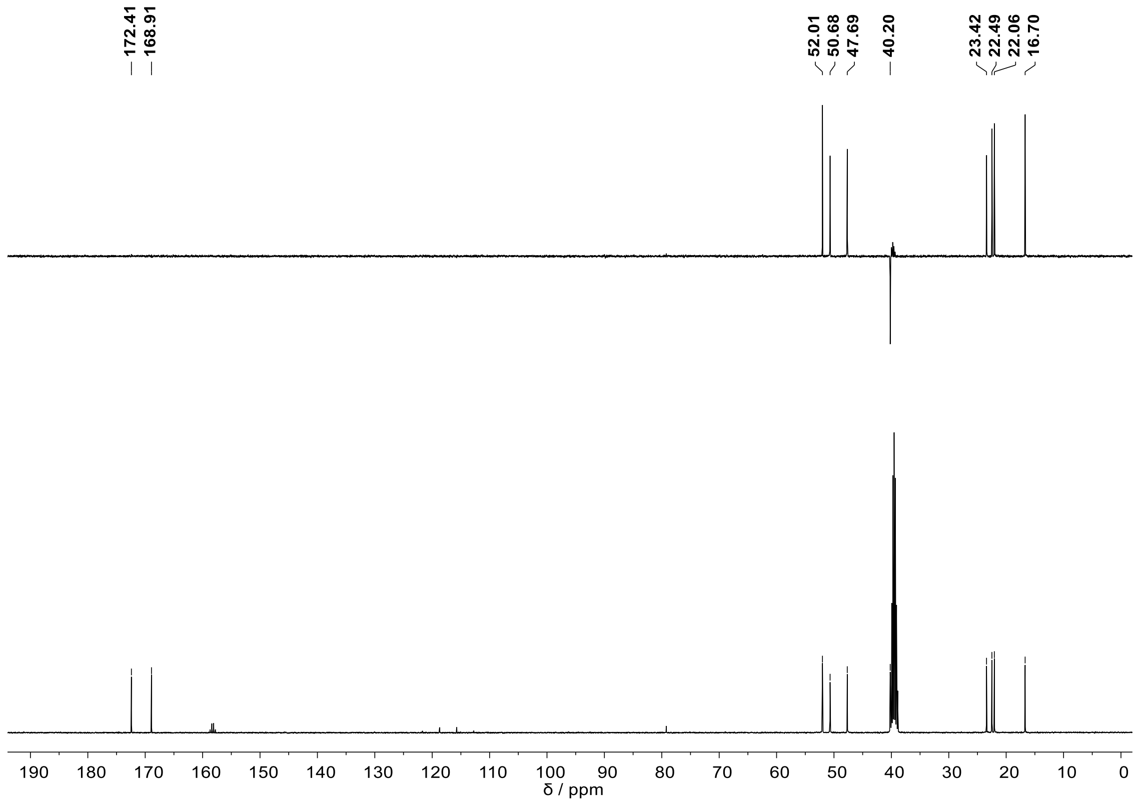

Figure S4. ^{13}C{^{1}H} and DEPT-135 NMR spectra (DMSO-d_6, 101 MHz) of dipeptide **4d**.

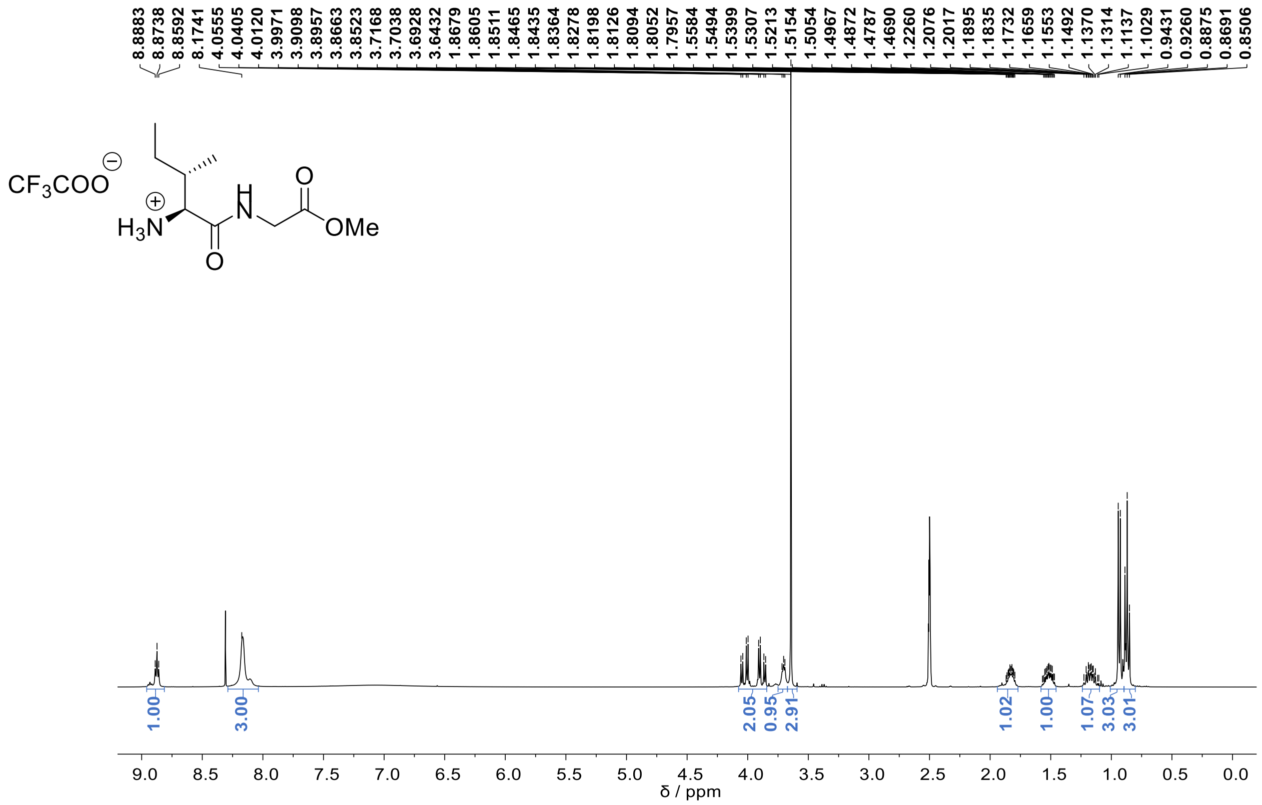

Figure S5. ^{1}H NMR spectrum (DMSO-d_6, 400 MHz) of dipeptide **4e**.

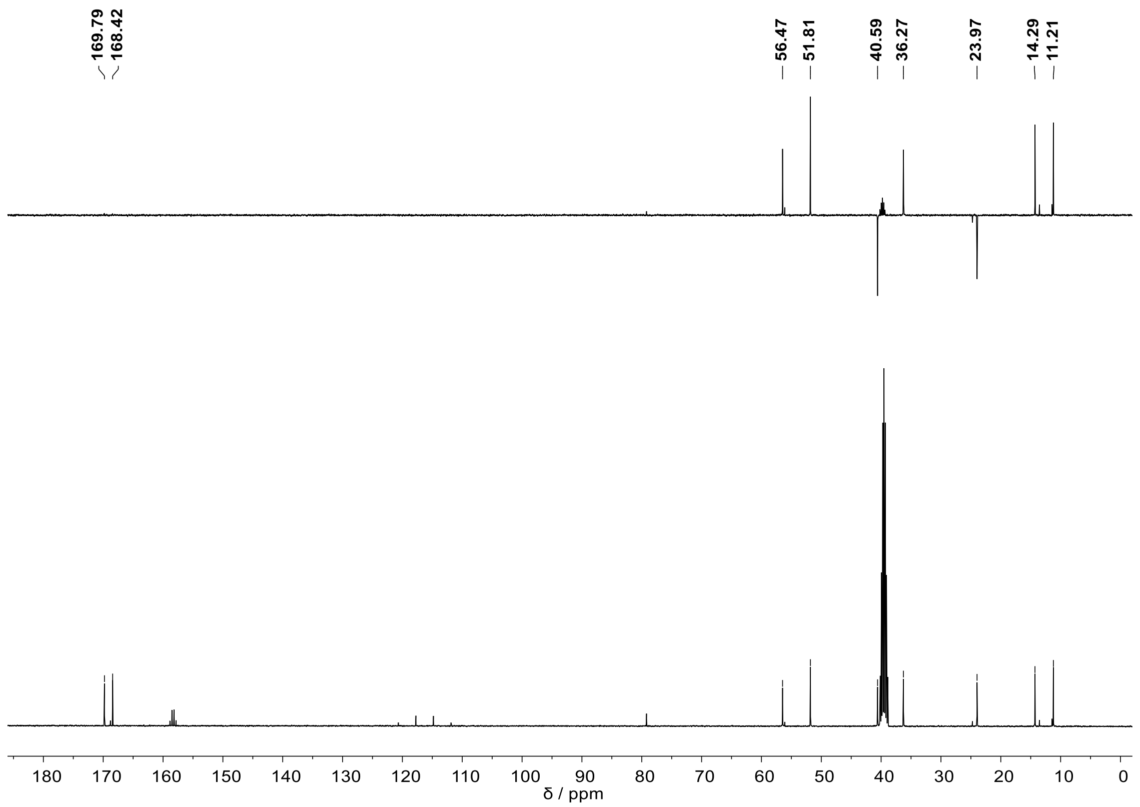

Figure S6. ^{13}C{^{1}H} and DEPT-135 NMR spectra (DMSO-d_6, 101 MHz) of dipeptide **4e**.

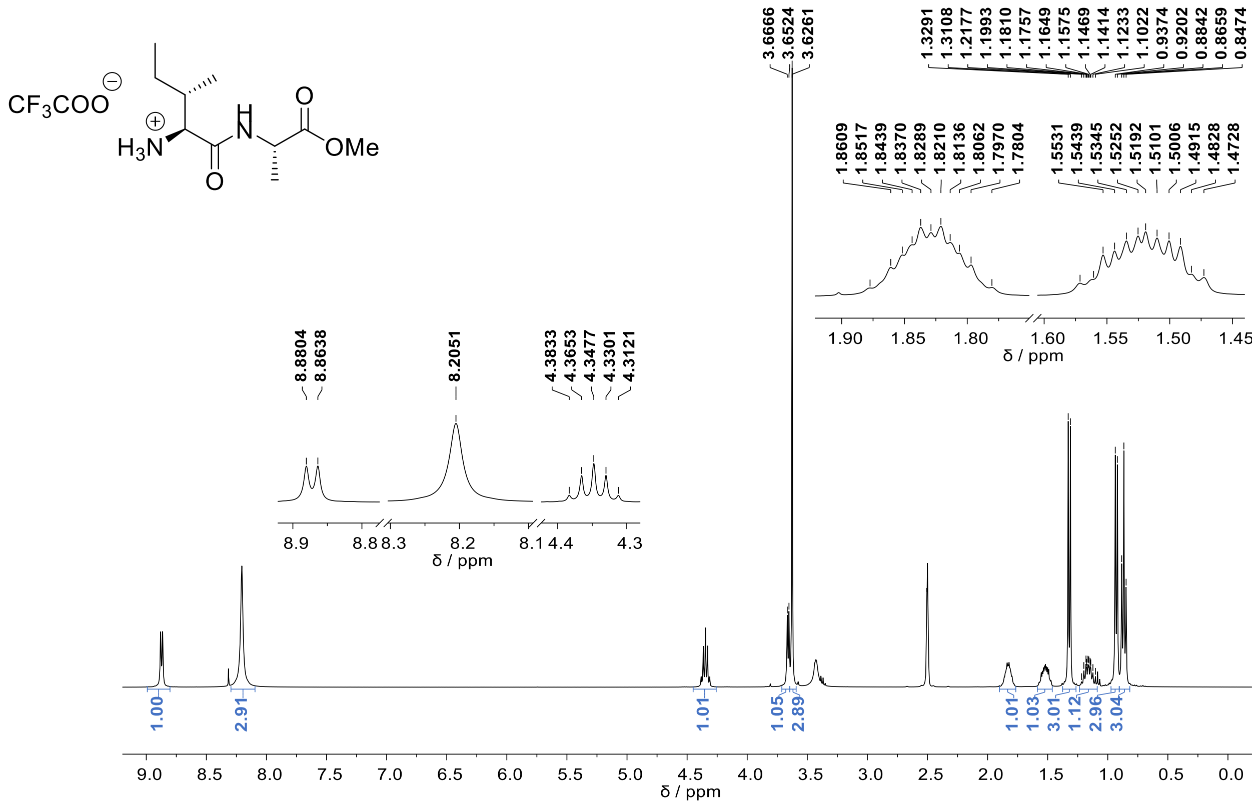

Figure S7. ^{1}H NMR spectrum (DMSO-d_6, 400 MHz) of dipeptide **4f**.

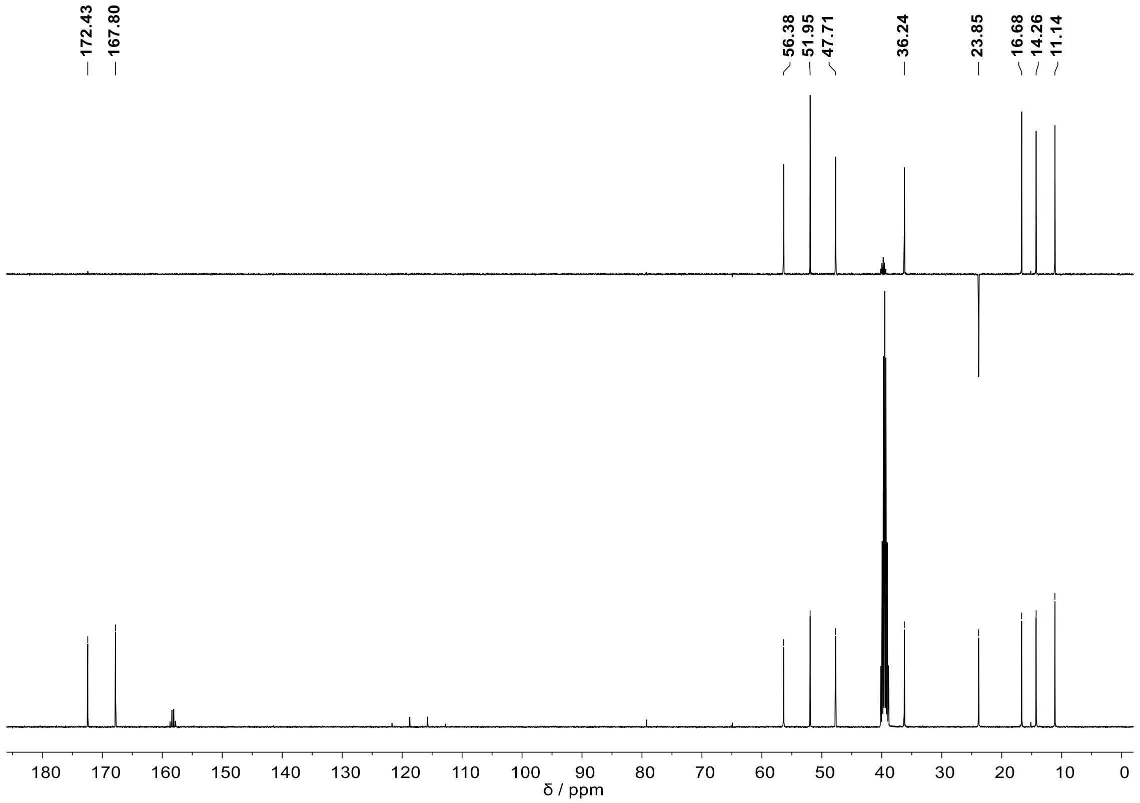

Figure S8. ^{13}C{^{1}H} and DEPT-135 NMR spectra (DMSO-d_6, 101 MHz) of dipeptide **4f**.

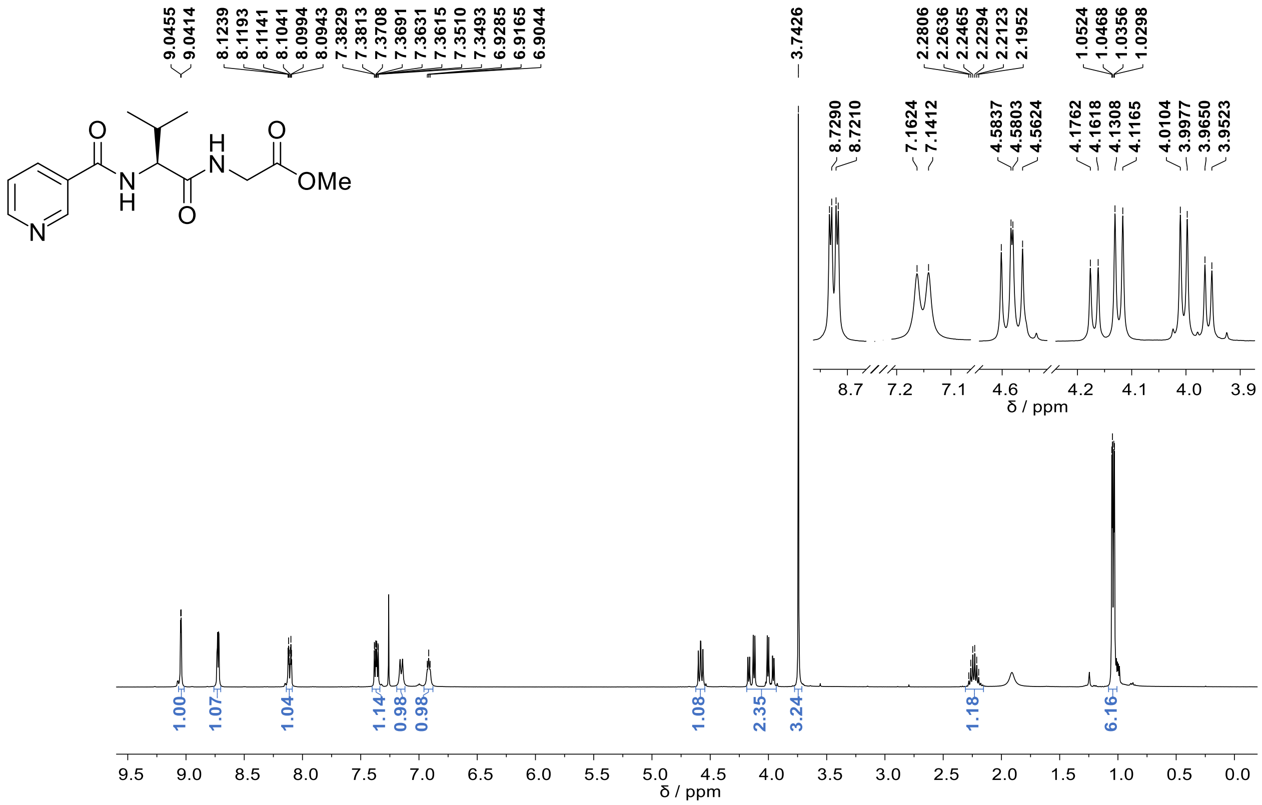

Figure S9. ^{1}H NMR spectrum ($CDCl_3$, 400 MHz) of compound **5a**.

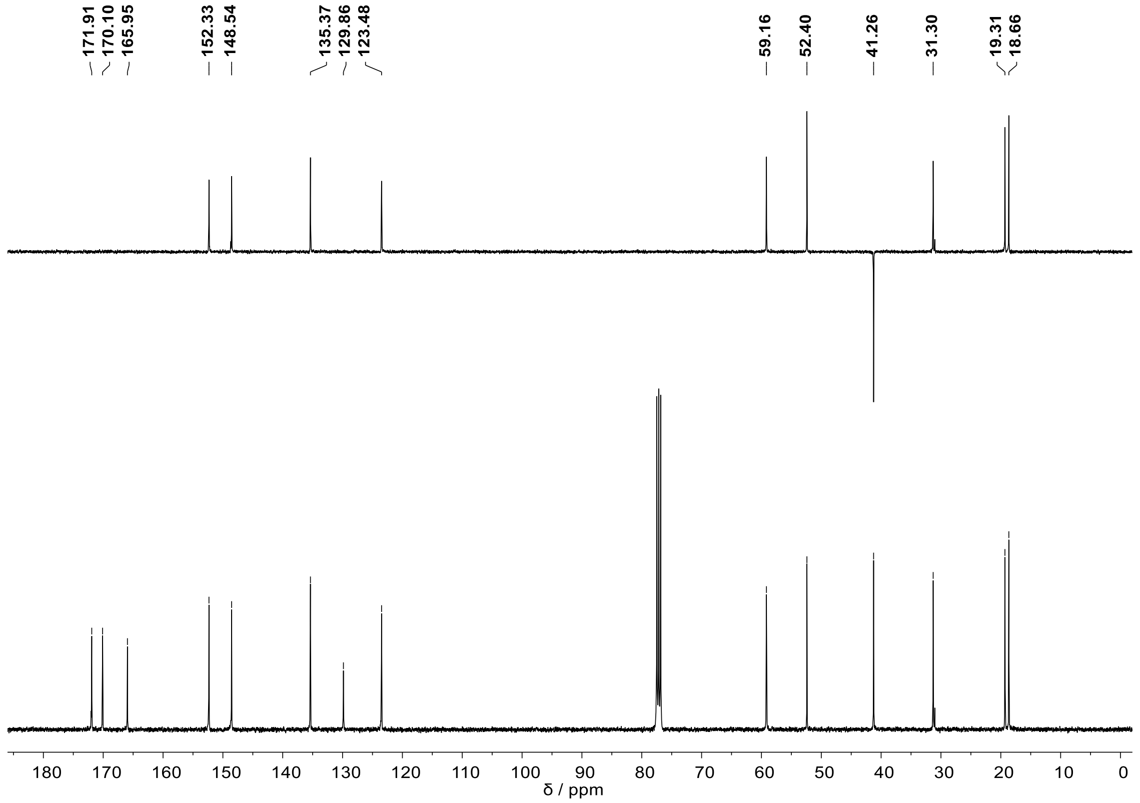

Figure S10. ^{13}C{^{1}H} and DEPT-135 NMR spectra ($CDCl_3$, 101 MHz) of compound **5a**.

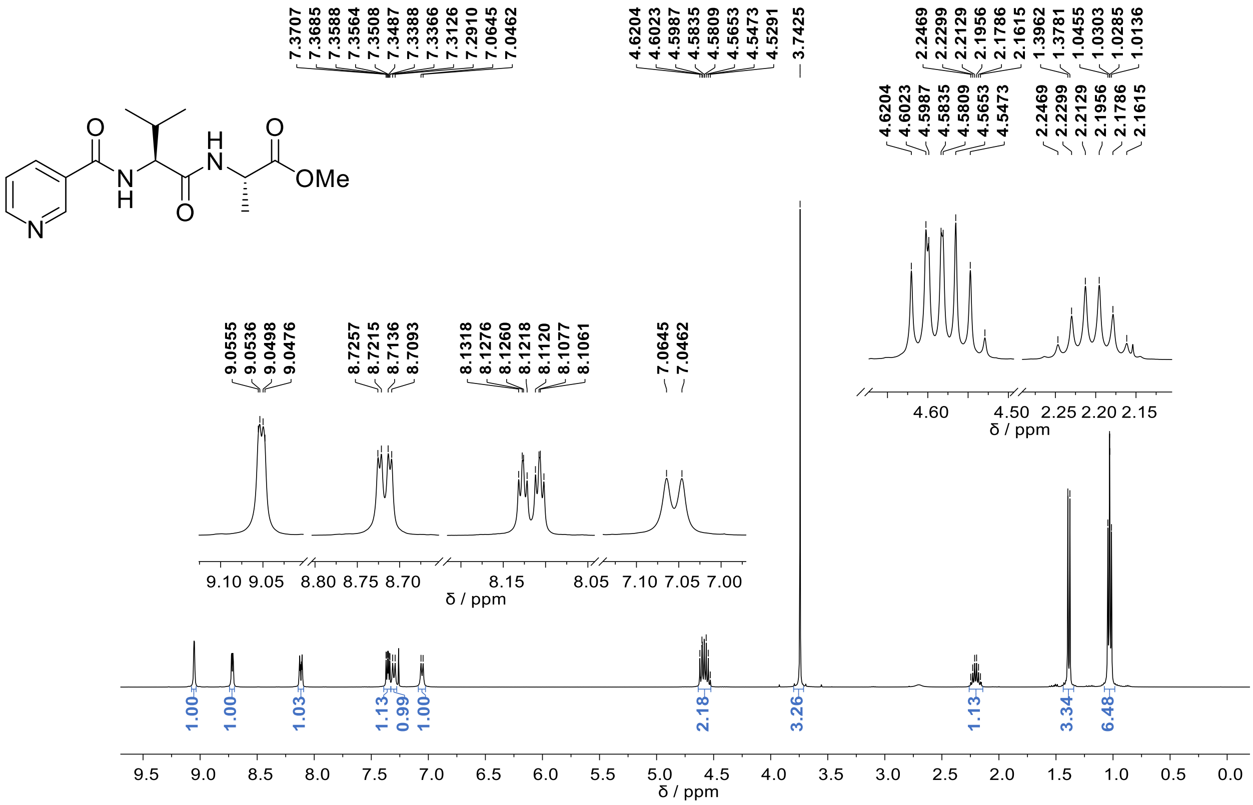

Figure S11. ^{1}H NMR spectrum ($CDCl_3$, 400 MHz) of compound **5b**.

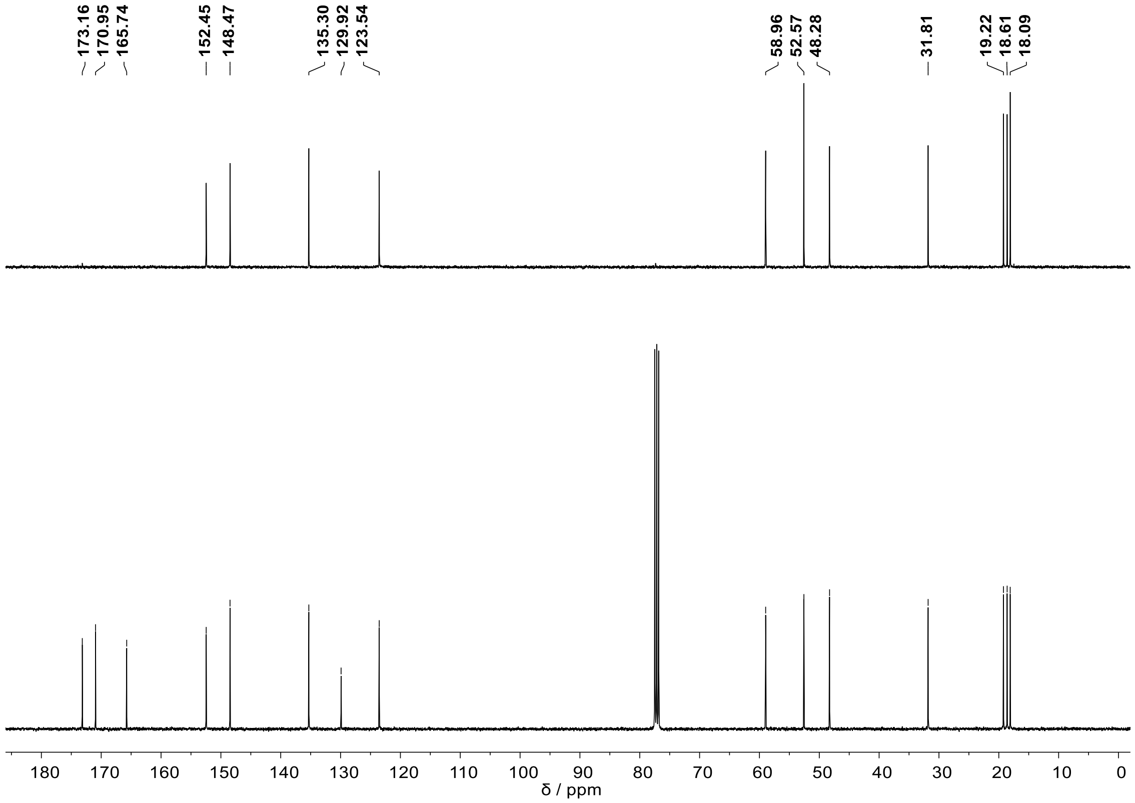

Figure S12. $^{13}C\{^{1}H\}$ and DEPT-135 NMR spectra ($CDCl_3$, 101 MHz) of compound **5b**.

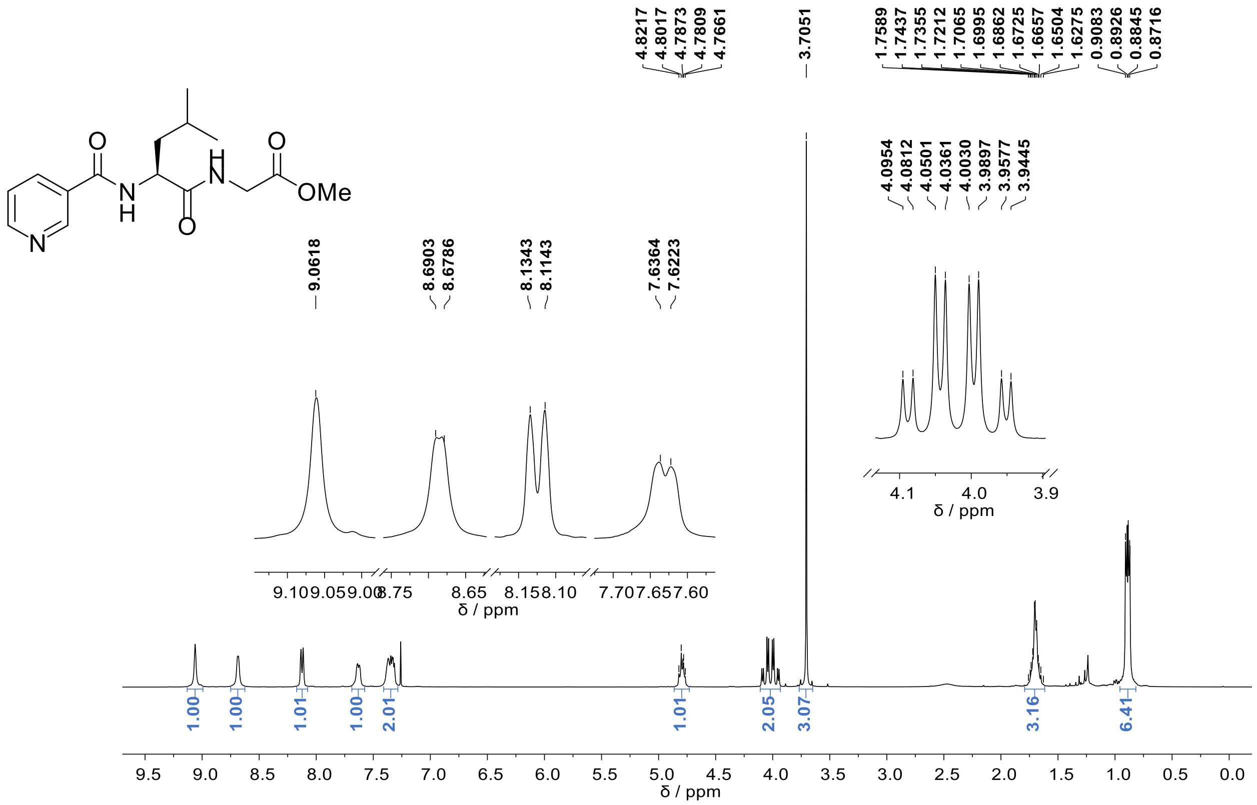

Figure S13. ^{1}H NMR spectrum ($CDCl_3$, 400 MHz) of compound **5c**.

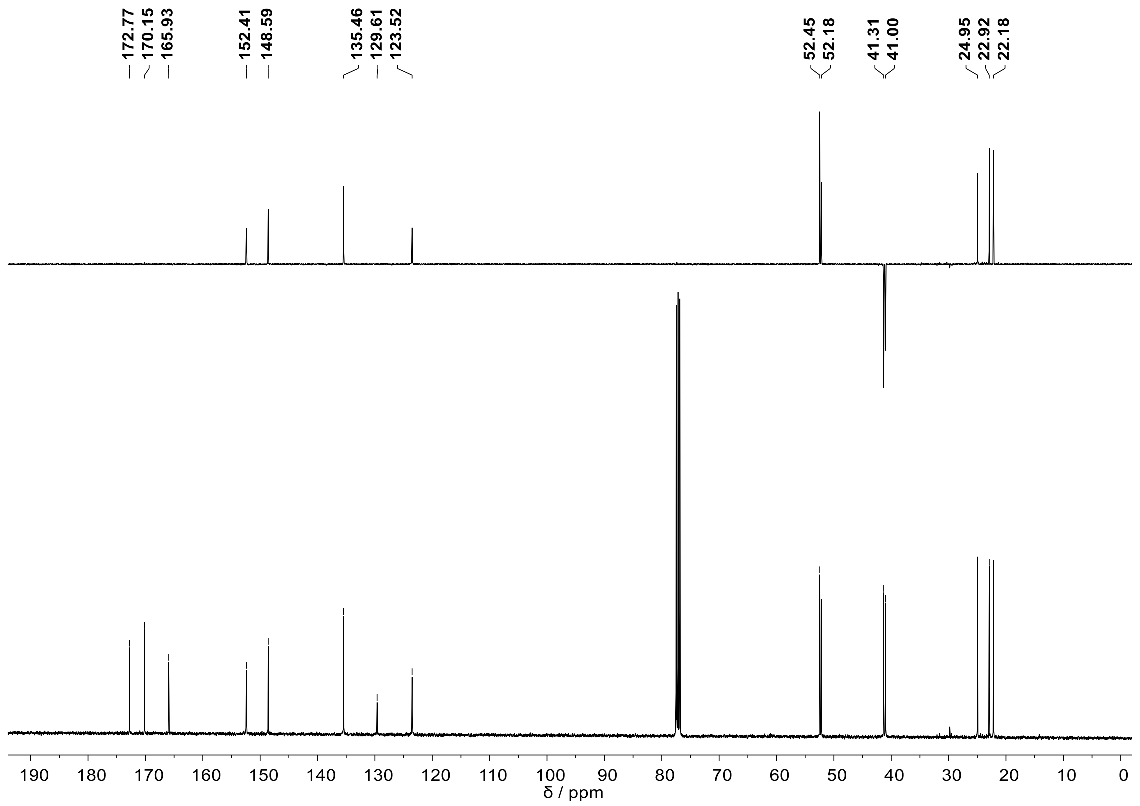

Figure S14. $^{13}C\{^{1}H\}$ and DEPT-135 NMR spectra ($CDCl_3$, 101 MHz) of compound **5c**.

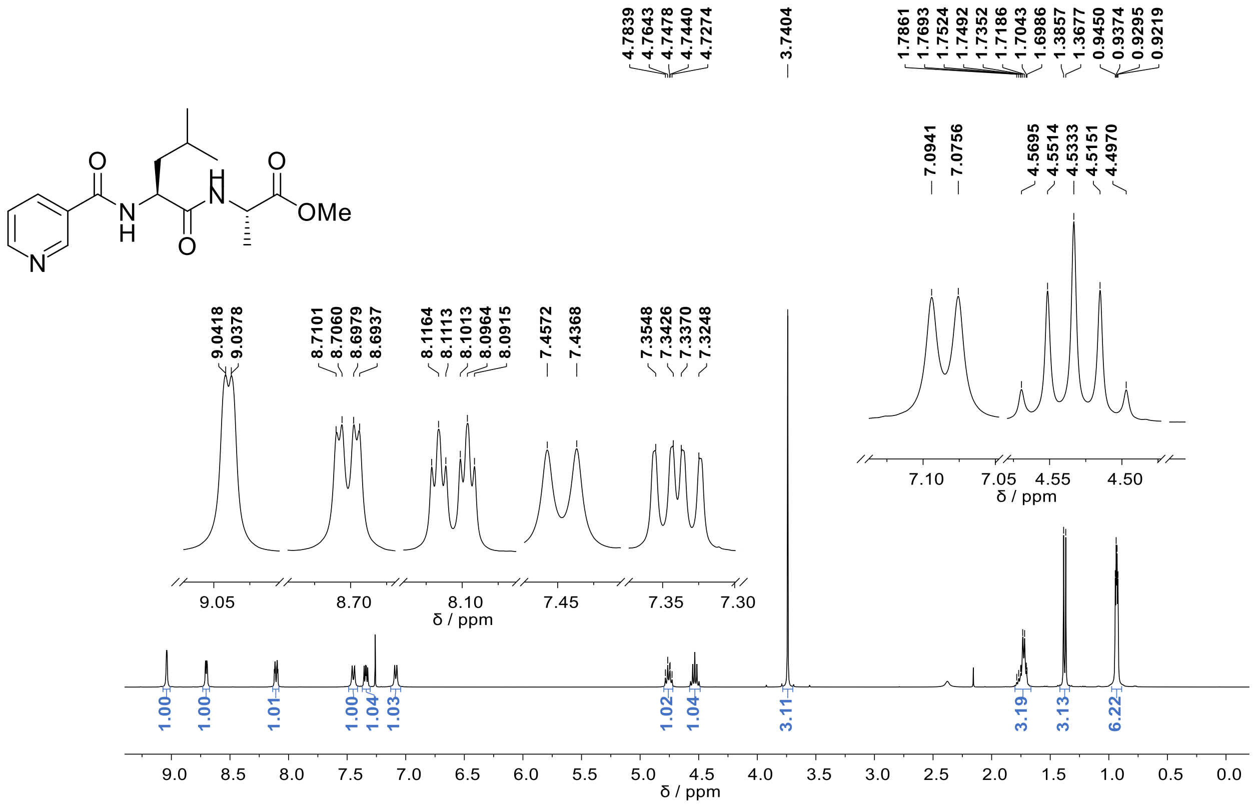

Figure S15. ^{1}H NMR spectrum ($CDCl_3$, 400 MHz) of compound **5d**.

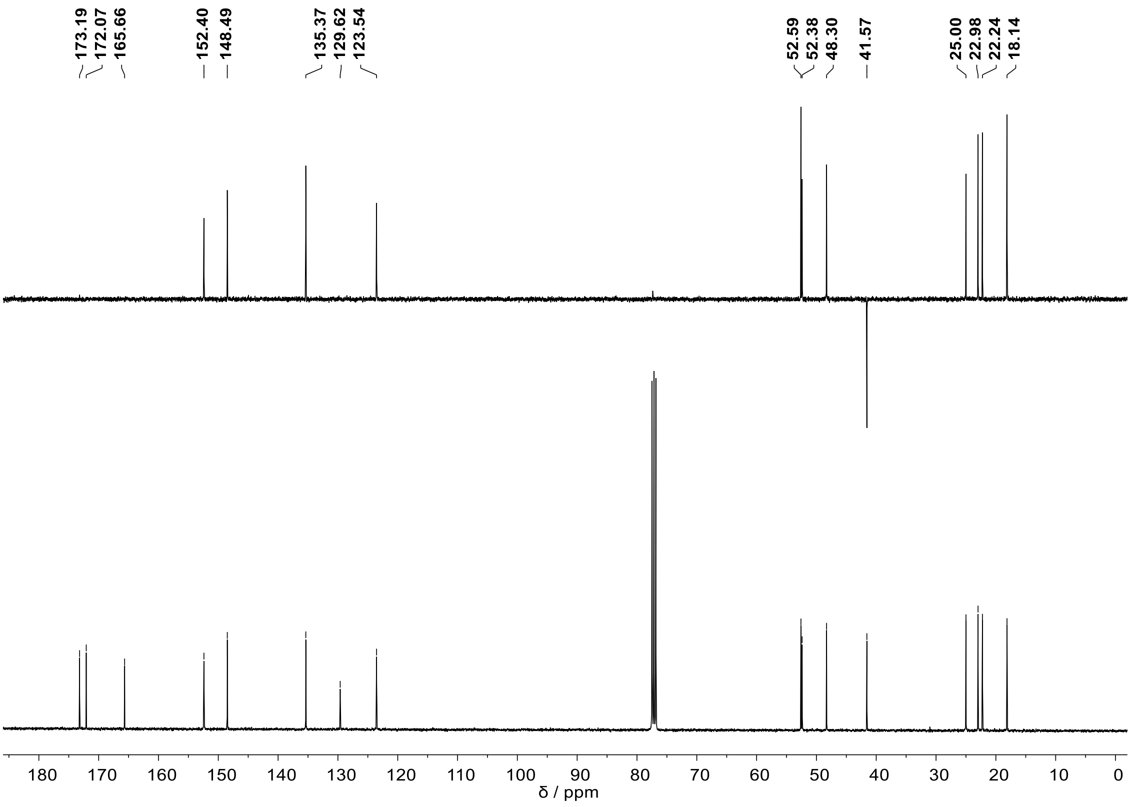

Figure S16. ^{13}C{^{1}H} and DEPT-135 NMR spectra ($CDCl_3$, 101 MHz) of compound **5d**.

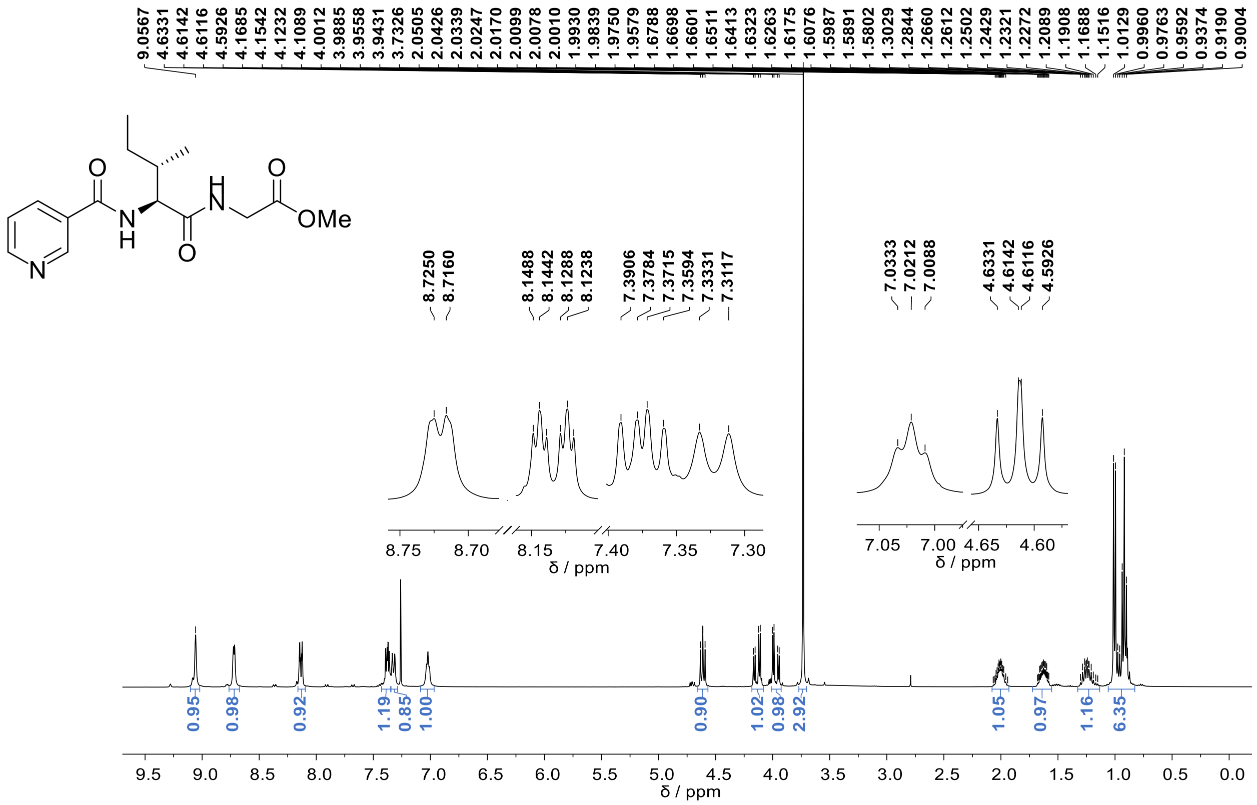

Figure S17. ^{1}H NMR spectrum ($CDCl_3$, 400 MHz) of compound **5e.**

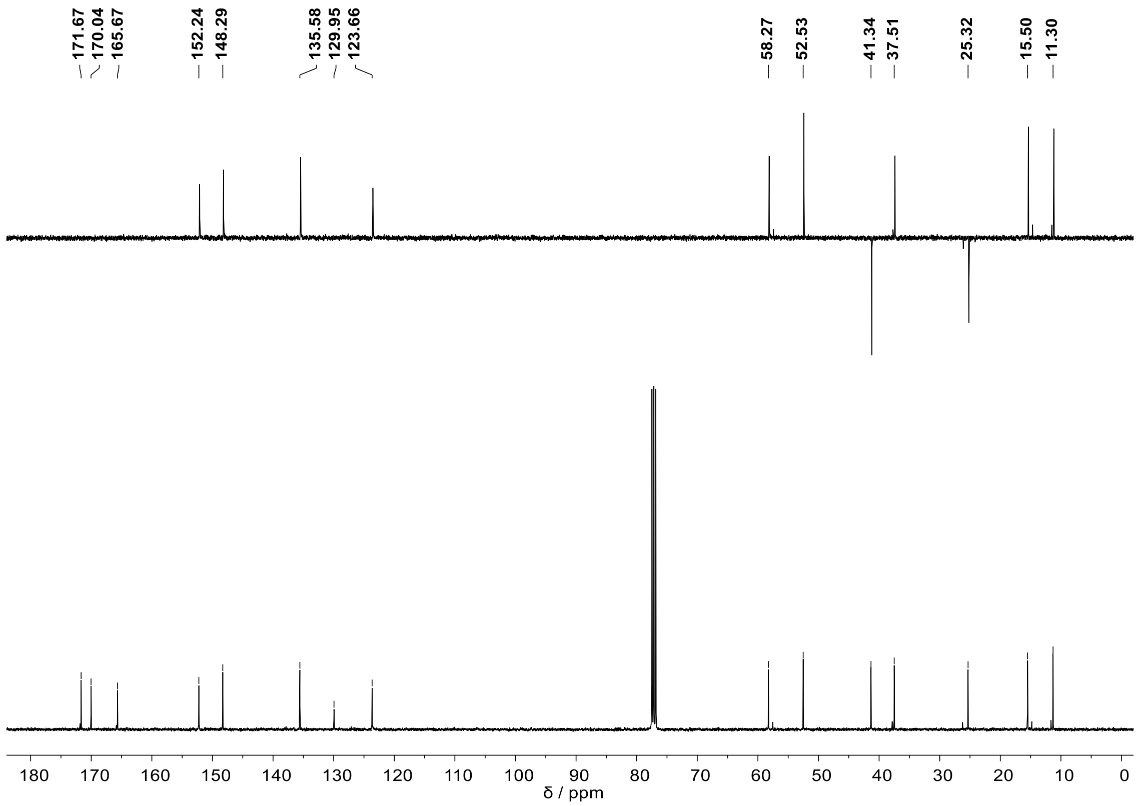

Figure S18. $^{13}C\{^{1}H\}$ and DEPT-135 NMR spectra ($CDCl_3$, 101 MHz) of compound **5e**.

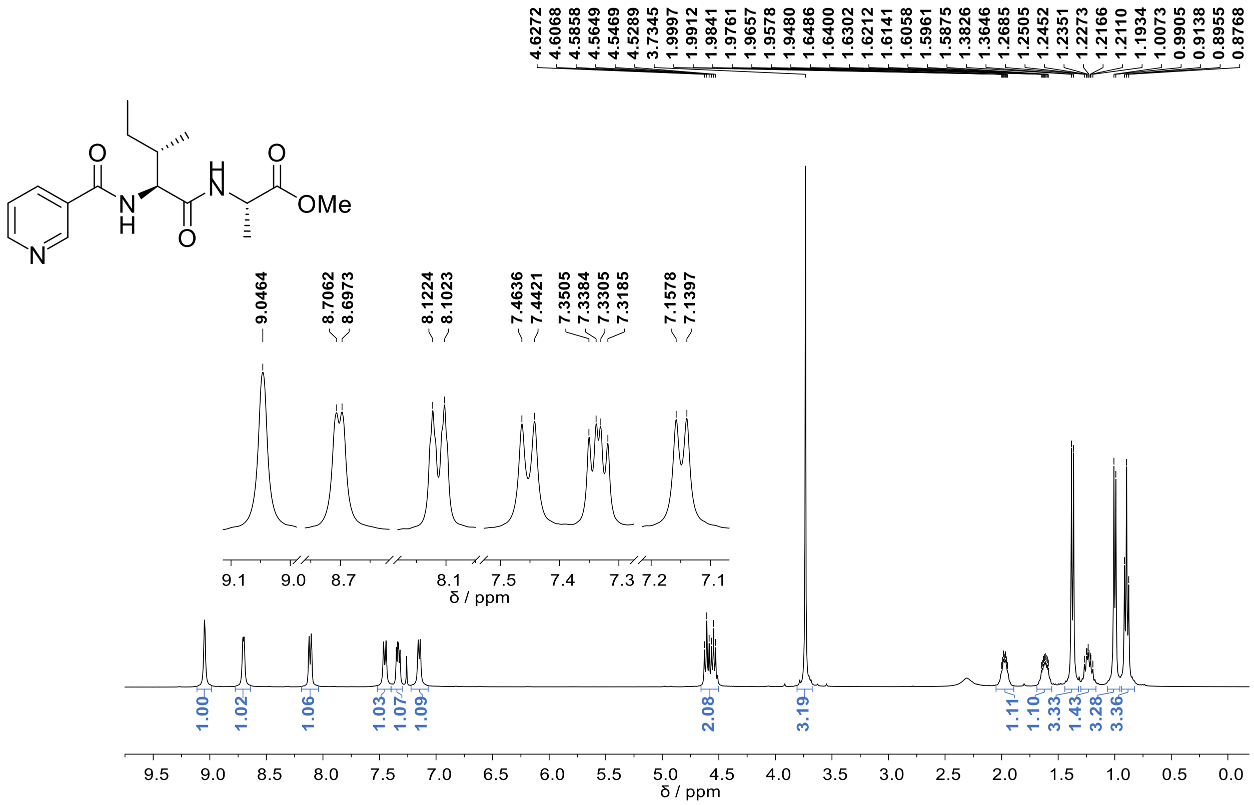

Figure S19. ^{1}H NMR spectrum ($CDCl_3$, 400 MHz) of compound **5f**.

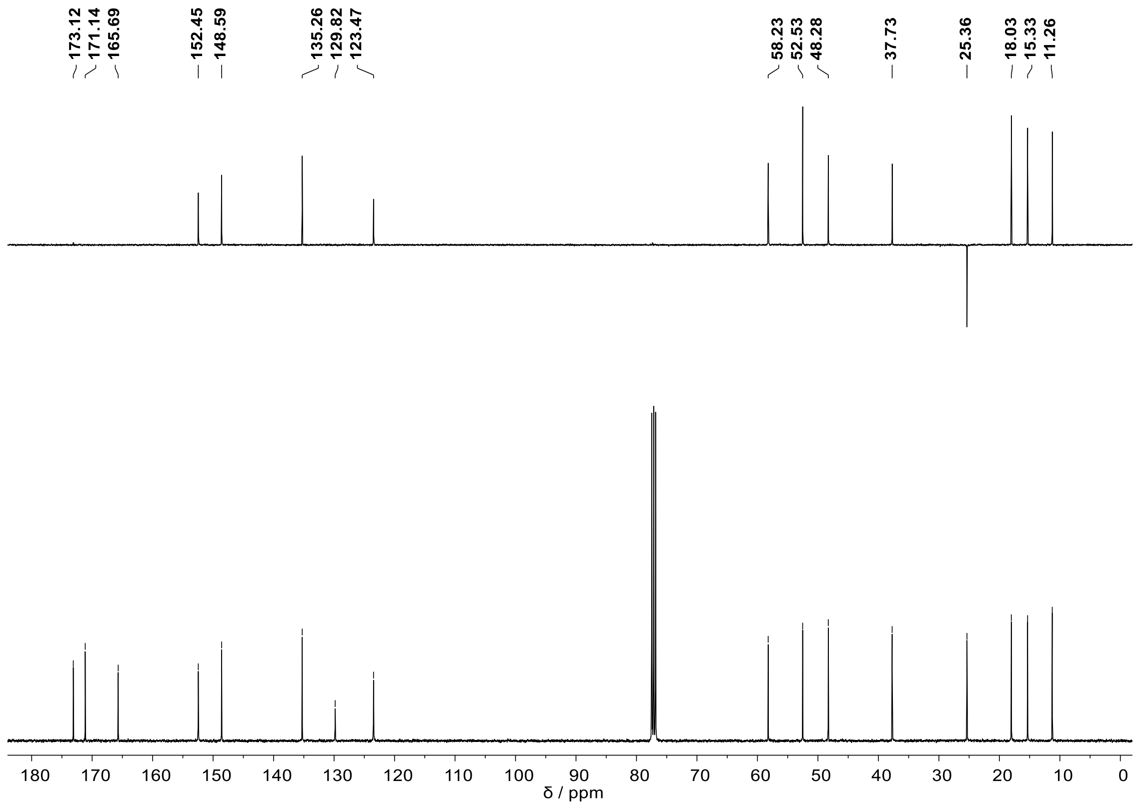

Figure S20. $^{13}C\{^{1}H\}$ and DEPT-135 NMR spectra ($CDCl_3$, 101 MHz) of compound **5f**.

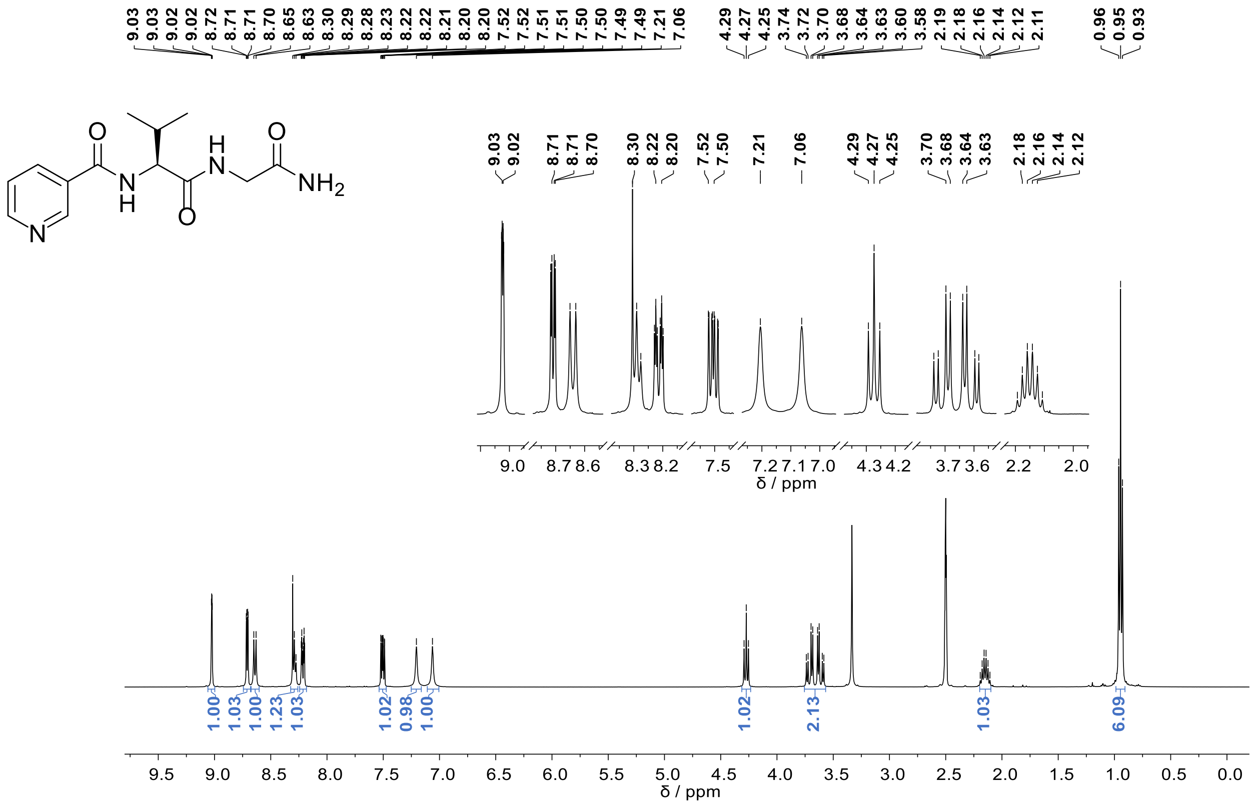

Figure S21. ^{1}H NMR spectrum (DMSO-d_6, 400 MHz) of compound **6a**.

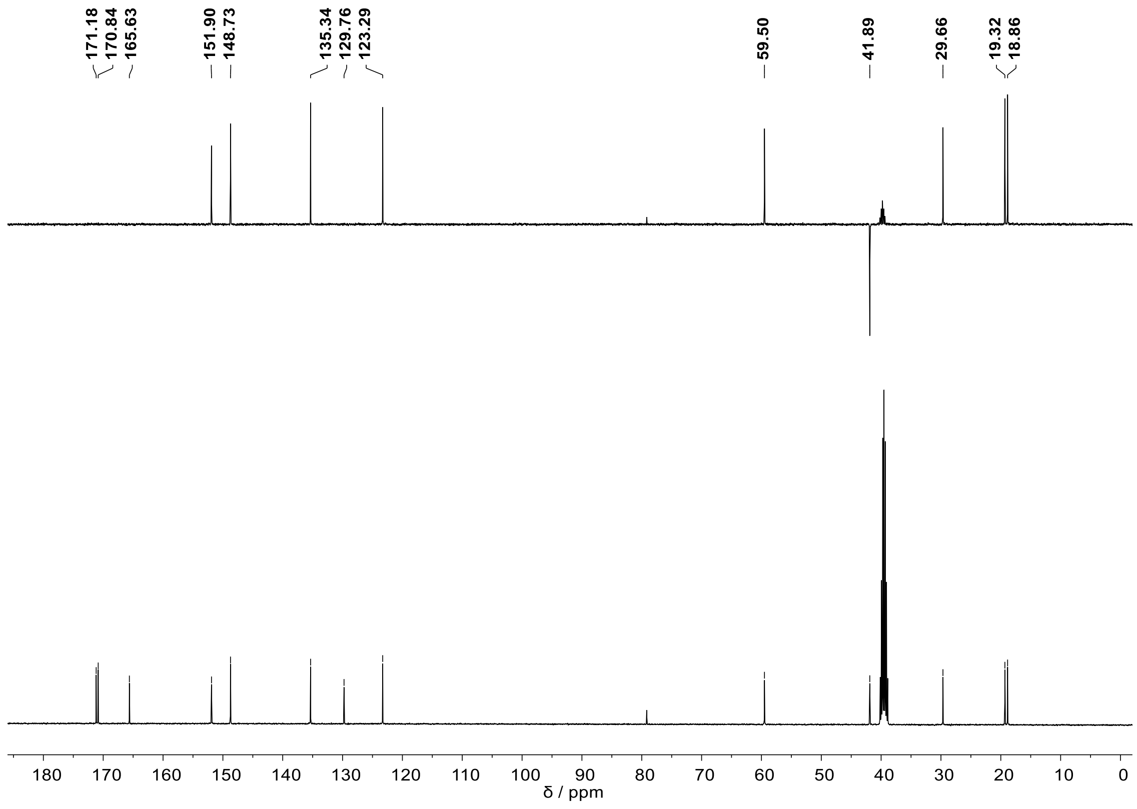

Figure S22. $^{13}C\{^{1}H\}$ and DEPT-135 NMR spectra (DMSO-d_6, 101 MHz) of compound **6a.**

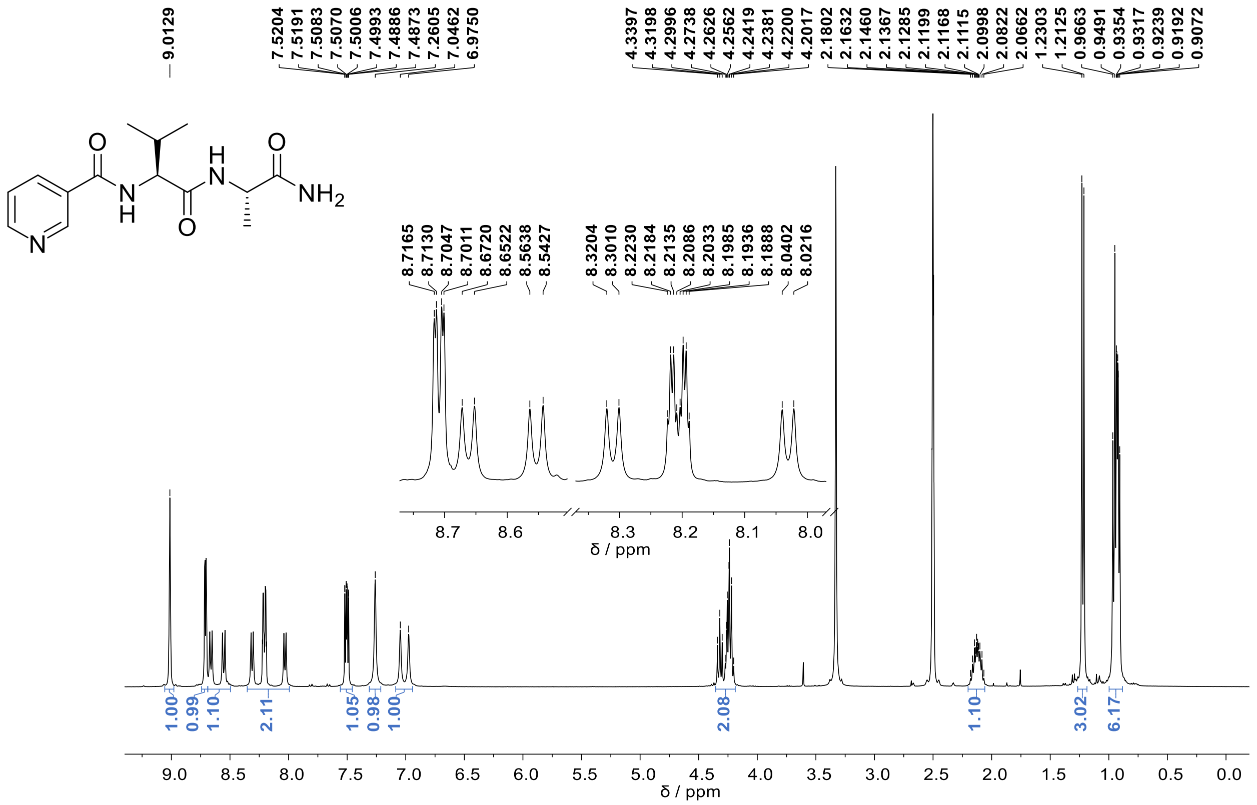

Figure S23. ^{1}H NMR spectrum (DMSO-d_6, 400 MHz) of compound **6b**.

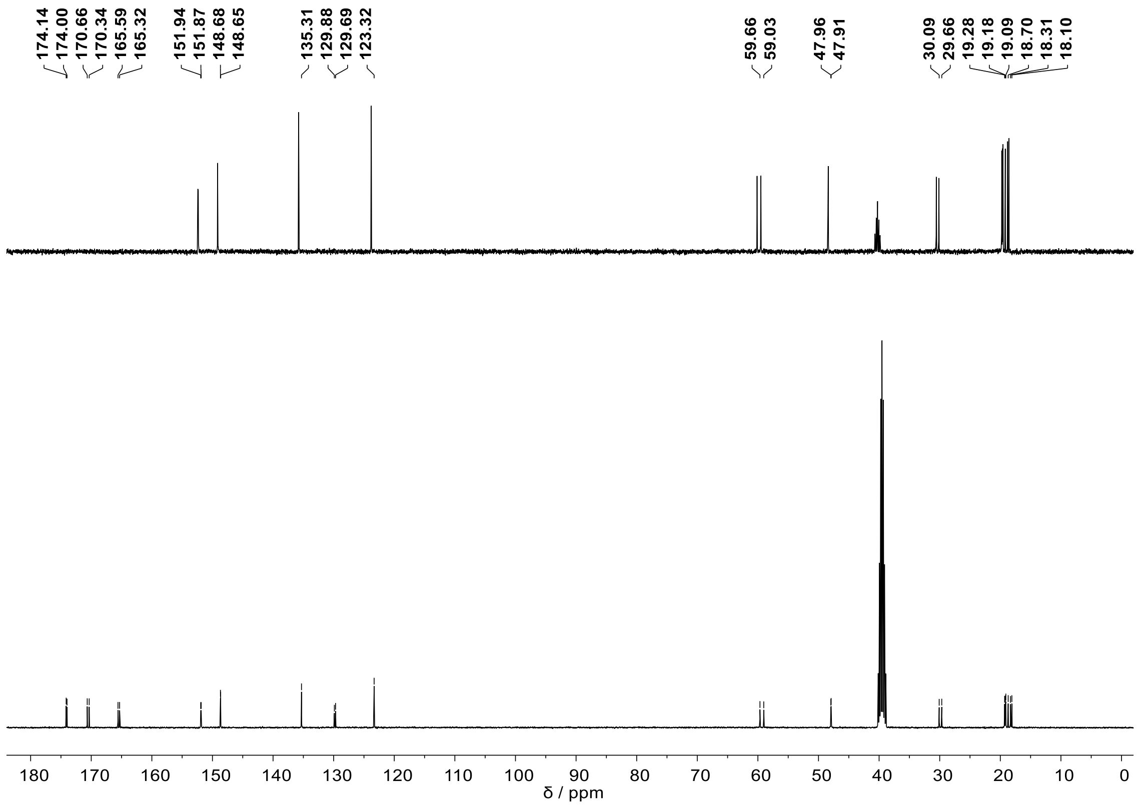

Figure S24. ^{13}C{^{1}H} and DEPT-135 NMR spectra (DMSO-d_6, 101 MHz) of compound **6b**.

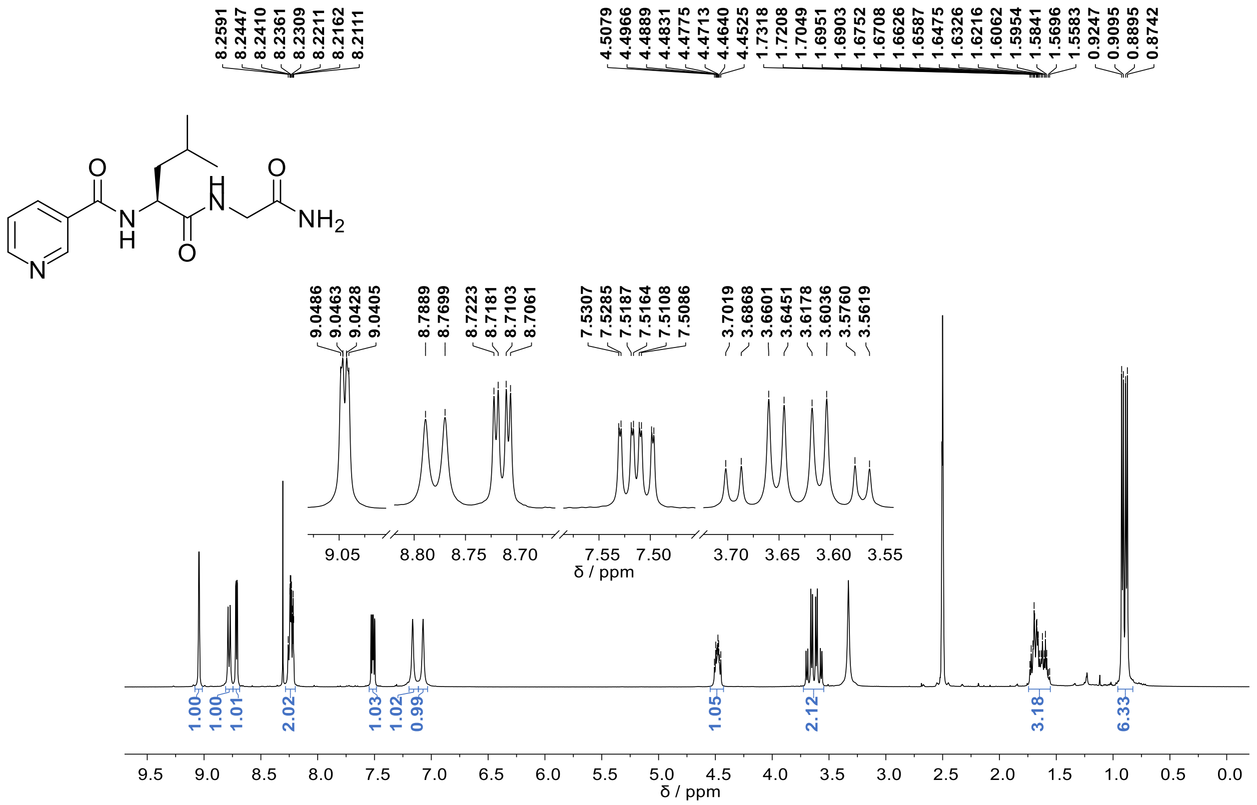

Figure S25. ^{1}H NMR spectrum (DMSO-d_6, 400 MHz) of compound **6c**.

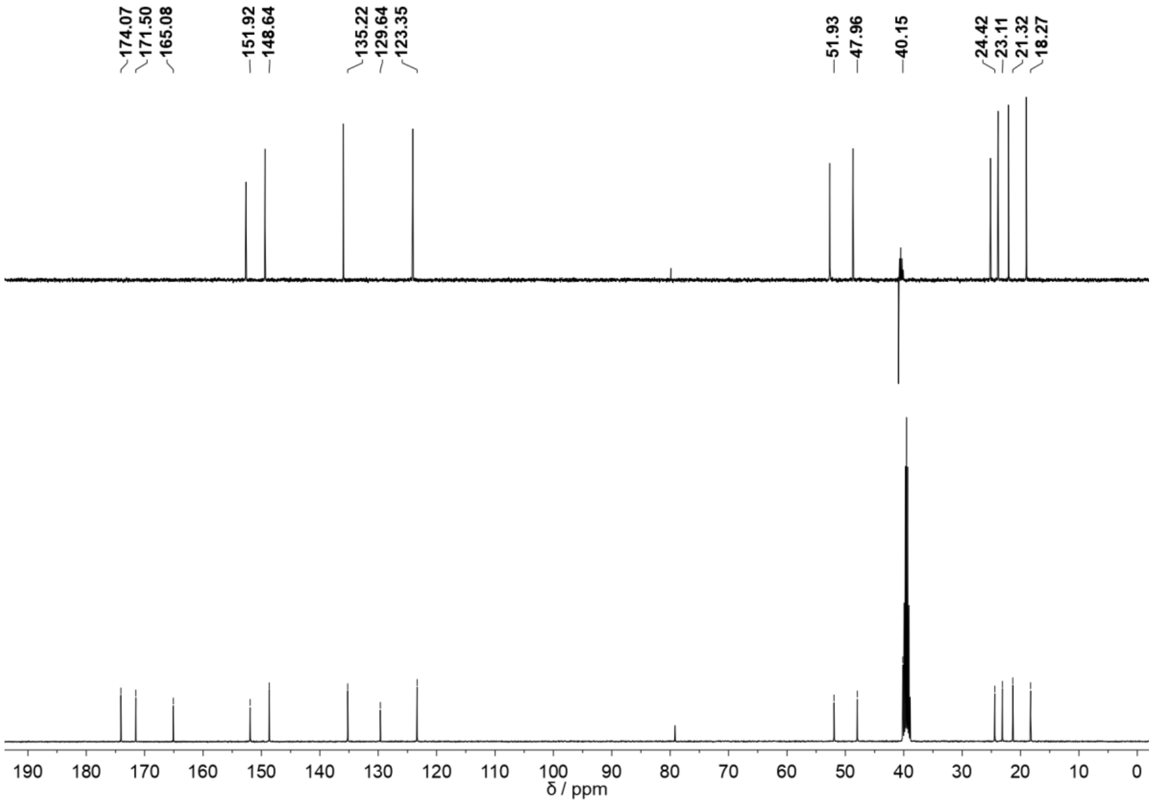

Figure S26. ^{13}C{^{1}H} and DEPT-135 NMR spectra (DMSO-d_6, 101 MHz) of compound **6c**.

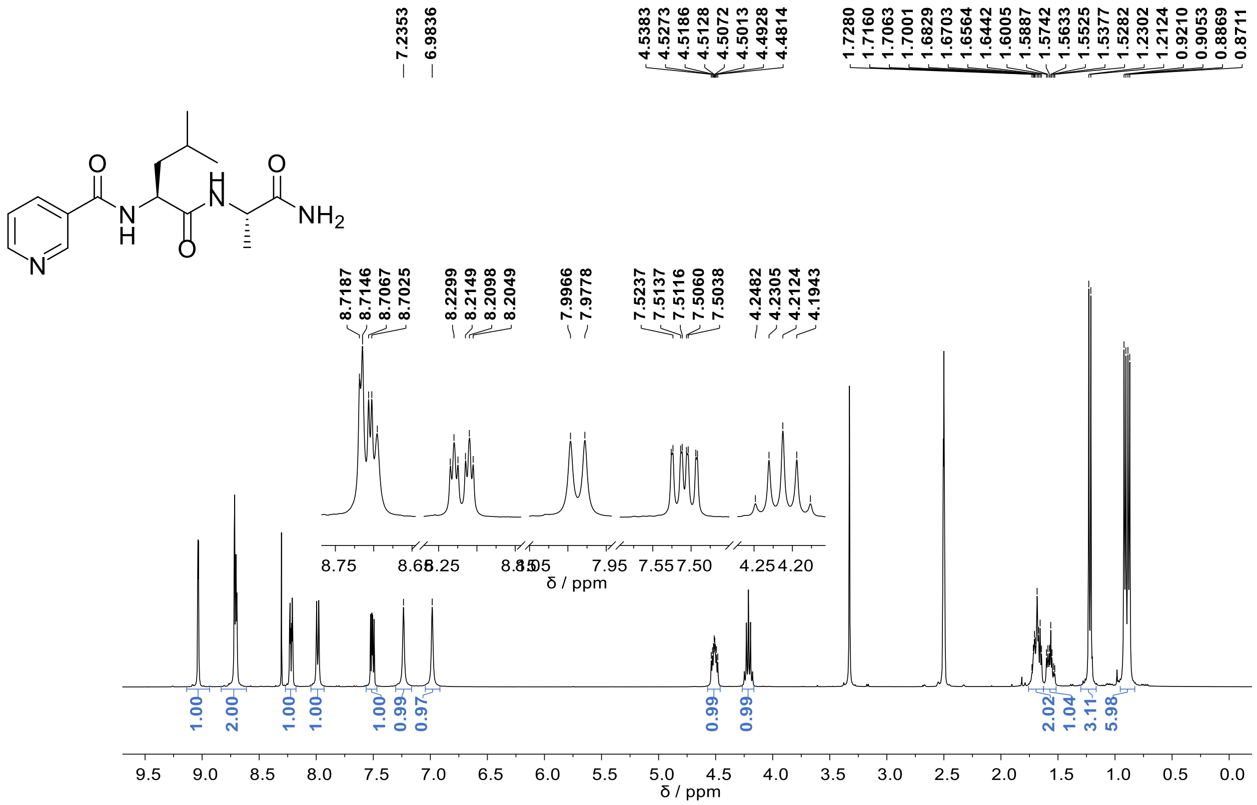

Figure S27. ^{1}H NMR spectrum (DMSO-d_6, 400 MHz) of compound **6d**.

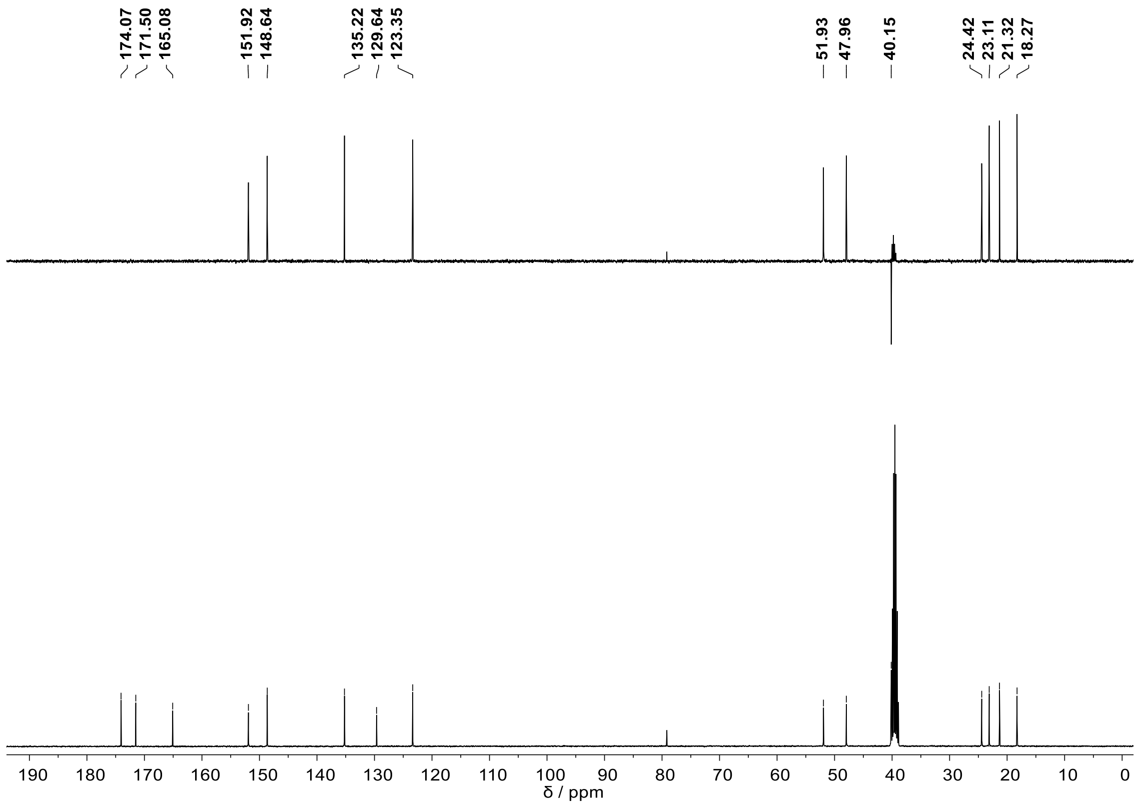

Figure S28. $^{13}C\{^{1}H\}$ and DEPT-135 NMR spectra (DMSO-d_6, 101 MHz) of compound **6d**.

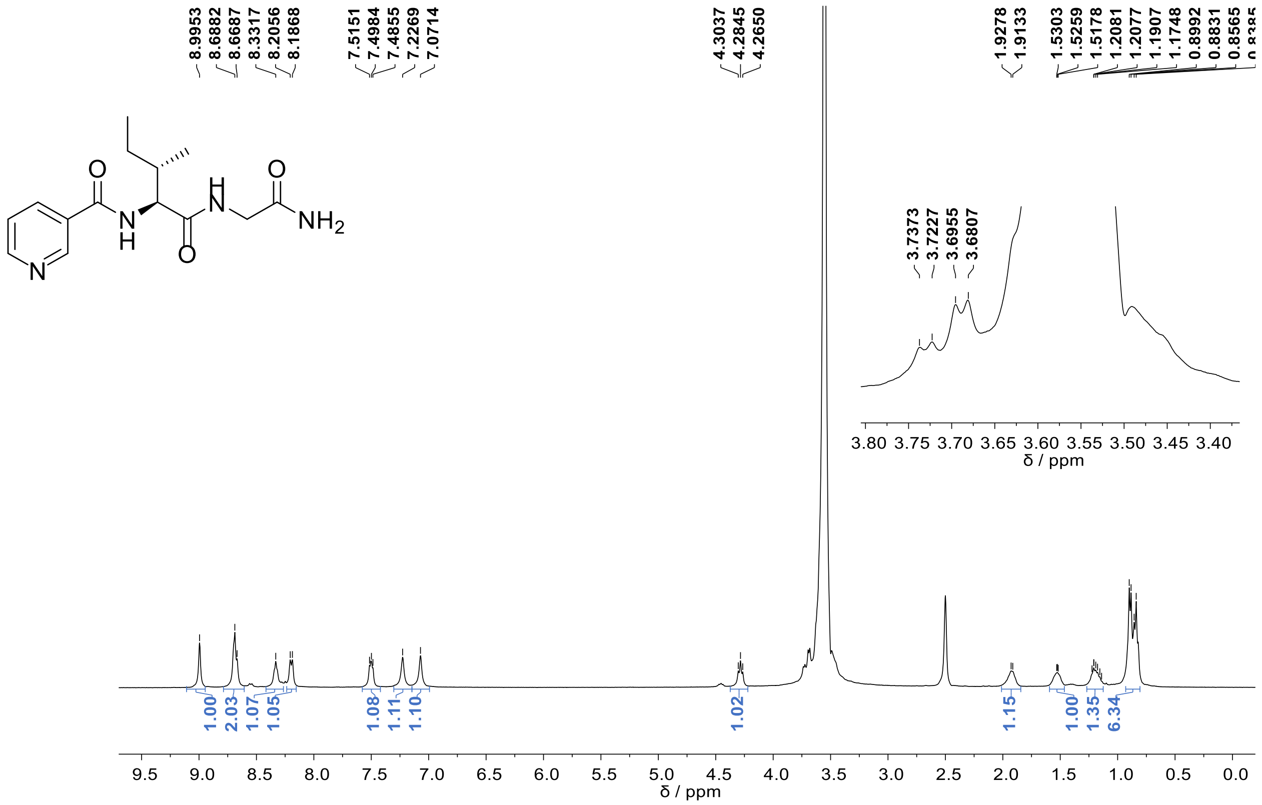

Figure S29. ^{1}H NMR spectrum (DMSO-d_6, 400 MHz) of compound **6e**.

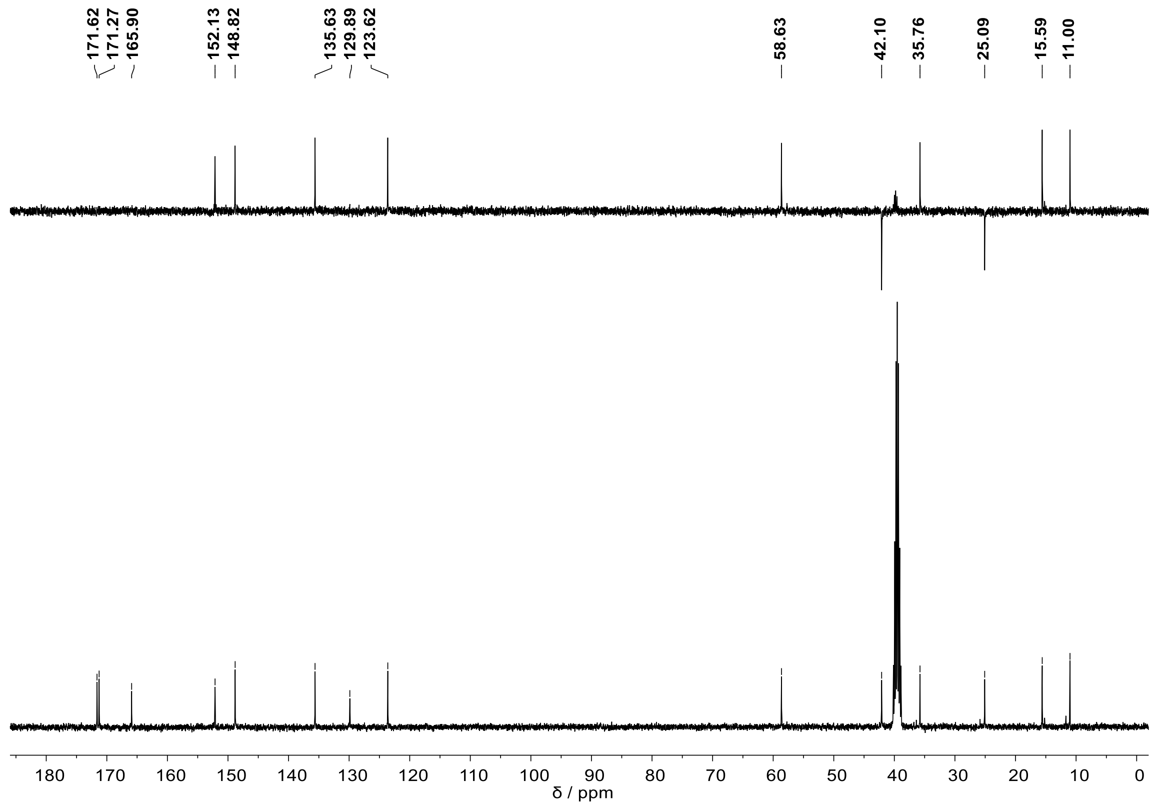

Figure S30. ^{13}C{^{1}H} and DEPT-135 NMR spectra (DMSO-d_6, 101 MHz) of compound **6e**.

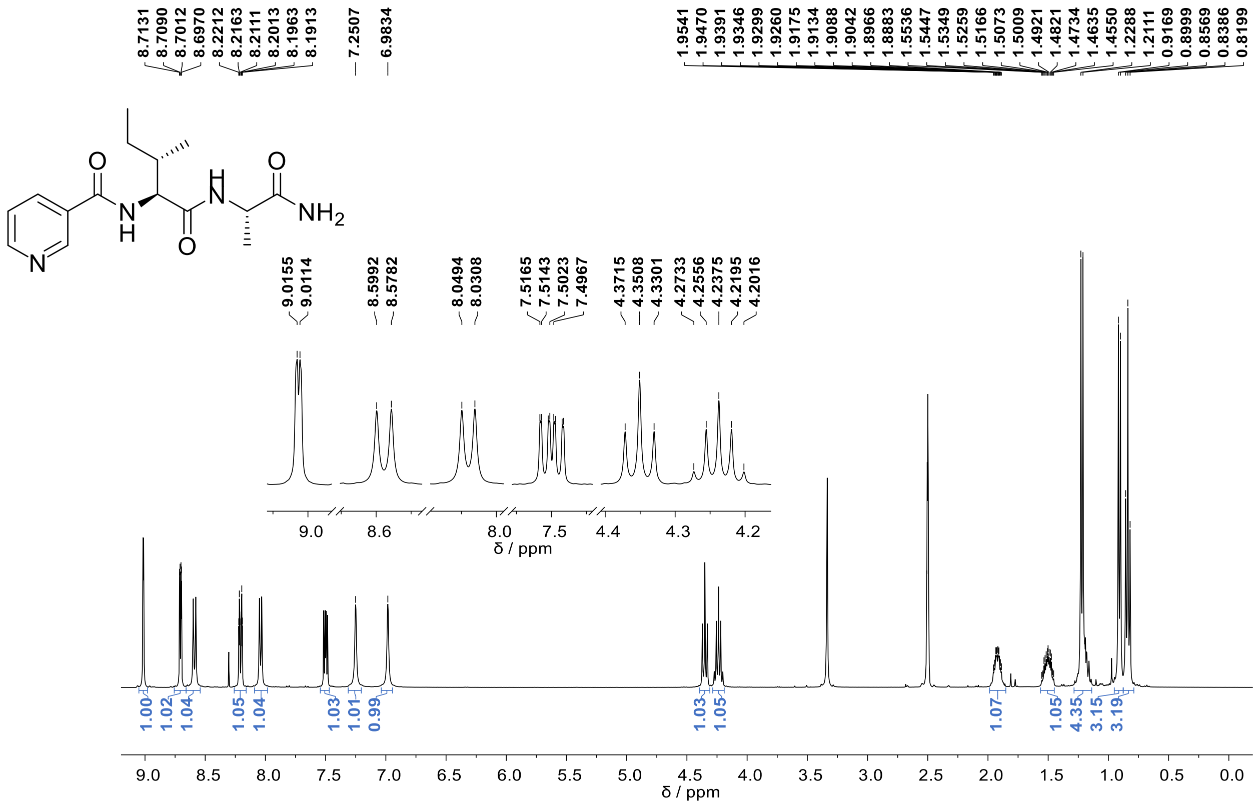

Figure S31. ^{1}H NMR spectrum (DMSO-d_6, 400 MHz) of compound **6f**.

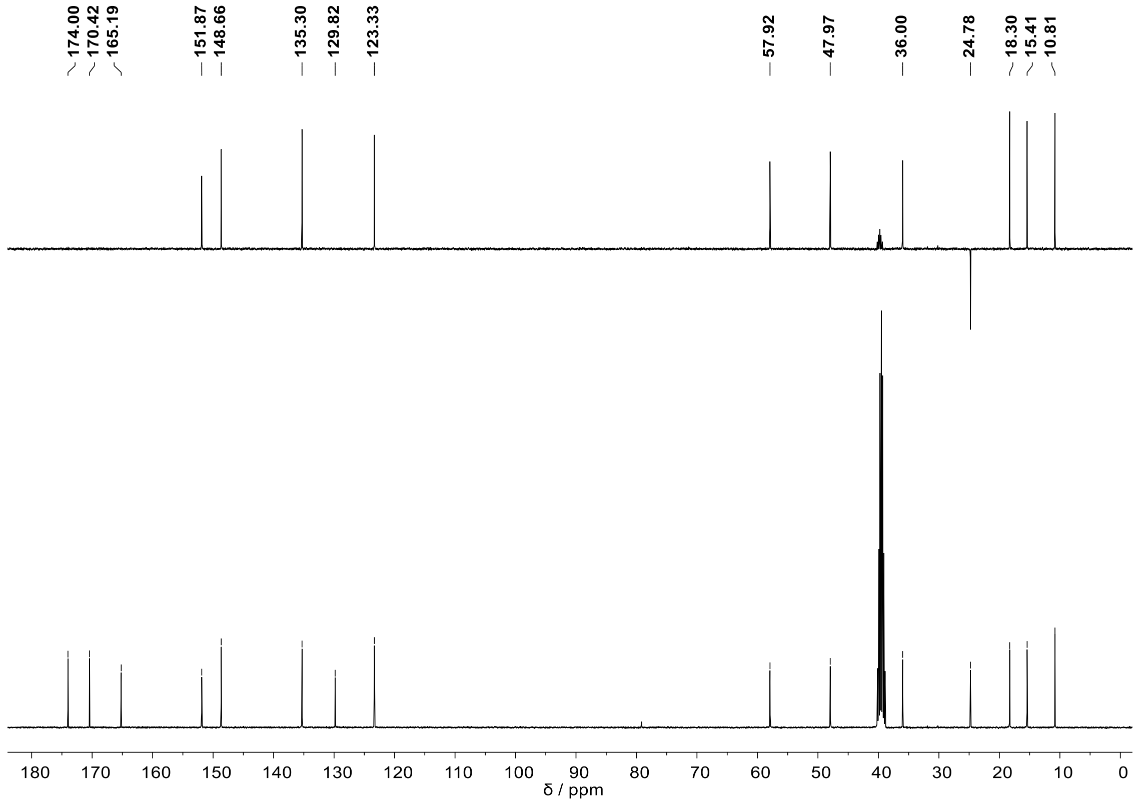

Figure S32. ^{13}C{^{1}H} and DEPT-135 NMR spectra (DMSO-d_6, 101 MHz) of compound **6f**.

2. HRMS Spectra for Compounds 5-6(a-f)

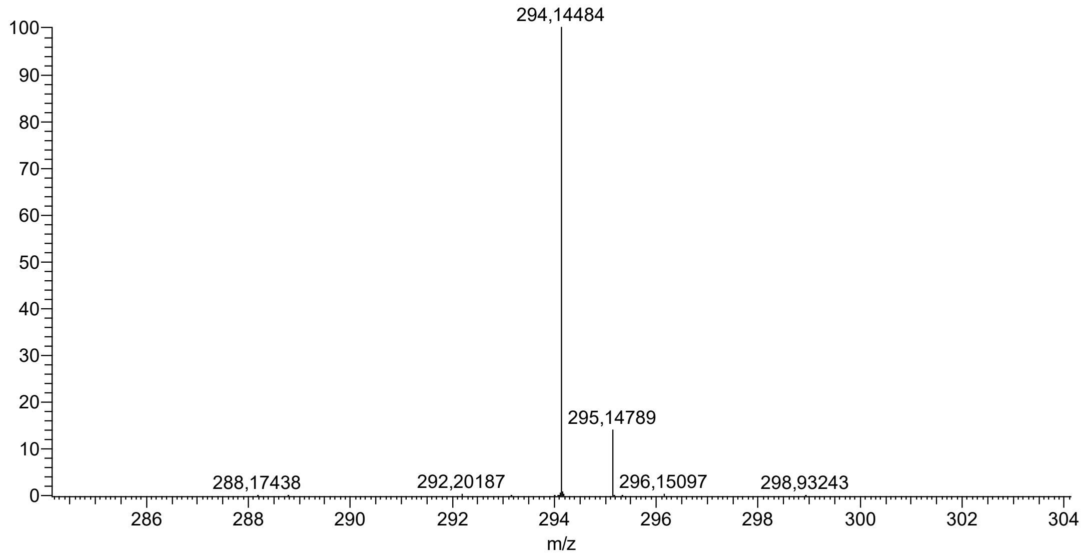

Figure S33. HRMS (ESI-TOF) *m/z* of **5a**. Calcd for $C_{14}H_{20}N_3O_4^+$: 294.14483; Found: 294.14484.

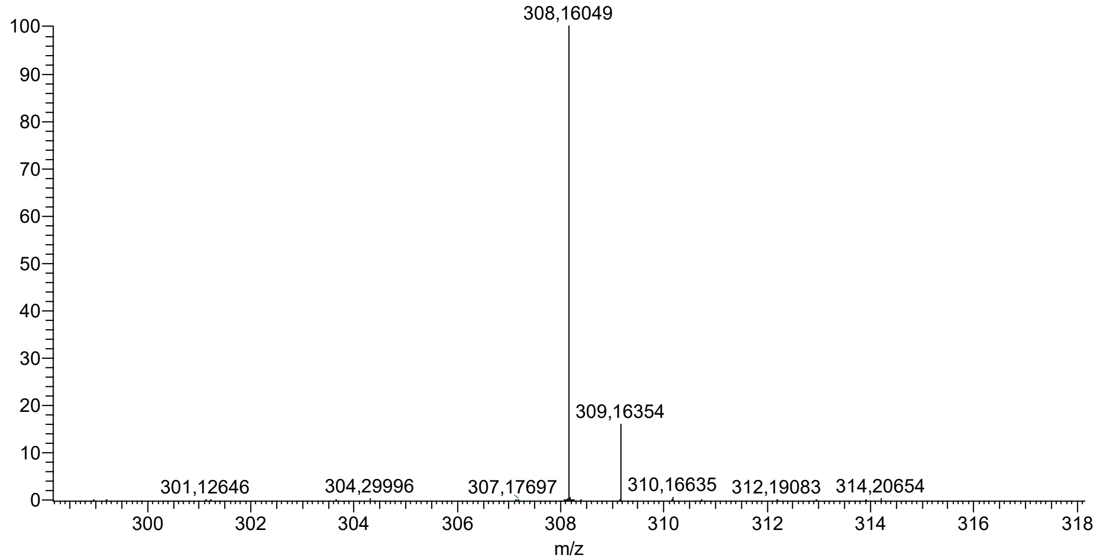

Figure S34. HRMS (ESI-TOF) *m/z* of **5b**. Calcd for $C_{15}H_{22}N_3O_4^+$: 308.16048; Found: 308.16049.

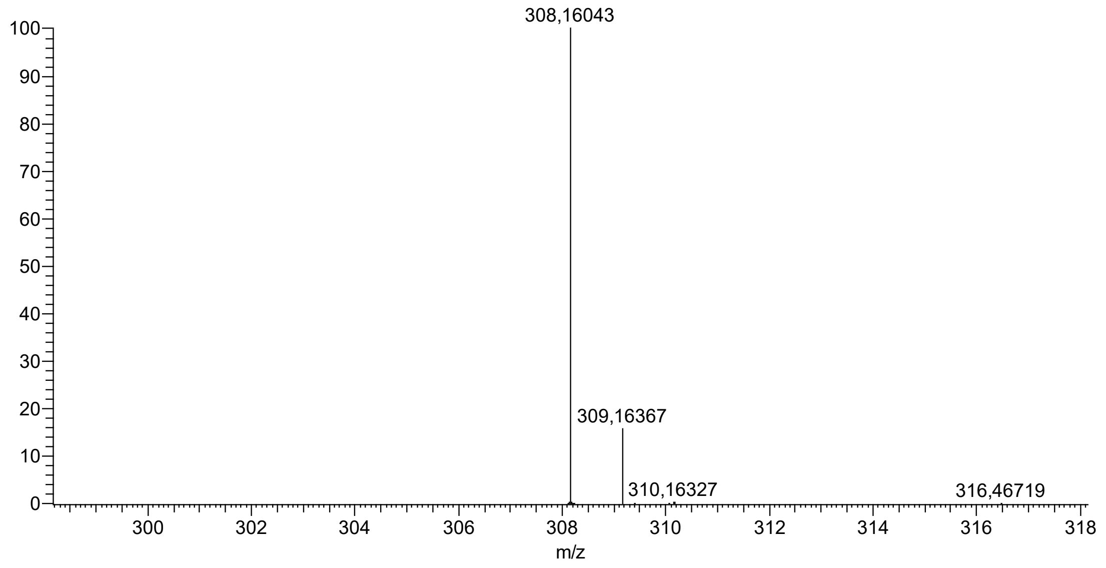

Figure S35. HRMS (ESI-TOF) *m*/*z* of **5c**. Calcd for $C_{15}H_{22}N_3O_4^+$: 308.16048; Found: 308.16043.

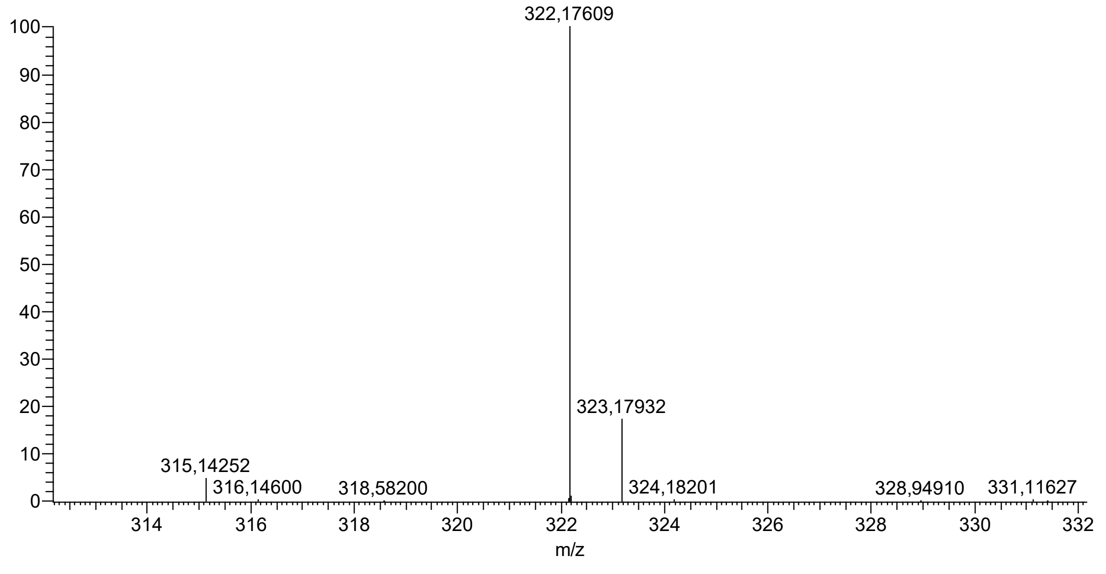

Figure S36. HRMS (ESI-TOF) *m*/*z* of **5d**. Calcd for $C_{16}H_{24}N_3O_4^+$: 322.17613; Found: 322.17609.

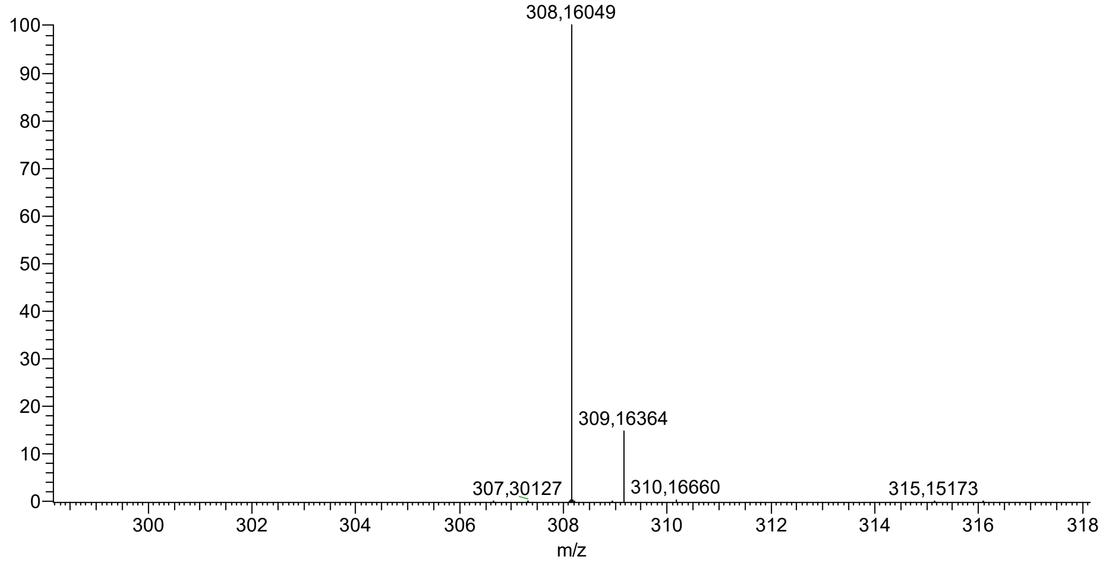

Figure S37. HRMS (ESI-TOF) *m*/*z* of **5e**. Calcd for $C_{15}H_{22}N_3O_4^+$: 308.16048; Found: 308.16049.

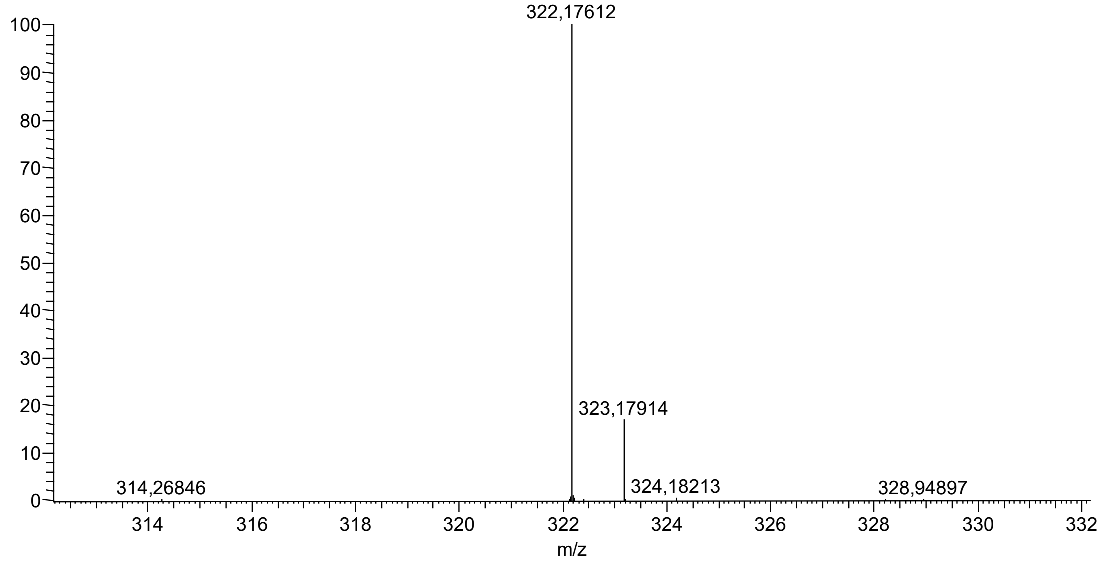

Figure S38. HRMS (ESI-TOF) *m*/*z* of **5f**. Calcd for $C_{16}H_{24}N_3O_4^+$: 322.17613; Found: 322.17612.

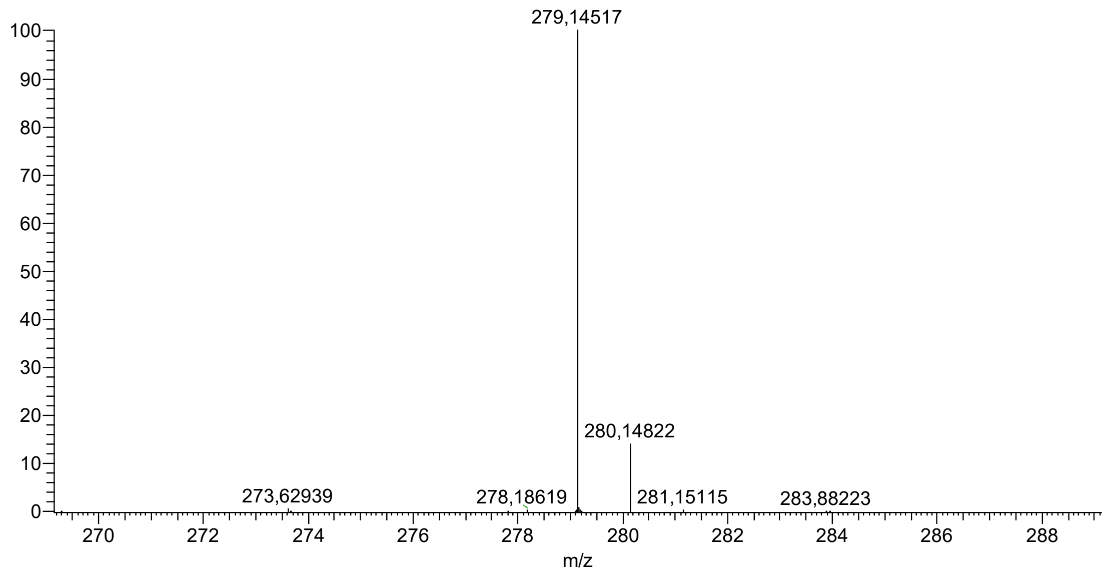

Figure S39. HRMS (ESI-TOF) *m*/*z* of **6a**. Calcd for $C_{13}H_{19}N_4O_3^+$: 279.14517; Found: 279.14517.

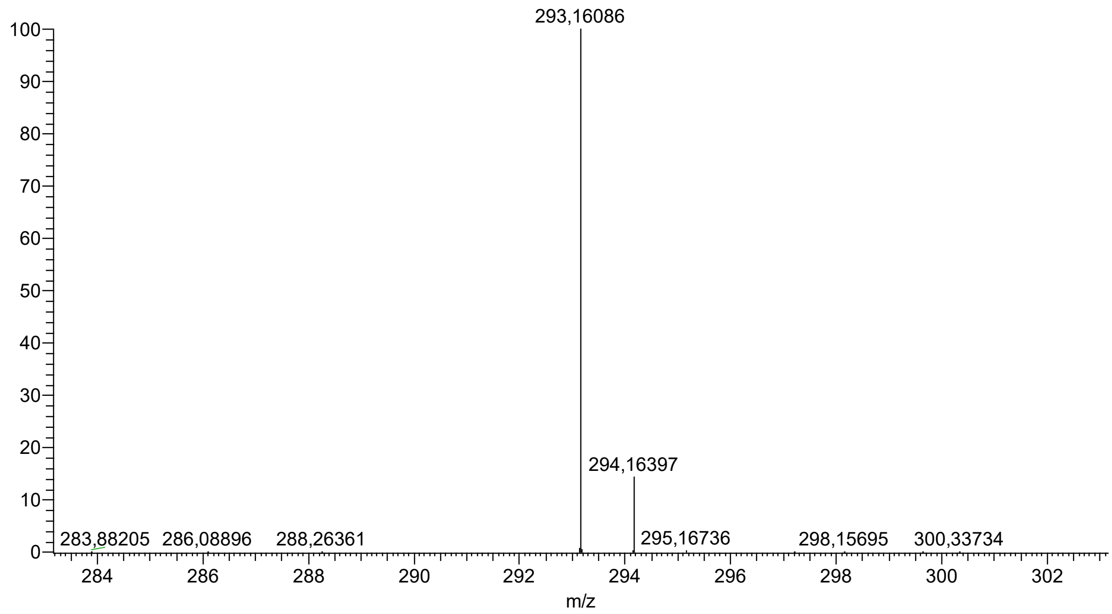

Figure S40. HRMS (ESI-TOF) *m*/*z* of **6b**. Calcd for $C_{14}H_{21}N_4O_3^+$: 293.16082; Found: 293.16086.

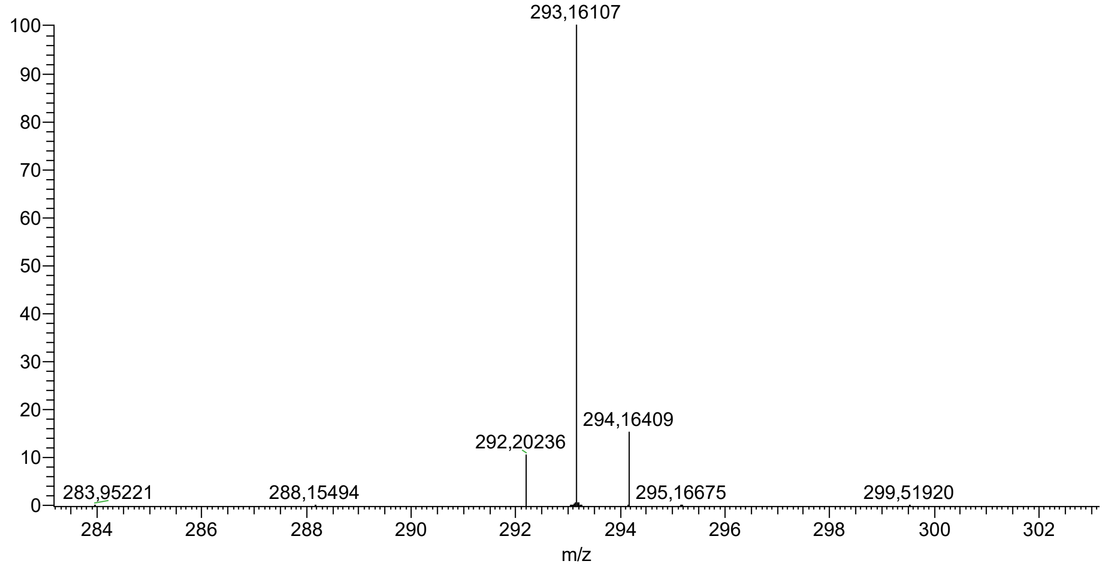

Figure S41. HRMS (ESI-TOF) *m*/*z* of **6c**. Calcd for $C_{14}H_{21}N_4O_3^+$: 293.16082; Found: 293.16107.

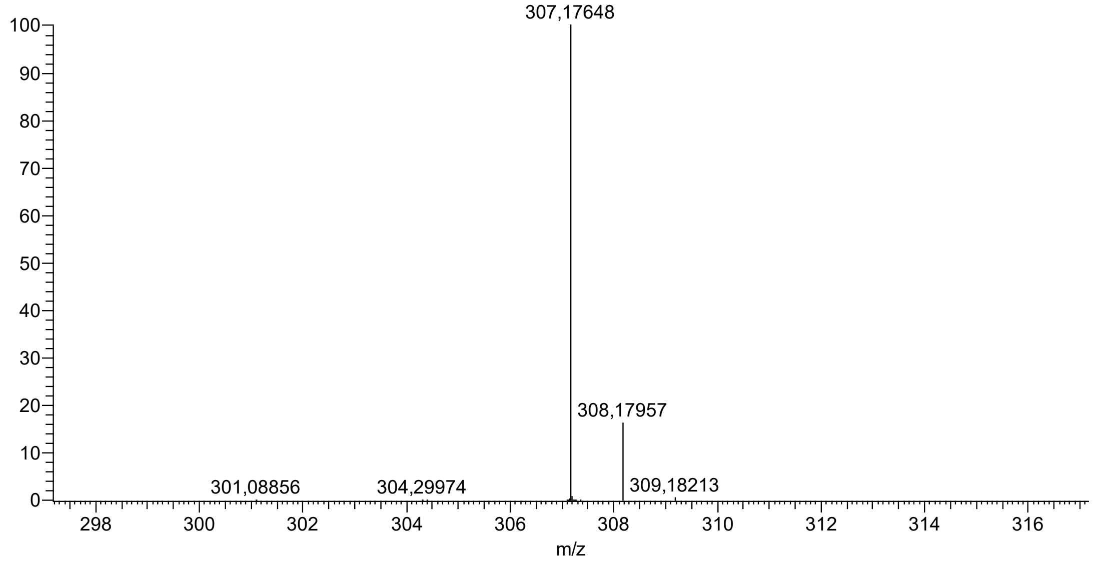

Figure S42. HRMS (ESI-TOF) *m*/*z* of **6d**. Calcd for $C_{15}H_{23}N_4O_3^+$: 307.17647; Found: 307.17648.

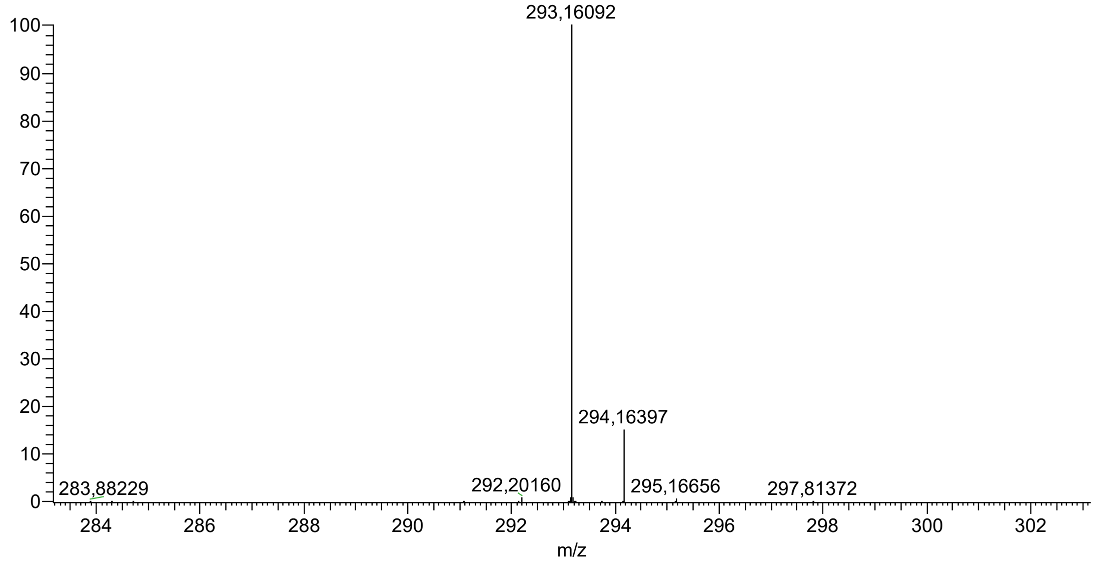

Figure S43. HRMS (ESI-TOF) *m*/*z* of **6e**. Calcd for $C_{14}H_{21}N_4O_3^+$: 293.16082; Found: 293.16092.

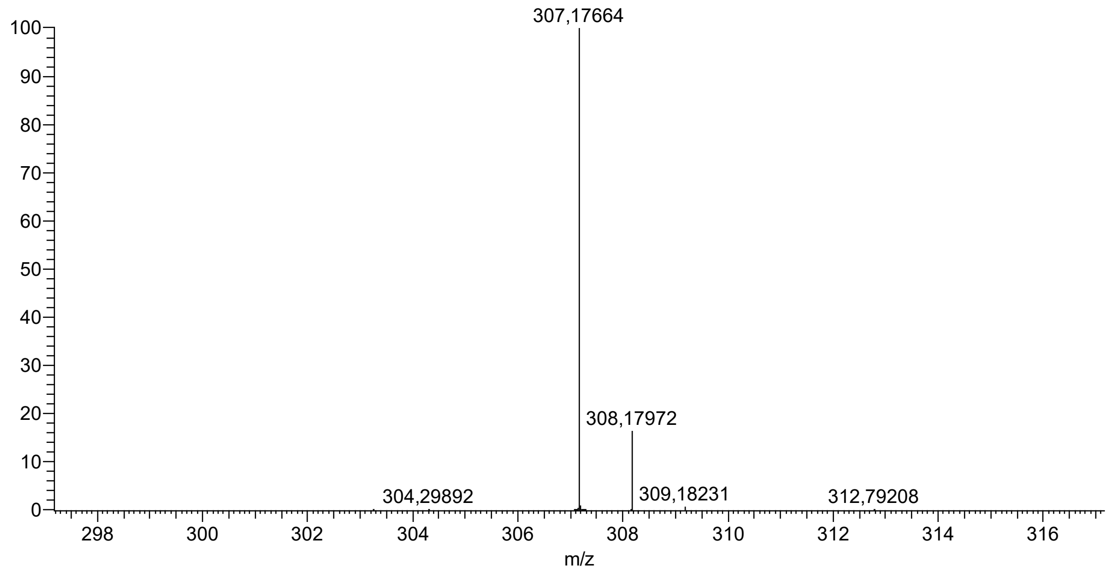

Figure S44. HRMS (ESI-TOF) *m*/*z* of **6f**. Calcd for $C_{15}H_{23}N_4O_3^+$: 307.17647; Found: 307.17664.

3. RP-HPLC Chromatogram for 6c

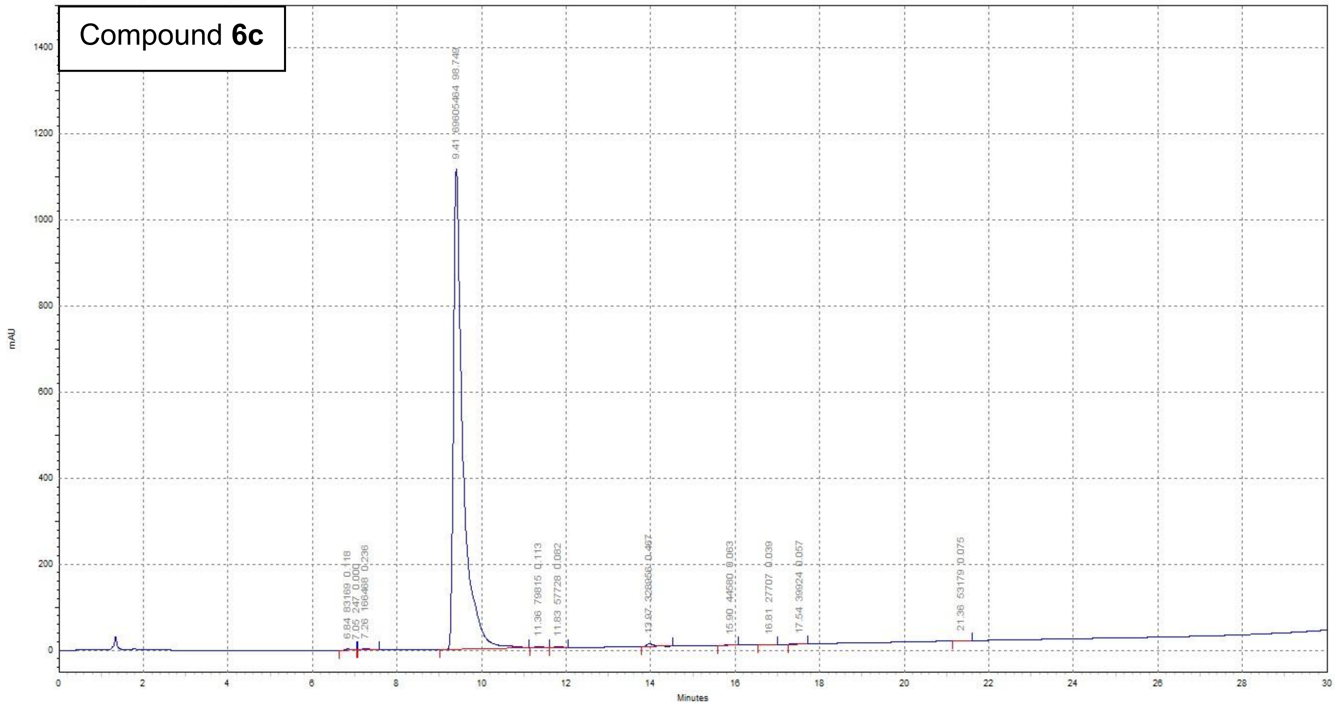

Figure S45. HPLC chromatogram of **6c**. R_t = 9.41 min, % area = 98.75%.

4. Data from Functional Assays at the hD_2R for Compounds 5-6(a-f) and MIF-1

Table S1. EC_{50} and E_{max} of DA in the absence and the presence of 0.01 nM and 1 nM of MIF-1 and Nic-based derivatives **5(a-f)** and **6(a-f)**.

**Compound**	**0.01 nM**		**1 nM**	
	EC_{50} (nM)	E_{max} (% of DA E_{max})	EC_{50} (nM)	E_{max} (% of DA E_{max})
**DA**	87.08 ± 24.87	100.00	87.08 ± 24.87	100.00
DA + **MIF-1**	92.92 ± 23.44	97.85	23.64 ± 6.73	93.97
DA + **5a**	61.70 ± 5.15	86.04	116.10 ± 60.46	94.77
DA + **5b**	32.18 ± 16.41	80.47	57.66 ± 39.78	105.40
DA + **5c**	35.70 ± 8.28	100.20	47.82 ± 5.17	83.13
DA + **5d**	49.89 ± 13.48	87.21	116.90 ± 48.16	94.23
DA + **5e**	108.80 ± 28.84	102.20	110.20 ± 46.82	93.95
DA + **5f**	71.67 ± 13.27	93.38	146.60 ± 51.11	97.43
DA + **6a**	115.70 ± 11.08	95.91	89.10 ± 13.22	89.77
DA + **6b**	47.44 ± 14.38	83.01	48.98 ± 12.47	96.26
DA + **6c**	17.03 ± 1.35	98.93	71.09 ± 12.71	84.78
DA + **6d**	65.35 ± 26.11	84.29	166.40 ± 73.6	104.10
DA + **6e**	74.07 ± 12.26	105.80	53.64 ± 8.96	87.79
DA + **6f**	22.89 ± 2.23	105.70	49.45 ± 13.73	85.34

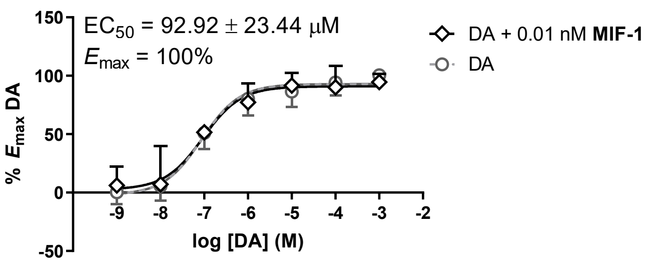

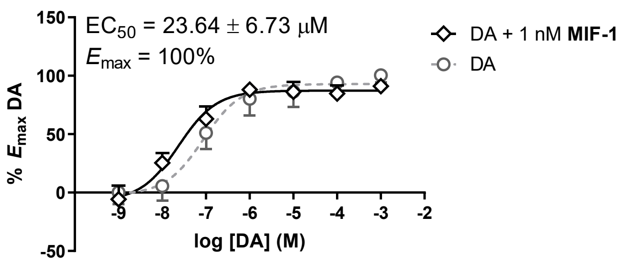

Figure S46. Concentration-response curve of DA in the presence of 0.01 nM and 1 nM of MIF-1 (including the concentration-response curve of dopamine).

5. Data from Neurotoxicity Evaluation at Dopaminergic Differentiated SH-SY5Y Cells for Compound I and MIF-1

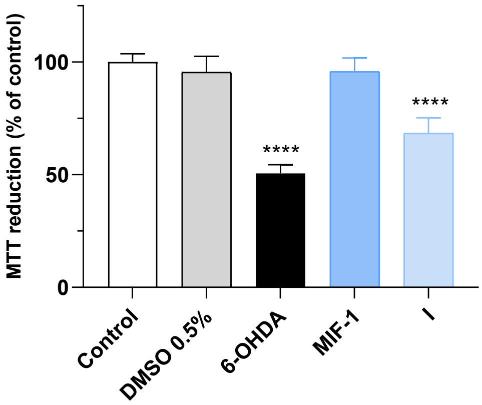

Figure S47. Neurotoxicity assessed by the MTT reduction assay in dopaminergic differentiated SH-SY5Y neuronal cells following a 48-h incubation with compound **I** and MIF-1 at 100 µM (all prepared in DMSO; final well concentration 0.5%), and with 6-OHDA at 100 µM (prepared in phosphate-buffered saline, PBS). Data are expressed as a percentage of the control condition (PBS) and are presented as mean ± standard deviation. The results were obtained from 9-15 wells across 3-4 independent experiments. Statistical analysis was performed using one-way ANOVA followed by Tukey's *post hoc* test (****$p < 0.0001$ vs. control).

6. Table of Cartesian Coordinates for 6c

Table S2. Cartesian coordinates of **6c**, at the PM6 level of theory in implicit water using the IEFPCM solvation model.

Atom	Cartesian coordinates			Atom	Cartesian coordinates		
	x	*y*	*z*		*x*	*y*	*z*
**C**	−4.15073	−2.65683	−0.11175	**H**	3.52253	−4.02279	−1.08452
**C**	−5.04136	−1.63534	−0.48460	**H**	5.06755	−3.62893	−0.33113
**C**	−2.85036	−2.31083	0.26134	**C**	1.68448	0.72896	0.24553
**C**	−2.47603	−0.96129	0.24875	**H**	1.50937	−1.10903	1.33490
**C**	−3.42936	0.00956	−0.12347	**O**	2.41726	1.22351	1.08376
**N**	−4.69039	−0.32207	−0.49283	**N**	1.1635	1.49350	−0.80075
**C**	−1.11681	−0.52004	0.67705	**C**	1.27669	2.96046	−0.75871
**O**	−0.90424	0.25768	1.59168	**C**	0.00211	3.67200	−0.28860
**N**	−0.06851	−1.03367	−0.09452	**H**	2.12345	3.26084	−0.08459
**C**	1.35234	−0.78339	−0.26274	**H**	1.53752	3.34333	−1.77523
**C**	2.28912	−1.55595	−0.67941	**O**	−0.37339	4.70474	−0.82031
**C**	3.51186	−2.15197	0.06116	**N**	−0.72176	3.10255	0.74606
**H**	2.63952	−0.90554	−1.50576	**H**	−0.24534	−1.66396	−0.87256
**H**	1.74076	−2.38199	−1.17633	**H**	0.48294	1.11678	−1.45146
**C**	4.50493	−1.06366	0.46923	**H**	−1.51342	3.60020	1.12780
**H**	4.91452	−0.53082	−0.39531	**H**	−0.40161	2.30251	1.28329
**H**	5.35173	−1.48201	1.02383	**H**	−2.14080	−3.08052	0.56902
**H**	4.04049	−0.30464	1.11920	**H**	−4.47381	−3.69607	−0.10822
**C**	4.19361	−3.19592	−0.83314	**H**	−6.07351	−1.85773	−0.78611
**H**	3.14822	−2.66719	0.98621	**H**	−3.18186	1.08112	−0.12511
**H**	4.55174	−2.75773	−1.77134				

7. Physicochemical Properties Analysis and Drug-Likeness Assessment

Evaluation of the physicochemical properties of novel drug candidates is of great importance to qualitatively estimate their oral bioavailability and membrane permeability.[1, 2]

The physicochemical properties of MIF-1 and the active peptidomimetics **5b**, **5c**, **6c**, and **6f** were evaluated using established descriptors commonly applied for drug-likeness and oral absorption profiling. These include molecular weight (MW), calculated octanol/water partition coefficient (clog*P*), number of hydrogen bond acceptors (HBA), hydrogen bond donors (HBD), total hydrogen bonding capacity (HBA + HBD), number of rotatable bonds (nRotB), and topological polar surface area (^{t}PSA). These parameters provide insight into molecular size, lipophilicity, hydrogen bonding potential, and conformational flexibility, all of which influence membrane permeability and developability.

Drug-likeness assessment was performed according to Lipinski's "rule of five" and the Veber criteria for oral absorption. Lipinski's rules stipulate that compounds with MW ≤ 500 Da, clog*P* ≤ 5, HBA ≤ 10, and HBD ≤ 5 are generally associated with good oral bioavailability.[1] Veber's criteria focus on molecular flexibility and polarity, recommending nRotB ≤ 10 and either ^{t}PSA ≤ 140 Å² or total hydrogen bonding capacity (HBA + HBD) ≤ 12.[2]

The calculated properties of MIF-1 and the Nic-based derivatives are summarized in Table S3.

Table S3. Calculated drug-like properties for MIF-1 and peptidomimetics **5b, 5c, 6c,** and **6f**. ^{a}Properties calculated using cheminformatics software [http://www.molinspiration.com].

Compound	MW^{a}	clog*P*^{a}	HBA^{a}	HBD^{a}	HBA + HBD	nRotB^{a}	^{t}PSA^{a} / Å^{2}
**5b**	307.35	0.30	7	2	9	7	97.39
**5c**	307.35	0.50	7	2	9	8	97.39
**6c**	292.34	–0.63	7	4	11	7	114.18
**6f**	306.37	0.23	7	4	11	7	114.18
**MIF-1**	284.36	–0.93	7	5	12	7	113.32
**Guidelines**[1, 2]	≤ 500	≤ 5	≤ 10	≤ 5	≤ 12	≤ 10	≤ 140

All compounds display molecular weights well below 500 Da, indicating a favorable size for absorption. The calculated clog*P* values range from −0.93 to 0.50, reflecting overall low to moderate lipophilicity. The use of Nic as a Pro surrogate results in a modest increase in lipophilicity relative to the parent neuropeptide (**6c** vs MIF-1), while remaining within the acceptable drug-like range.

Hydrogen bond acceptors are constant across all compounds (HBA = 7), whereas hydrogen bond donors are reduced in the peptidomimetics compared to MIF-1 (HBD = 2-4 vs 5), leading to a lower overall hydrogen bonding capacity. This reduction is generally associated with improved passive membrane permeability. All compounds satisfy Veber's limits for hydrogen bonding (HBA + HBD ≤ 12) and rotatable bonds (nRotB ≤ 10), although compound **5c** approaches the upper limit for conformational flexibility (nRotB = 8).

Topological polar surface area values range from 97.39 to 114.18 Å^2, well below the Veber threshold of 140 Å^2. These values support the conclusion that all compounds maintain an acceptable balance between polarity and lipophilicity, consistent with favorable oral absorption profiles.

Taken together, these results indicate that the designed peptidomimetics occupy a favorable region of drug-like chemical space. Compared to the parent neuropeptide, they display improved hydrogen bonding profiles and optimized lipophilicity, suggesting enhanced membrane permeability and developability.

8. References

(1) Lipinski, C. A.; Lombardo, F.; Dominy, B. W.; Feeney, P. J. Experimental and computational approaches to estimate solubility and permeability in drug discovery and development settings. *Adv. Drug Deliv. Rev.* **1997**, *23* (1), 3-25.

(2) Veber, D. F.; Johnson, S. R.; Cheng, H.-Y.; Smith, B. R.; Ward, K. W.; Kopple, K. D. Molecular properties that influence the oral bioavailability of drug candidates. *J. Med. Chem.* **2002**, *45* (12), 2615-2623.
