# Supplementary figures and images for: Dual-Acting Vitamin B3‑Melanostatin Neuropeptide Hybrids as Potent Modulators of the Dopamine D2 Receptors with Neuroprotective Activity
Source: J Med Chem. 2026 Jun 18;69(13):15597–613. doi: 10.1021/acs.jmedchem.6c00697 (PMC13370884; doi:10.1021/acs.jmedchem.6c00697)

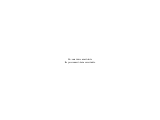

Supplement: Supplementary file 2 [file jm6c00697_si_002.zip › NMR/Compound 4a/DEPT-135/pdata/1/thumb.png]

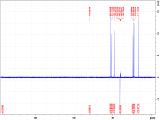

Supplement: Supplementary file 2 [file jm6c00697_si_002.zip › NMR/Compound 4d/DEPT-135/pdata/1/thumb.png]

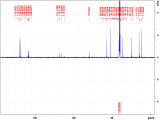

Supplement: Supplementary file 2 [file jm6c00697_si_002.zip › NMR/Compound 4e/13C/pdata/1/thumb.png]
