# Supplementary material for: Dual-Acting Vitamin B3‑Melanostatin Neuropeptide Hybrids as Potent Modulators of the Dopamine D2 Receptors with Neuroprotective Activity
Source: J Med Chem. 2026 Jun 18;69(13):15597–613. doi: 10.1021/acs.jmedchem.6c00697 (PMC13370884; doi:10.1021/acs.jmedchem.6c00697)
Supplement: Supplementary file 2 [file jm6c00697_si_002.zip › NMR/Compound 6e/13C/pdata/1/email_EB_BL_088_1_5_1.pdf]

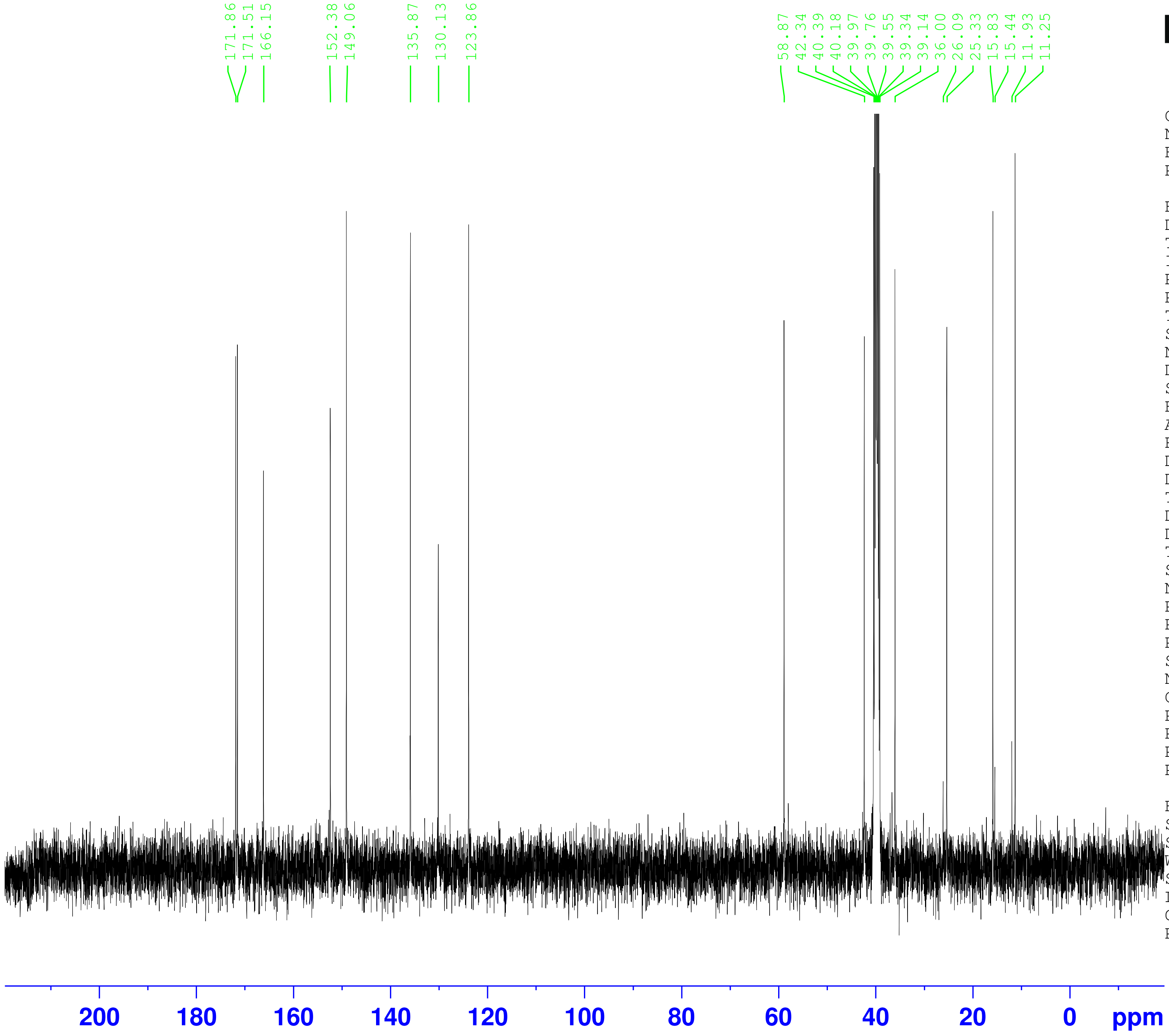

Current Data Parameters

NAME	EB_BL_088_1
EXPNO	5
PROCNO	1

F2 - Acquisition Parameters

Date_	20210915
Time	15.28 h
INSTRUM	spect
PROBHD	Z108621_0028 (
PULPROG	zgpg30
TD	65536
SOLVENT	DMSO
NS	128
DS	4
SWH	24038.461 Hz
FIDRES	0.733596 Hz
AQ	1.3631488 sec
RG	212.73
DW	20.800 usec
DE	6.50 usec
TE	298.3 K
D1	2.00000000 sec
D11	0.03000000 sec
TD0	1
SFO1	100.6253446 MHz
NUC1	13C
P0	3.07 usec
P1	9.20 usec
PLW1	50.00000000 W
SFO2	400.1416006 MHz
NUC2	1H
CPDPRG[2	waltz65
PCPD2	90.00 usec
PLW2	25.00000000 W
PLW12	0.36820000 W
PLW13	0.13140000 W

F2 - Processing parameters

SI	32768
SF	100.6152830 MHz
WDW	EM
SSB	0
LB	1.00 Hz
GB	0
PC	1.40
