# Supplementary material for: Dual-Acting Vitamin B3‑Melanostatin Neuropeptide Hybrids as Potent Modulators of the Dopamine D2 Receptors with Neuroprotective Activity
Source: J Med Chem. 2026 Jun 18;69(13):15597–613. doi: 10.1021/acs.jmedchem.6c00697 (PMC13370884; doi:10.1021/acs.jmedchem.6c00697)
Supplement: Supplementary file 2 [file jm6c00697_si_002.zip › NMR/Compound 6e/1H/pdata/1/email_EB_BL_088_1_4_1.pdf]

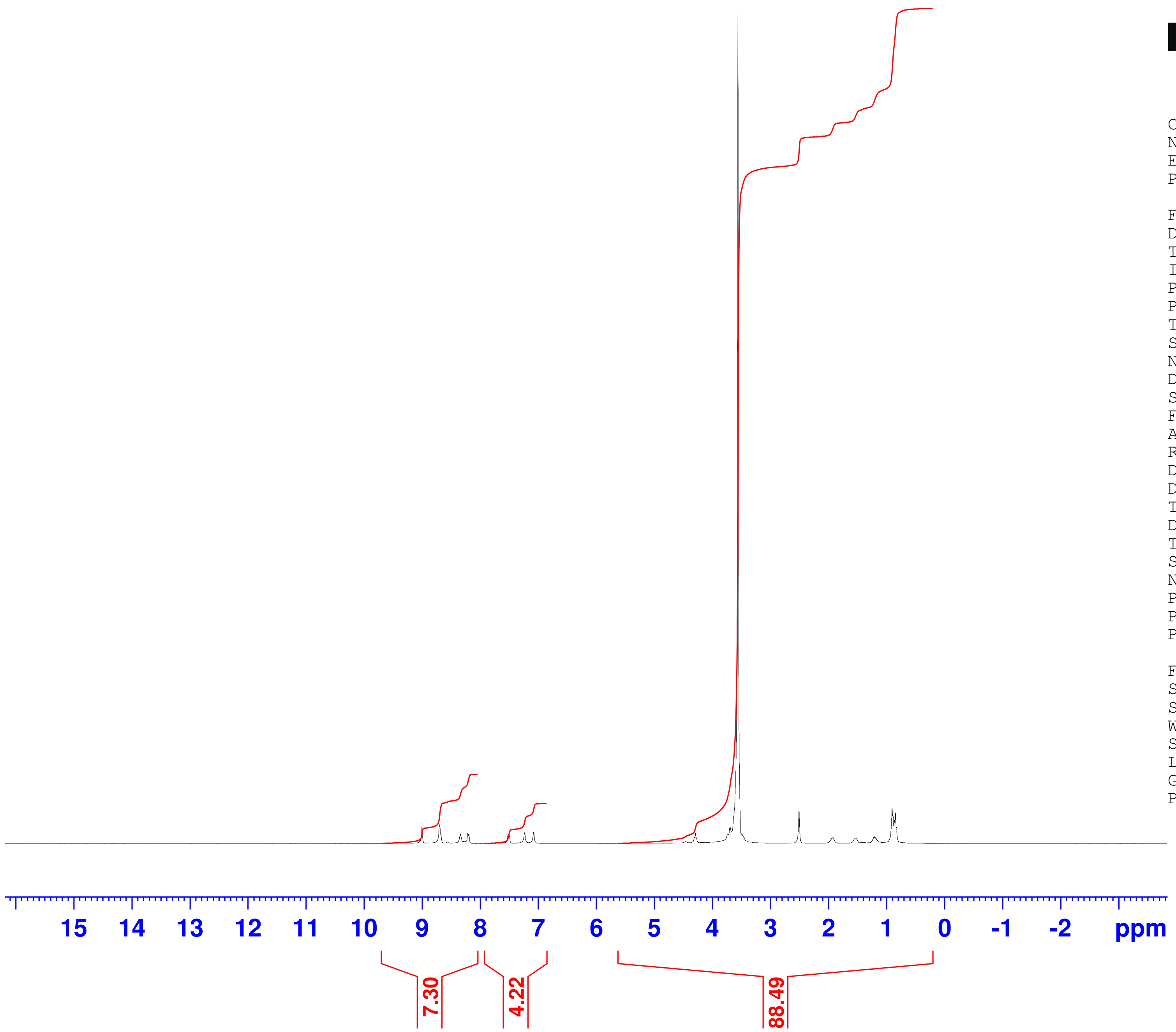

BRUKER

```
Current Data Parameters
NAME          EB_BL_088_1
EXPNO                   4
PROCNO                  1

F2 - Acquisition Parameters
Date_            20210915
Time                15.19 h
INSTRUM             spect
PROBHD    Z108621_0028 (
PULPROG              zg30
TD                  65536
SOLVENT              DMSO
NS                      2
DS                      2
SWH              8012.820 Hz
FIDRES           0.244532 Hz
AQ              4.0894465 sec
RG                  31.89
DW                 62.400 usec
DE                   6.50 usec
TE                  298.2 K
D1            1.00000000 sec
TD0                     1
SFO1          400.1424710 MHz
NUC1                   1H
P0                   3.00 usec
P1                   9.00 usec
PLW1          25.00000000 W

F2 - Processing parameters
SI                  65536
SF            400.1400000 MHz
WDW                    EM
SSB                     0
LB                   0.30 Hz
GB                      0
PC                   1.00
```
